# Nanoparticle size distribution quantification: results of a small-angle X-ray scattering inter-laboratory comparison

**DOI:** 10.1107/S160057671701010X

**Published:** 2017-08-18

**Authors:** Brian R. Pauw, Claudia Kästner, Andreas F. Thünemann

**Affiliations:** a Federal Institute for Materials Research and Testing (BAM), Unter den Eichen 87, 12205 Berlin, Germany

**Keywords:** small-angle scattering, accuracy, methodology, silver nanoparticles, poly(acrylic acid), *SASfit*, *McSAS*, inverse Fourier transform, round robin

## Abstract

This paper presents the first worldwide inter-laboratory comparison of small-angle X-ray scattering (SAXS) for nanoparticle sizing. The measurands in this comparison are the mean particle radius, the width of the size distribution and the particle concentration. The investigated sample consists of dispersed silver nanoparticles, surrounded by a stabilizing polymeric shell of poly(acrylic acid). The silver cores dominate the X-ray scattering pattern, leading to the determination of their radius size distribution using (i) the generalized indirect Fourier transformation method, (ii) classical model fitting using *SASfit* and (iii) a Monte Carlo fitting approach using *McSAS*. The application of these three methods to the collected data sets from the various laboratories produces consistent mean number- and volume-weighted core radii of *R*_n_ = 2.76 (6) nm and *R*_v_ = 3.20 (4) nm, respectively. The corresponding widths of the lognormal radius distribution of the particles were σ_n_ = 0.65 (1) nm and σ_v_ = 0.71 (1) nm. The particle concentration determined using this method was 3.0 (4) g l^−1^ or 4.2 (7) × 10^−6^ mol l^−1^. These results are affected slightly by the choice of data evaluation procedure, but not by the instruments: the participating laboratories at synchrotron SAXS beamlines, commercial and in-house-designed instruments were all able to provide highly consistent data. This demonstrates that SAXS is a suitable method for revealing particle size distributions in the sub-20 nm region (at minimum), out of reach for most other analytical methods.

## Abstract 2

An extensive round robin experiment between small-angle X-ray scattering laboratories has delivered a global uncertainty estimate for the measurands of a nanoparticle dispersion. Irrespective of the instrument pedigree, the distribution mean, width and volume fraction could be determined with an accuracy of 1, 10 and 10%, respectively.

## 1. Introduction   

Demonstrating that a given technique is truly able to reliably determine the size distribution and quantify the number of nano-objects is of great importance. Such a demonstration can be done using an inter-laboratory or ‘round robin’ comparison, comparing results inferred from measurements of identical samples on different instruments. Only a few such round robin experiments exist for the analytical methods used in nanotechnology, most notably for single-particle inductively coupled plasma mass spectrometry (ICP-MS) (Linsinger *et al.*, 2014 ▸; Montoro Bustos *et al.*, 2015 ▸) and transmission electron microscopy (Rice *et al.*, 2013 ▸). Furthermore, only one exists for small-angle neutron scattering (SANS) (Rennie *et al.*, 2013 ▸) and none at all for small-angle X-ray scattering (SAXS). SAXS is an uncomplicated bulk nanostructural quantification technique, particularly sensitive to the smaller end of the nanoscale, and therefore a prime candidate to answer the aforementioned analytical needs. Results from SAXS have repeatedly been demonstrated to agree well with findings from electron microscopy (Borchert *et al.*, 2005 ▸; Rosalie & Pauw, 2014 ▸), and comparisons between the results of two or three SAXS instruments suggest that the inter-instrument reproducibility could be satisfactory (Krumrey *et al.*, 2011 ▸; Allen *et al.*, 2017 ▸). In the absence of a standard methodology, however, a wide range of data collection and correction procedures are being applied at various laboratories and synchrotrons (Jacques & Trewhella, 2010 ▸; Pauw, 2013 ▸). The effects of these variations on the inter-laboratory reproducibility of SAXS findings are hard to estimate without a more thorough comparison.

A round robin experiment for SAXS was therefore conducted to assess its practical precision and accuracy. To this end, a suitable sample of dispersed particles was chosen that satisfies particular conditions: analyte dimensions smaller than 10 nm, limited size dispersity and with a reasonable scattering power (a combination of contrast and concentration). Suitable samples were synthesized in our laboratory in the form of poly(acrylic acid)-stabilized silver nanoparticles with nominal radii of 3 nm (Kästner & Thünemann, 2016 ▸). Silver nanoparticles are also one of the most widespread types of nanoparticles in consumer products worldwide and their accurate quantification is, therefore, of great interest (Jemec *et al.*, 2016 ▸).

This work provides the first inter-laboratory comparison of the measurement of nanoparticle size distributions with SAXS. The measurements received for this sample from the various laboratories are anonymized and subjected to a trio of fundamentally different analysis methods. On this basis, we arrive at a well founded estimate of how precise the SAXS method is for the determination of sizes of nanoparticles in the sub-20 nm range.

## 2. Experimental   

### 2.1. Explicit experiment conditions   

As the purpose of the study is to determine the practical precision of SAXS-based measurands, we explicitly refrain from comparing the instruments directly. To that end, all collected data sets have been anonymized thoroughly (details are available in the supporting information), using the anonymization procedure described below. To assess the current state of inter-instrument variability as accurately as possible, a minimum of restrictions were imposed on the participants: each laboratory and user was given a brief instruction set (see below), but was otherwise left free to choose their own measurement and data processing criteria.

### 2.2. Participants   

A total of 45 data sets were measured in 22 laboratories on 41 samples using 24 instruments (a maximum of two samples per instrument). We understand that four instruments were slit-collimated instruments and one was a Bonse–Hart instrument. Samples were measured from February to May 2016. As shown in Table 1 ▸, 18 laboratories measured both samples, of which two laboratories measured both samples on two different instruments, and one beamline measured one sample at two photon energies. Three laboratories measured one sample only.

Many of these laboratories were recruited at the 16th International Conference on Small-Angle Scattering in Berlin, while others were recruited *via* an announcement of the study on a SAXS-related weblog (http://www.lookingatnothing.com/).

### 2.3. Sample preparation   

The nanoparticle samples were synthesized according to the exact procedure described elsewhere (Kästner & Thünemann, 2016 ▸). The resulting batch of 300 ml was used to fill 60 bottles with 5 ml each. The samples were sent in labelled pairs to the individual laboratories by regular mail, encapsulated within a padded box. To ensure that the effects of mailing are minimal, a few samples have been returned after measuring and measured again to ensure their stability during transport (no measurable differences were observed).

### 2.4. Measurements   

We requested that the two samples sent to each participant should be measured in adherence to the following conditions: (i) samples should be measured undiluted as delivered over a range of 0.1 ≤ *q* (nm^−1^) ≤ 3.0, (ii) at least the water background should be subtracted, (iii) if possible, the intensity should be provided in absolute units and (iv) if possible, with uncertainty estimates of the intensity. Participants using slit-smeared instruments returned de-smeared data. Thus, the participants of this inter-laboratory study provided background-subtracted scattering curves, but were not required to perform any data evaluation.

### 2.5. Data set anonymization and post-processing   

Received data sets were anonymized and post-processed in several steps:

(*a*) The filenames were re-named to represent the sample vial number and were cleaned of any additional information.

(*b*) The units were made uniform, by converting (when necessary) to [*q*] = nm^−1^ and to 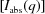 = m^−1^ for those data sets supplied with the intensity calibrated to absolute units.

(*c*) The *q* range was limited to the range specified in the measurement conditions [0.1 ≤ *q* (nm^−1^) ≤ 3]. This range is sufficiently broad to accurately describe the distribution of the chosen particles.

(*d*) The data were re-binned to a target of 100 logarithmically spaced bins using the procedure available in the supporting information (this only reduces the number of data points by averaging neighbouring points). This re-binning propagates existing uncertainty estimates when supplied, and estimates a second uncertainty estimate based on the standard error on the mean of the intensity values in the bin. The larger of these two estimates is chosen to represent the uncertainty on the intensity for each data point.

(*e*) The resulting uncertainty is limited to a minimum of 1% of the data point intensity value, which is an appropriate lower limit for SAXS (Bressler, Pauw & Thünemann, 2015 ▸). This is done to avoid disproportionate data point weighting differences in the fitting procedures due to unrealistically low uncertainty estimates. Of all the re-binned data points in this study, 31.4% were affected by this lower limit.

Through this procedure, it is unlikely that another laboratory can be identified by their data set. Since the data sets are named by their sample vial numbers, each laboratory can positively identify (only) their own data set. The choice as to whether to reveal their ‘identity’ is thus left up to the wishes of the individual laboratories. Note that the anonymized data sets are made available under a Creative Commons licence for further scrutiny by interested parties (see the supporting information).

### 2.6. Analyses   

**Figure 1:**
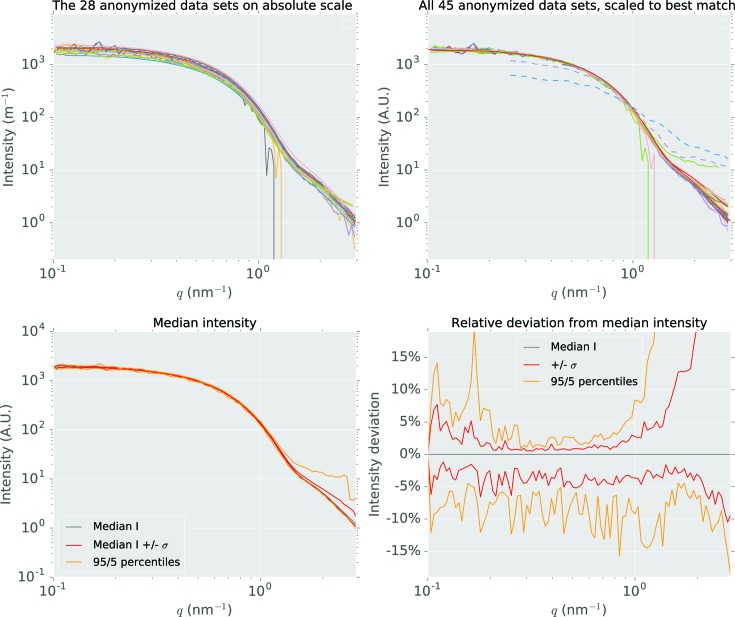
Top left: the subset of 28 post-processed data sets provided in absolute units of silver particles as a function of the scattering vector. 0.45% of the data points are negative and therefore not shown because of the logarithmic intensity scale. Top right: overlay of all 45 SAXS data sets provided by the participants (*i.e.* those provided in absolute as well as arbitrary intensity units). For visual comparison, the data sets are matched using an uncertainty-weighted least-squares procedure to optimize the scaling factor (see *Jupyter Notebook* in the supporting information). The dashed curves are considered outliers of the study. 0.25% of the data points are negative and therefore cannot be displayed. Bottom left: median intensity of the 45 data sets, scaled to best match, median ±34.1% percentiles (in total two standard deviations, red) and median ±45% percentiles (within which 90% of the data lie, orange). Bottom right: the percentiles shown relative to the median intensity, showing a 5–10% deviation of the intensity of the curves within two standard deviations (red). Error bars are not shown in any sub-figure for clarity.

While differences in the intensity values between the laboratories’ measurements are somewhat interesting from a metrological perspective (see Fig. 1 ▸), the practical effects of these differences on the derived parameters are more relevant to the user. In this study, the following measurands could be assessed: (i) the mean radius, (ii) the width of the size distribution and (iii) the particle concentration. However, these measurands may be biased by the chosen data analysis procedure.

To find out, we chose typical representatives of three fundamentally different evaluation methods for determination of the measurands: (i) the GIFT, implementing an indirect Fourier transformation (IFT) (Glatter, 1980 ▸), (ii) *SASfit*, implementing a model fit of spheres (Pedersen, 1997 ▸) with a lognormal size distribution, and (iii) *McSAS*, implementing a Monte Carlo determination of size distributions assuming spherical scatterers (Bressler, Pauw & Thünemann, 2015 ▸). Other methods such as that developed by Sen *et al.* (2014 ▸) or usage of the mature evaluation package *IRENA* (Ilavsky & Jemian, 2009 ▸) are also suitable, but an exhaustive comparison of all available data evaluation methods and packages is beyond the scope of this study.

For all three of the above methods, the model assumes dilute, non-interacting spherical particles of non-uniform size. In the case of *SASfit*, the size distribution form is further restricted to a lognormal representation of the number-weighted distribution. For the GIFT, a non-negativity and smoothness constraint is applied to the size distribution, and the distribution parameters are determined by fitting a log­normal function to the IFT results. For *McSAS*, only a non-negativity constraint is applied to the size distribution with the population parameters directly calculated from the resulting set. A flat background is included in the fitting procedures. To aid reproducibility, a software usage guide (SUG) has been defined for each of the analysis methods to analyse the data sets of this study. These SUGs are provided in the supporting information.

## 3. Results and discussion   

### 3.1. Overview of the returned data sets   

The laboratory procedures for performing SAXS measurements varied greatly between participants, for example in their choice of sample containers: some used re-usable containers or flow-through capillaries, whereas others used non-identical containers for the sample and background measurements. Likewise, a wide spectrum of data correction procedures (Pauw, 2013 ▸), from very basic to very advanced, were employed. Differences in both collection and correction procedures can affect the data. Our data anonymization and post-processing procedure also risks affecting the data, but no significant effects were found after extensive testing. Of note is that 31.4% of the resulting data points had uncertainty estimates smaller than 1% of the intensity, indicating that there is a tendency to underestimate the uncertainty in the incoming data sets. This is further shown by the necessity for the analysis procedures to raise the convergence criterion above 1 (see Table S1 in the supporting information) for about half of the data sets, also highlighting that the expanded uncertainty estimation and limits in our post-processing procedures do not affect the uncertainties far enough.^1^

Nonetheless, the received, pre-processed data show a high degree of similarity when plotted on a double-logarithmic scale, as evident from the scattering curve comparisons in Fig. 1 ▸. Note that, for all the comparisons except the top-left figure, the curves were matched to each other using an uncertainty-weighted least-squares procedure to optimize the scaling factors (this scaling has not been used further in the data analysis). The procedures to achieve this, as well as additional information on the data sets, are available in the *Jupyter Notebook* provided in the supporting information. The similarity of the data sets is best evaluated from the relative deviations shown as percentiles, demonstrating that the intensity can easily deviate by ±5%. The effect of these deviations on the resulting morphological parameters will be investigated below.

Most importantly, the samples, which contain 14 wt% of poly(acrylic acid) as stabilizer, have been found to be highly resistant to synchrotron radiation.^2^ Secondly, no time-correlation effect was observed in the samples for the duration of the comparison, demonstrating that the sample was stable throughout the experiment, and resilient to the environmental changes encountered during shipping.

### 3.2. Data analysis using IFT   

The agreement between the measurands obtained from the different data sets needs to be assessed using the various data analysis procedures, starting with the IFT. This method was developed by Glatter around 1980, and provides a convenient approach to determine intensity, volume- and number-weighted particle distributions (Glatter, 1977 ▸, 1980 ▸). The method has seen some updates and is still in widespread use (Pedersen, 1999 ▸). It is used in this study to determine the number- and volume-weighted radius distributions, following the SUG in the supporting information.

**Figure 2:**
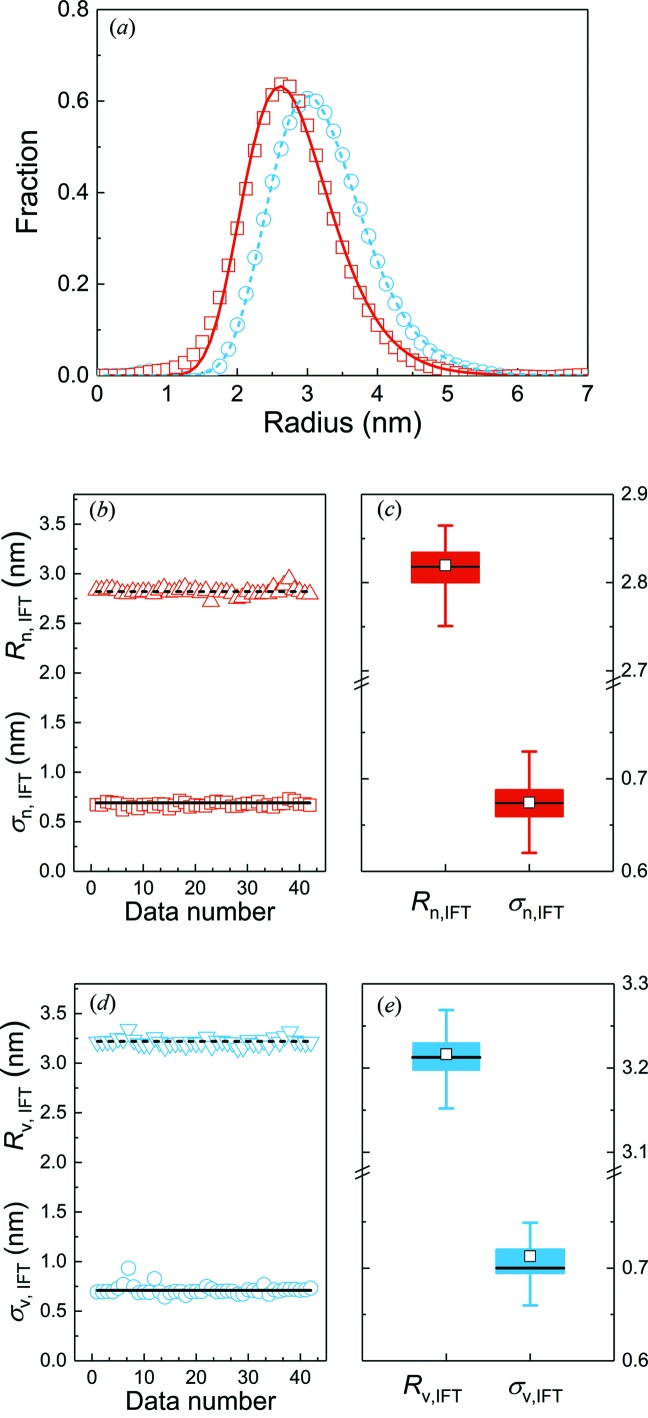
Results of data evaluation using the IFT method (Glatter, 1980 ▸). (*a*) Volume- and number-weighted radii distribution derived from data set number 2, shown as blue and red lines, respectively. (*b*) Number-weighted radii, *R*_n,IFT_, and widths of the size distribution, σ_n,IFT_, as a function of the data set number (triangles and squares, respectively). Mean values of the data sets *R*_n,IFT_ = 2.82 (4) nm and σ_n,IFT_ = 0.67 (2) nm are shown as horizontal lines. (*c*) Box plot depicting the distribution of *R*_n,IFT_ and σ_n,IFT_ from the measurements. The horizontal line that forms the top of the box is the 75th percentile (*Q*_1_). The horizontal line that forms the bottom is the 25th percentile (*Q*_3_). The horizontal line within the box is the median value and the square corresponds to the mean value. The whiskers represent lower 5 and 95% values. (*d*) Volume-weighted radii, *R*_v,IFT_, and widths of the size distribution, σ_v,IFT_, as a function of the data set number (triangles and spheres, respectively). Mean values of the data sets *R*_v,IFT_ = 3.22 (4) nm and σ_v,IFT_ = 0.71 (5) nm are shown as horizontal lines. (*e*) Box plot of the distribution of *R*_v,IFT_ and σ_v,IFT_ from the measurements. Results are summarized in Table S2.

As the population modes are not automatically provided by the IFT, they are determined by fitting a distribution function to the result, and so an appropriate distribution function must be selected. The IFT-resultant distributions are slightly asymmetric around their maxima, with the tail decaying more slowly towards larger radii, as shown for one data set in the upper part of Fig. 2 ▸. Therefore, symmetric functions such as a Gaussian profile cannot be considered for their description, but a lognormal function describes the distributions sufficiently well. The choice of a lognormal distribution is, furthermore, supported by theoretical considerations (Kiss *et al.*, 1999 ▸) and a transmission electron microscopy inter-laboratory study on nominally 30 nm NIST gold nanoparticles (Rice *et al.*, 2013 ▸), and is recommended for the standardization of the classification of magnetic nanoparticle systems (Bogren *et al.*, 2015 ▸).

Here we employed the lognormal distribution of the radii, *R*, defined as 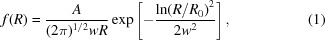
with *A* the area of the size distribution, *w* the scale parameter defining the width of the size distribution and *R*_0_ the median radius, which is the value of the radii in the limit of *w =* 0. The mean value for the radii of the lognormal distribution is defined by 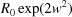 and its standard deviation 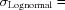 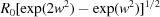. Examples of the fits of the distribution function to the number- and volume-weighted IFT results are shown in Fig. 2 ▸(*a*) (red and blue lines, respectively).

This procedure was carried out for all received data sets, minus the two outliers (Nos. 6 and 16), in order to retrieve the number-weighted mean radii, *R*_n,IFT_, and mean widths, σ_n,IFT_ (Fig. 2 ▸ *b*). The mean values of the data sets are *R*_n,IFT_ = 2.82 (4) nm and σ_n,IFT_ = 0.67 (2) nm. The null hypothesis that both *R*_n,IFT_ and σ_n,IFT_ are distributed according to a Student’s *t* distribution is not rejected at the 0.05 level. The box plot of these two (Fig. 2 ▸ *c*) highlights that 90% of the values for the radii are within the range 2.81 ≤ *R*_n,IFT_ (nm) ≤ 2.83 and the widths are within 0.67 ≤ σ_n,IFT_ (nm) ≤ 0.68. Therefore, the spread of the radii on a 90% interval is within 0.1 nm. This is surprisingly low given the relative breadth of the distribution of our particles of around 20%, in particular when compared with typical proteins or monodisperse latex particles (Rennie *et al.*, 2013 ▸).

We repeated the IFT data evaluation procedure for the determination of the volume-weighted radii and found mean values of *R*_v,IFT_ = 3.22 (4) nm and σ_v,IFT_ = 0.71 (5) nm (Fig. 2 ▸ *d*). The box plots in Fig. 2 ▸(*e*) show that 90% of the values for the radii are within the range 3.20 ≤ *R*_v,IFT_ (nm) ≤ 3.23 and the widths are within 0.70 ≤ σ_v,IFT_ (nm) ≤ 0.73. Again, the spread of the values on a 90% interval is within 0.1 nm. The volume-weighted radii are significantly larger than the number-weighted ones owing to the breadth of the size distribution (for monodisperse size distributions, *R*_n_ = *R*_v_).

This precision of the determined radii is surprising for a size-disperse sample. It is known that SAXS can provide precise radii if the particle size distribution is narrow, *i.e.* if the width of the particle size distribution can be neglected (Borchert *et al.*, 2005 ▸). This was demonstrated by a SANS round robin test on 77 nm large latex particles with a very narrow size distribution (Rennie *et al.*, 2013 ▸). They found that the spread in the fitted mean particle size was about ±1%, but the uncertainties in the determination of the size distribution were much larger and sensitive to a number of instrumental effects. We now find that a similarly high precision in the radius determination can also be achieved for nanoparticles with a broader size distribution (with a width of about 20%; Fig. 2 ▸). As a result, we conclude that the IFT evaluation is ostensibly insensitive to the variations between (i) the participants’ data sets and (ii) their instruments. However, the IFT method does impose a smoothness constraint on the resulting size distribution, which may artificially constrict the results and thereby introduce an overestimated degree of precision. In the next step we therefore investigate the influence of the choice of data evaluation procedure on the results.

### 3.3. Comparison of IFT with representatives of other methods   

**Figure 3:**
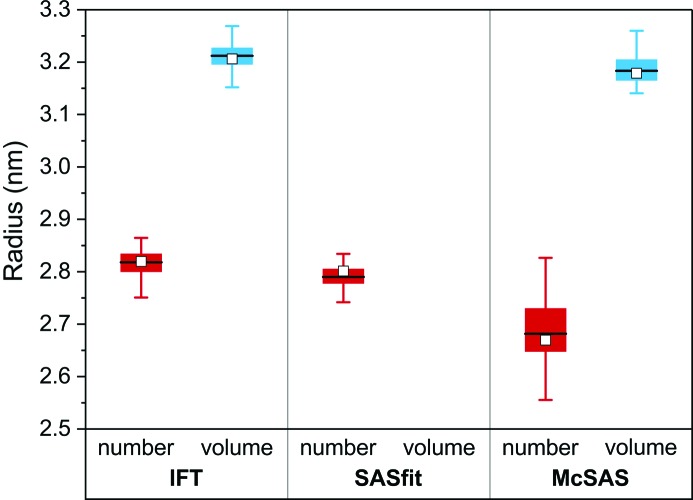
Comparison of number- and volume-weighted radii derived from IFT and the *SASfit* and *McSAS* programs. Number-weighted values are in red, volume-weighed in blue. The top and bottom of the box delineate the 75th (*Q*_1_) and 25th (*Q*_3_) percentiles. The horizontal line within the filled box is the median value and the square represents the mean value. The whiskers correspond to lower 5 and 95% limits. Data are listed in Table S2.

We used *SASfit* (Breßler, Kohlbreche & Thünemann, 2015 ▸) as a representative of a classical curve-fitting procedure and *McSAS* (Bressler, Pauw & Thünemann, 2015 ▸) as a Monte Carlo fitting program (a minimal assumption method), to compare with the aforementioned IFT results. The results obtained from both for the radii and widths are visually summarized in the curves and box plots of Figs. 3 ▸ and 4 ▸, respectively. All values are listed in Table 2 ▸, with more detail in Table S2. Note that *SASfit* only provides estimates of number-weighted size distributions in its current implementation and does not provide volume-weighted distributions (Breßler, Kohlbrecher & Thünemann, 2015 ▸). We have chosen the lognormal distribution in *SASfit* for the stated reasons.

Figs. 3 ▸ and 4 ▸ show that the means of the radii and widths are similar for all three evaluation methods (means are indicated by white squares in the box plots). In order to test whether the mean values resulting from the IFT, *SASfit* and *McSAS* methods are the same we employed analysis of variance (ANOVA) at the 0.05 level. This demonstrates firstly that the number-weighted mean radii *R*_n,IFT_, *R*_n,*SASfit*_ and *R*_n,*McSAS*_ are not equal [with a data mean of 2.76 (6) nm]. Secondly, the volume-weighted mean radii *R*_v,IFT_ and *R*_v,*McSAS*_ [with a mean of 3.20 (4) nm] show a very small, yet still significant, difference according to ANOVA. Thirdly, we found that the number-weighted mean widths σ_n,IFT_, σ_n,*SASfit*_ and σ_n,*McSAS*_ are significantly different [data mean is 0.65 (1) nm]. Lastly, however, the volume-weighted mean widths of σ_v,IFT_ and σ_v,*McSAS*_ are equal [data mean is 0.71 (1) nm]. The ANOVA underscores that the values for *R*_n_, *R*_v_ and σ_n_ are dependent on the type of evaluation method we used in this study. In contrast, σ_v_ is (perhaps by chance) independent of the choice of the method. Of interest is that the spread of the *R*_n_, *R*_v_, σ_n_ and σ_v_ values is somewhat smaller for IFT and *SASfit* in comparison with *McSAS* (see Figs. 3 ▸ and 4 ▸). An overview of their interquartile ranges is given in Table S2, where it can be seen that they are 0.03 nm (IFT), 0.02–0.03 nm (*SASfit*) and 0.04–0.08 nm (*McSAS*). The primary cause of this difference is likely to be the increased number of assumptions (restrictions) applied in the IFT and *SASfit* methods.

The values of the interquartile ranges for all three methods are small enough for us to recommend all three for data evaluation purposes. The highly consistent results of the IFT method indicate that it is the best suited method for this particular kind of problem. The relatively wide interquartile ranges of *McSAS* result from its form-free nature, *i.e.* no assumption is made on the type, modality or smoothness of the size distribution. Therefore, we recommend a preferential use of one of the programs depending on the prior knowledge of the particle system under investigation. The IFT should be the first choice if it is known that the particle size distribution is smooth, while *McSAS* is the first choice if little *a priori* knowledge is available.^3^ The use of *SASfit* is recommended if an estimate of the size distribution form is known, since it provides more than 20 different size distributions (Breßler, Kohlbrecher & Thüneman, 2015 ▸). In ambiguous situations we recommend comparing the results from the different methods to verify the results.

### 3.4. Accuracy and precision limits of the particle size distribution   

The estimation of the precision and accuracy of nanoparticle size distributions, referring to the closeness of agreement and the distance to the true values, respectively, is inherently challenging for a wide range of nanoscale sizing techniques. These problems arise because the outcome of particle sizing of these dimensions is generally method specific, as discussed in a *post hoc* inter-laboratory comparison by Montoro Bustos *et al.* (2015 ▸). In this context, SAXS and SANS have clear benefits in that they are fully traceable methods, based on first-principle physics, and are capable of measuring *in situ* size distributions of nanoparticles in the full nanoscale range of 1–100 nm. In principle, then, we should be able to achieve precise and accurate results.

While this work mainly details the inter-instrument variability of the findings, it is good to contrast this with the ultimately achievable accuracy and precision for a given instrument. For the determination of radii and their distributions, this means we are sensitive to variations in *q*. We have, therefore, evaluated the worst-case precision and accuracy limits of *q* for our own instrument (an Anton Paar SAXSess). This evaluation, discussing most effects affecting the *q* precision, is supplied in full in the supporting information as a modifiable *Jupyter Notebook*. This considers both the geometrical contributors to uncertainty (beam divergence, beam width, beam height, pixel or bin width, and polychromaticity) and the practically determinable accuracy using three different calibrants. In the following paragraphs, only the most important findings are summarized.

For our (slit-collimated) instrument, by far the biggest potential contributor to *q* uncertainty is the divergence due to the focusing optics. The evaluation of its possible effects, however, is complicated by the use of de-smearing, which may partially compensate for the divergence effects as a side effect to its slit-width compensation functionality. Barring that, the binning introduces the second-worst uncertainty contribution to *q*, introducing an uncertainty of maximally 3.5% of its value (full width, *cf.* the supporting information). Evaluating the effect of this worst-case shift in *q* on the *McSAS*-retrieved distribution demonstrates that a systematic binning-induced *q*-uncertainty shift can affect the found distribution means and widths by −1/+2% and −8/+6%, respectively.

Practical calibrants, such as apoferritin and silver behenate (Gilles *et al.*, 1998 ▸; Blanton *et al.*, 2000 ▸), showed a possible practical uncertainty in *q* of ±0.035 nm^−1^. Such an uncertainty in *q* can maximally affect the found distribution means and widths by 2.5 and 35%, respectively. It was demonstrated that our instrument accuracy is well within expected limits and therefore we have high confidence in the absolute radius values.

Uncertainties in the data point *q* values are typically neglected owing to their small magnitude. Our estimates show that they can significantly affect the deduced measurands, despite their small magnitude. The effect on the measurands approaches the same order of magnitude as the practical spread found between laboratories, and is, therefore, not negligible. Thus, we strongly recommend starting to consider *q* uncertainty in order to improve intercomparability and achieve ultimate nanometrological precision.

### 3.5. Particle concentration   

The particle concentration can be determined from SAXS data if the scattering intensities are provided on an absolute scale (Glatter & Kratky, 1982 ▸). This can be achieved using water (Orthaber *et al.*, 2000 ▸) or glassy carbon (Zhang *et al.*, 2010 ▸) as primary or secondary absolute calibration intensity standards. Upon the provision of data scaled to absolute units, *SASfit* (Breßler, Kohlbrecher & Thünemann, 2015 ▸) provides an estimate of particle number concentrations, which can be converted to a particle mass concentration. *McSAS* (Bressler, Pauw & Thünemann, 2015 ▸) provides estimates of volume fractions, which can be directly converted to mass concentrations. The IFT method (Fritz & Glatter, 2006 ▸) does not return any measure of particle concentration.

The intensities are given in units of 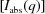 = (m sr)^−1^ and the scattering length density difference between particles and solvent in units of 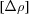 = Å^−2^. The scattering contrast was calculated to be 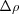 = 6.8 × 10^−5^ Å^−2^ for silver in water, as calculated for an energy of 8 keV (although specific contrast values were used for laboratories employing deviating energies). Twenty-eight data sets were provided in absolute units (labelled red in Table S1), and the resultant volume concentrations multiplied with the bulk density of silver of 10.49 g cm^−3^ to attain mass concentration estimates of the silver nanoparticles.

**Figure 5:**
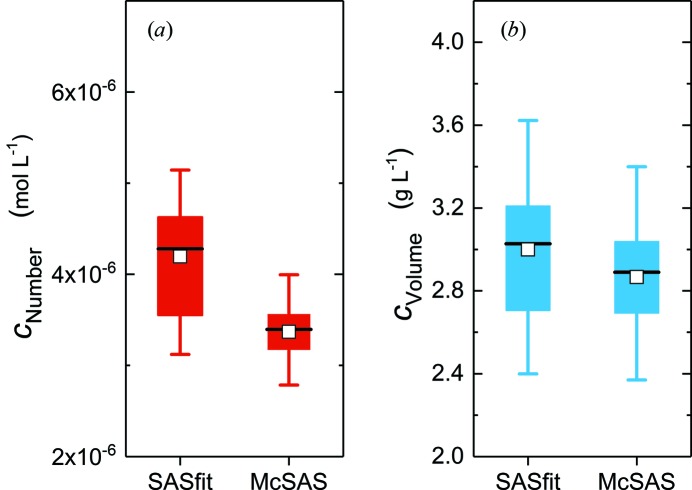
(*a*) Particle number concentration from *SASfit* and *McSAS*, with the latter converted from the volume concentration. (*b*) Particle mass concentration as converted from the *SASfit* number concentration in (*a*) and as a direct determination from *McSAS*. The white squares and horizontal lines in the box charts are the mean and median values, respectively. The lower and upper values of the box represent the quartiles *Q*_1_ and *Q*_3_; the upper and lower whiskers are the 5 and 95% levels. All values are summarized in Table S3.

The number-weighted concentrations from *SASfit* and volume-weighted concentrations from *McSAS* are summarized in Fig. 5 ▸, Table 3 ▸ and (extended) Table S3. The mean number-weighted concentration was *c*_n,*SASfit*_ = 4.20 (73) × 10^−6^ mol l^−1^ and the mean volume-weighted concentration was *c*_v,*McSAS*_ = 2.86 (31) g l^−1^. Conversion of the number concentration to volume concentration results in *c*_v,*SASfit*_ = 3.00 (38) g l^−1^. An ANOVA test shows that the *c*_v,*McSAS*_ and *c*_v,*SASfit*_ means are not significantly different at the 0.05 level. The conversion of the volume-weighted concentration *c*_v,*McSAS*_ to the corresponding number-weighted distribution results in *c*_n,*McSAS*_ = 3.37 (37) ×10^−6^ mol l^−1^. An ANOVA test shows that the means of *c*_n,*SASfit*_ and *c*_n,*McSAS*_ are significantly different. This demonstrates that, while it is possible to convert the number-weighted concentrations to volume-weighted ones, it is in general not recommended to convert the volume-weighted concentrations to number-weighted ones due to the divergence of the numerical nature of this operation. This has been discussed elsewhere (Bressler, Pauw & Thünemann, 2015 ▸).

Both analyses deliver mutually consistent values for the particle concentration and are equally useful for this challenge. Other methods, such as ICP-MS, determine the total silver content (particle-bound as well as ionic), which would be less representative of the particle concentration. On the basis of the aforementioned results, quantification of the concentration of nanoparticles with SAXS can be done straightforwardly with an uncertainty of approximately 10%.

## 4. Related literature   

For literature related to the supporting information, see Fritz & Bergmann (2006 ▸), Gleber (2013 ▸), Leiterer *et al.* (2008 ▸), Zhou *et al.* (2005 ▸).

## 5. Conclusion   

Our inter-laboratory comparison demonstrates that SAXS is a mature method for particle size analysis: accurate and precise nanoparticle sizes and size distributions can be measured irrespective of the type of instrument used, be they 0.6 or 60 m in length. SAXS reliably delivers the concentration as well as the size distribution parameters with a sub-nanometre precision. We were able to confirm that SAXS is a suitable laboratory-independent reference method for *in situ* nanoparticle analysis, reinforcing our opinion that SAXS is an appropriate technique for standardization and regulatory purposes regarding nanoparticle size analysis. This conclusion holds at least for monomodally distributed particles in suspension, but we expect a similar outcome for multimodal distributions or embedded nano-objects (a test to be performed in the future).

## Supplementary Material

A Jupyter notebook and associated files for estimating the Q uncertainty contributions of an instrument (included in the main SI document).. DOI: 10.1107/S160057671701010X/ge5041sup1.zip

Main SI document containing much of the background information.. DOI: 10.1107/S160057671701010X/ge5041sup3.pdf

The 45 anonymized datasets and an accompanying Jupyter notebook used to interpret the intensity and McSAS analyses.. DOI: 10.1107/S160057671701010X/ge5041sup2.zip

## Figures and Tables

**Figure 4:**
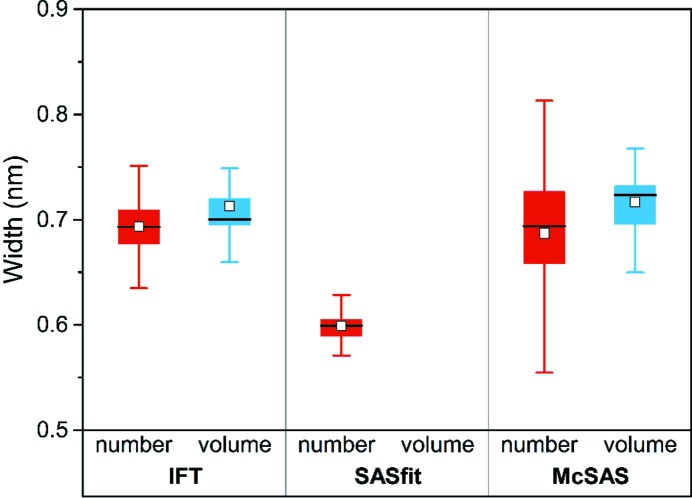
Comparison of number- and volume-weighted widths of the radius distributions derived from IFT and the *SASfit* and *McSAS* programs. Number-weighted values are in red, volume-weighed in blue. The top and bottom of the box delineate the 75th (*Q*_1_) and 25th (*Q*_3_) percentiles. The horizontal line within the filled box is the median value and the square symbol is the mean value. The whiskers represent the 5 and 95% confidence intervals. Data are given in Table S2.

**Table 1:** Tabulation of the origin of the received data sets

Number of laboratories	Measured samples	Number of instruments	Subtotal
16	2	1	32
2	2	2	8
1	1	2	2
3	1	1	3
		Total measurements	45

**Table 2:** Summary of the statistical data of the inter-laboratory comparison (*cf.* Table S2)

	IFT				*SASfit* †			*McSAS*			
Weighting	Number		Volume		Number			Number		Volume	
Statistical value	*R*_n,IFT_ (nm)	σ_n,IFT_ (nm)	*R*_v,IFT_ (nm)	σ_v,IFT_ (nm)	*R*_n,*SASfit*_ (nm)	σ_n,*SASfit*_ (nm)		*R*_n,*McSAS*_ (nm)	σ_n,*McSAS*_ (nm)	*R*_v,*McSAS*_ (nm)	σ_v,*McSAS*_ (nm)
Mean	2.82	0.67	3.22	0.71	2.80	0.60		2.67	0.69	3.18	0.72
Width, SD	0.04	0.02	0.04	0.05	0.07	0.02		0.16	0.08	0.07	0.06

† The *SASfit* software provides estimates of the number-weighted particle properties only.

**Table 3:** Summary of concentration determination of the statistical data of the inter-laboratory comparison with *SASfit* and *McSAS* (*cf.* Table S2; the current implementation of IFT does not provide an estimate of the concentration)

	*SASfit*		*McSAS*	
Weighting	Number	Volume†	Number‡	Volume
Statistical value	*c*_n,*SASfit*_ (10^−6^ mol l^−1^)	*c*_v,*SASfit*_ (g l^−1^)	*c*_n,*McSAS*_ (10^−6^ mol l^−1^)	*c*_v,*McSAS*_ (g l^−1^)
Mean	4.20	3.00	3.37	2.86
Width, SD	0.73	0.38	0.37	0.31

† The *SASfit* software provides estimates of the number-weighted particle properties. Volume-weighted concentrations were calculated by conversion of number-weighted values.

‡ The *McSAS* software provides volume-weighted concentrations. Number-weighted concentration was calculated by conversion of volume-weighted values from *McSAS*.

## Footnotes

1 A future improvement of such a round robin study can apply more stringent conditions on the data corrections and provide a universally applicable procedure for the uncertainty estimation. With this, a more detailed evaluation of the uncertainties would be possible.

2 It should be noted that silver particles with 4 wt% stabilizer were used in an earlier attempt to perform this inter-laboratory comparison, but aggregated rapidly when irradiated with synchrotron radiation (of the order of 10^12^ photons s^−1^). That first attempt started showing a clear time-dependent drift of the incoming SAXS data three months into the inter-laboratory comparison, which led to its abortion. The particles’ radiation stability will be discussed in a separate publication.

3 For example, multimodal size distributions can be detected easily with *McSAS*, as has been demonstrated for the reference material ERM-FD-102 (a suspension of bimodal silica particles) (Bressler, Pauw & Thünemann, 2015 ▸).
