# Supplementary material for: Nanoparticle size distribution quantification: results of a small-angle X-ray scattering inter-laboratory comparison
Source: J Appl Crystallogr. 2017 Aug 18;50(Pt 5):1280–8. doi: 10.1107/S160057671701010X (PMC5627679; doi:10.1107/S160057671701010X)
Supplement: Supplementary file 1 [file j-50-01280-sup1.zip › QPrecision/data/exDminus0p012 2016-11-10_15-01-35/exDminus0p012 2016-11-10_15-01-35.pdf]

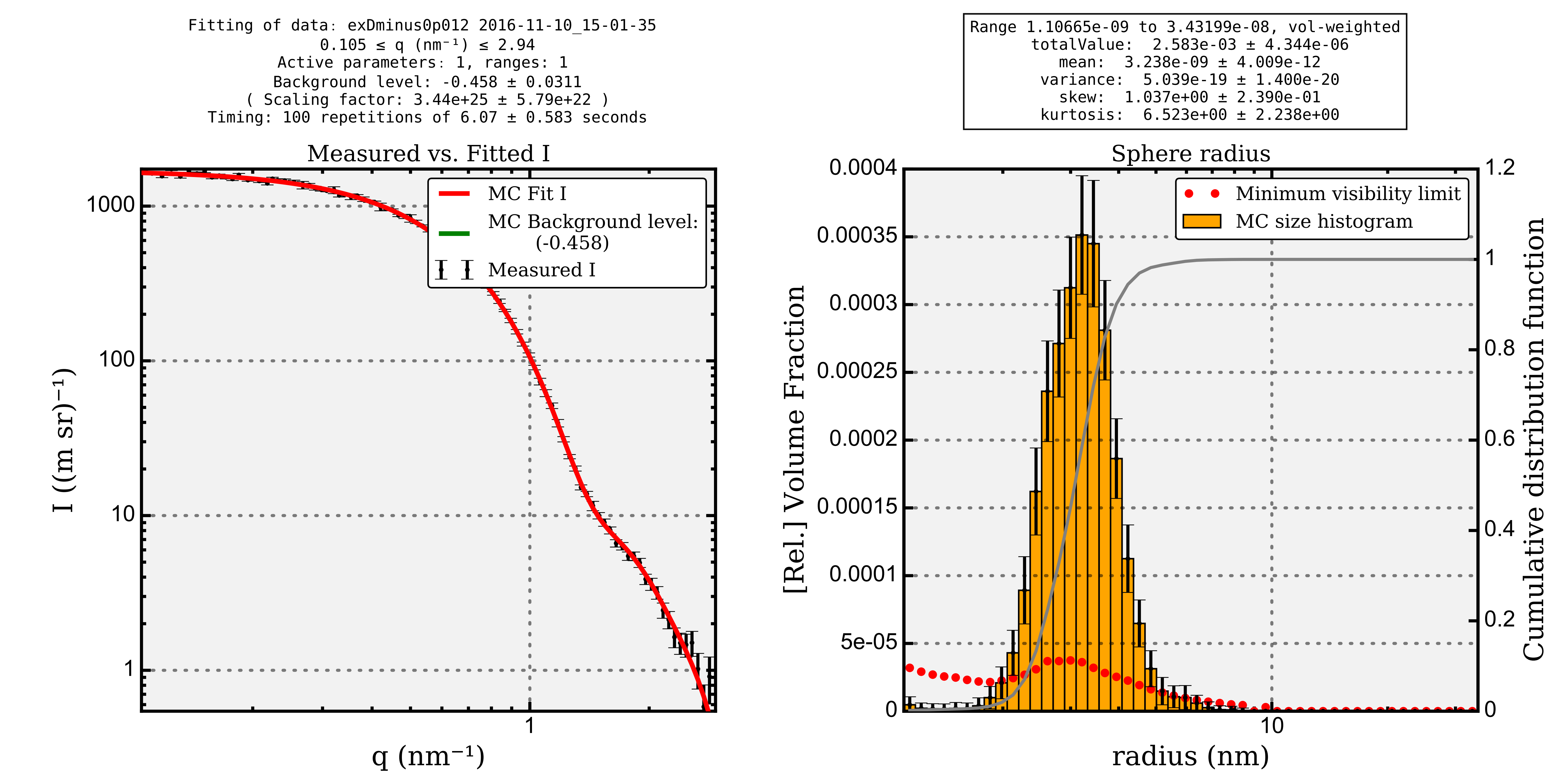

Fitting of data: exDminus0p012 2016-11-10_15-01-35
0.105 ≤ q (nm⁻¹) ≤ 2.94
Active parameters: 1, ranges: 1
Background level: -0.458 ± 0.0311
(Scaling factor: 3.44e+25 ± 5.79e+22)
Timing: 100 repetitions of 6.07 ± 0.583 seconds
Range 1.10665e-09 to 3.43199e-08, vol-weighted
totalValue: 2.583e-03 ± 4.344e-06
mean: 3.238e-09 ± 4.009e-12
variance: 5.039e-19 ± 1.400e-20
skew: 1.037e+00 ± 2.390e-01
kurtosis: 6.523e+00 ± 2.238e+00
Measured vs. Fitted I
MC Fit I
MC Background level: (-0.458)
Measured I
I ((m sr)⁻¹)
1000
100
10
1
q (nm⁻¹)
Sphere radius
Minimum visibility limit
MC size histogram
[Rel.] Volume Fraction
0.0004
0.00035
0.0003
0.00025
0.0002
0.00015
0.0001
5e-05
0
radius (nm)
10
Cumulative distribution function
1.2
1
0.8
0.6
0.4
0.2
0
