# Supplementary material for: Nanoparticle size distribution quantification: results of a small-angle X-ray scattering inter-laboratory comparison
Source: J Appl Crystallogr. 2017 Aug 18;50(Pt 5):1280–8. doi: 10.1107/S160057671701010X (PMC5627679; doi:10.1107/S160057671701010X)
Supplement: Supplementary file 1 [file j-50-01280-sup1.zip › QPrecision/data/exDminus0p035 2016-11-15_09-59-45/exDminus0p035 2016-11-15_09-59-45.pdf]

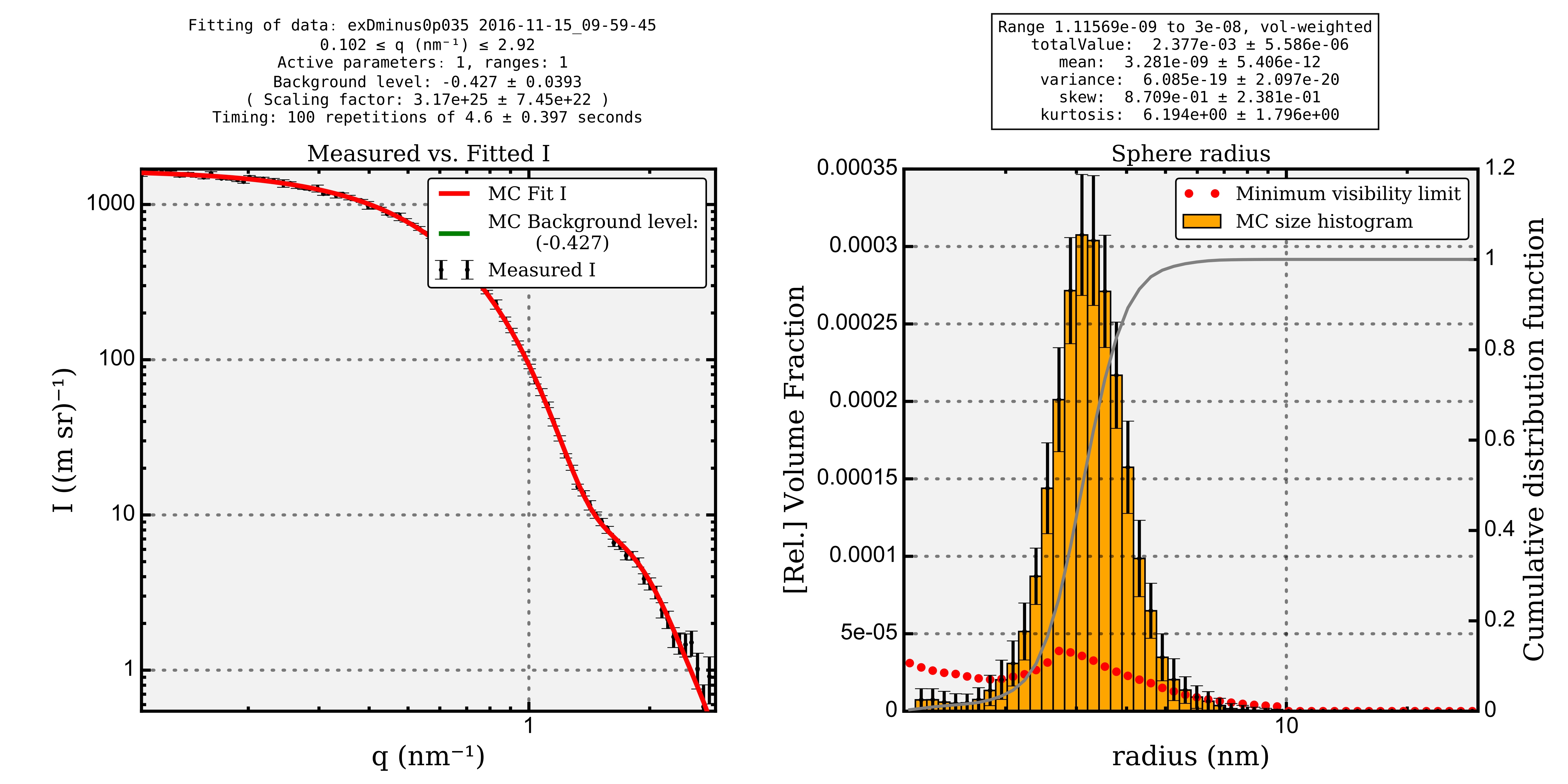
Fitting of data: exDminus0p035 2016-11-15_09-59-45
0.102 ≤ q (nm⁻¹) ≤ 2.92
Active parameters: 1, ranges: 1
Background level: -0.427 ± 0.0393
(Scaling factor: 3.17e+25 ± 7.45e+22)
Timing: 100 repetitions of 4.6 ± 0.397 seconds
Measured vs. Fitted I
MC Fit I
MC Background level: (-0.427)
Measured I
I ((m sr)⁻¹)
1000
100
10
1
q (nm⁻¹)
1
Range 1.11569e-09 to 3e-08, vol-weighted
totalValue: 2.377e-03 ± 5.586e-06
mean: 3.281e-09 ± 5.406e-12
variance: 6.085e-19 ± 2.097e-20
skew: 8.709e-01 ± 2.381e-01
kurtosis: 6.194e+00 ± 1.796e+00
Sphere radius
Minimum visibility limit
MC size histogram
[Rel.] Volume Fraction
0.00035
0.0003
0.00025
0.0002
0.00015
0.0001
5e-05
0
Cumulative distribution function
1.2
1
0.8
0.6
0.4
0.2
0
radius (nm)
10
