# Supplementary material for: Nanoparticle size distribution quantification: results of a small-angle X-ray scattering inter-laboratory comparison
Source: J Appl Crystallogr. 2017 Aug 18;50(Pt 5):1280–8. doi: 10.1107/S160057671701010X (PMC5627679; doi:10.1107/S160057671701010X)
Supplement: Supplementary file 1 [file j-50-01280-sup1.zip › QPrecision/data/exDminus0p0925 2016-11-14_15-17-38/exDminus0p0925 2016-11-14_15-17-38.pdf]

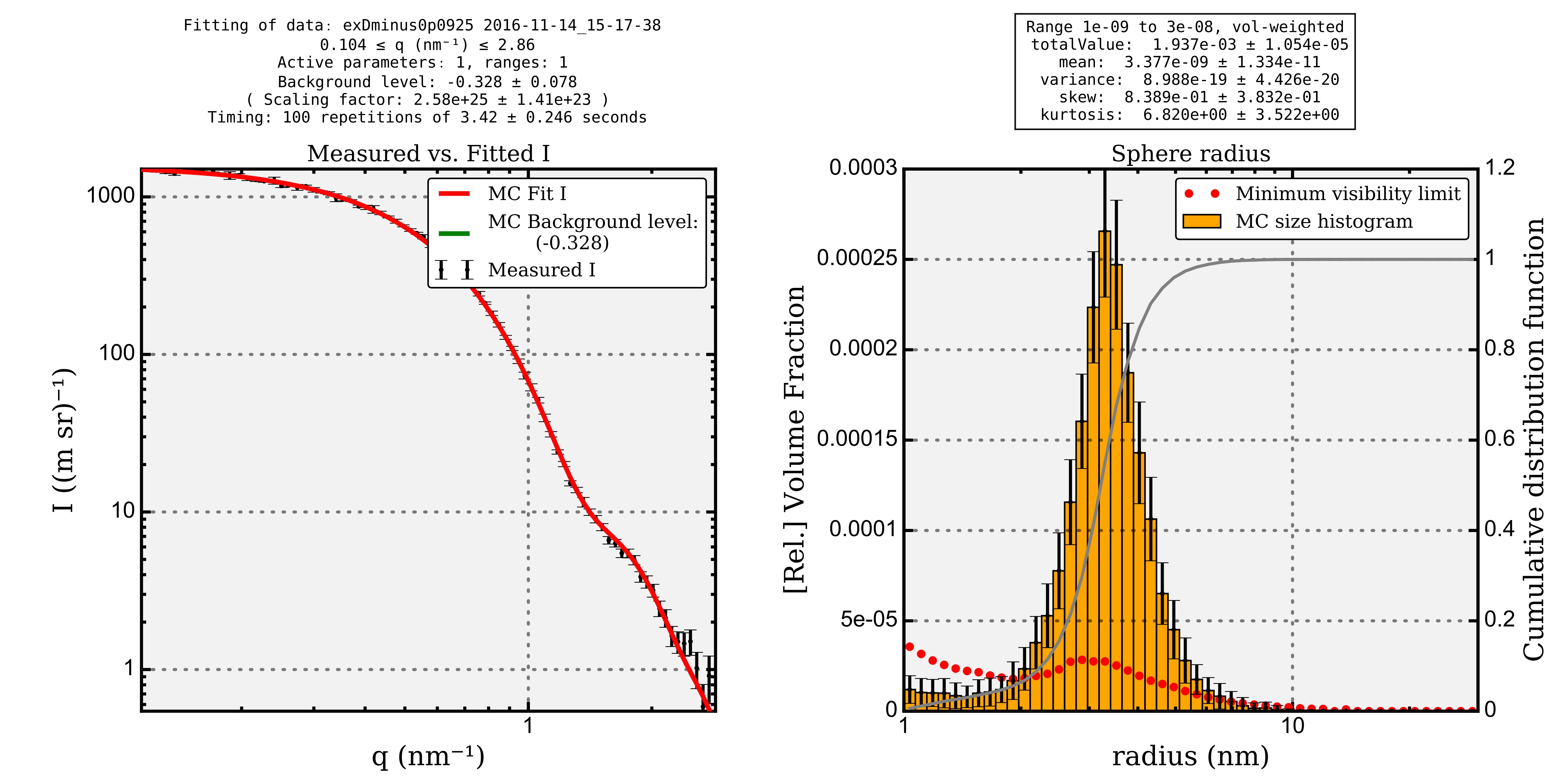
Fitting of data: exDminus0p0925 2016-11-14_15-17-38
0.104 ≤ q (nm⁻¹) ≤ 2.86
Active parameters: 1, ranges: 1
Background level: -0.328 ± 0.078
(Scaling factor: 2.58e+25 ± 1.41e+23)
Timing: 100 repetitions of 3.42 ± 0.246 seconds
Measured vs. Fitted I
MC Fit I
MC Background level: (-0.328)
Measured I
1000
100
10
1
I ((m sr)⁻¹)
q (nm⁻¹)
Range 1e-09 to 3e-08, vol-weighted
totalValue: 1.937e-03 ± 1.054e-05
mean: 3.377e-09 ± 1.334e-11
variance: 8.988e-19 ± 4.426e-20
skew: 8.389e-01 ± 3.832e-01
kurtosis: 6.820e+00 ± 3.522e+00
Sphere radius
Minimum visibility limit
MC size histogram
0.0003
0.00025
0.0002
0.00015
0.0001
5e-05
0
[Rel.] Volume Fraction
1.2
1
0.8
0.6
0.4
0.2
0
Cumulative distribution function
1
10
radius (nm)
