# Supplementary material for: Nanoparticle size distribution quantification: results of a small-angle X-ray scattering inter-laboratory comparison
Source: J Appl Crystallogr. 2017 Aug 18;50(Pt 5):1280–8. doi: 10.1107/S160057671701010X (PMC5627679; doi:10.1107/S160057671701010X)
Supplement: Supplementary file 1 [file j-50-01280-sup1.zip › QPrecision/data/exDplus0p012 2016-11-10_19-21-33/exDplus0p012 2016-11-10_19-21-33.pdf]

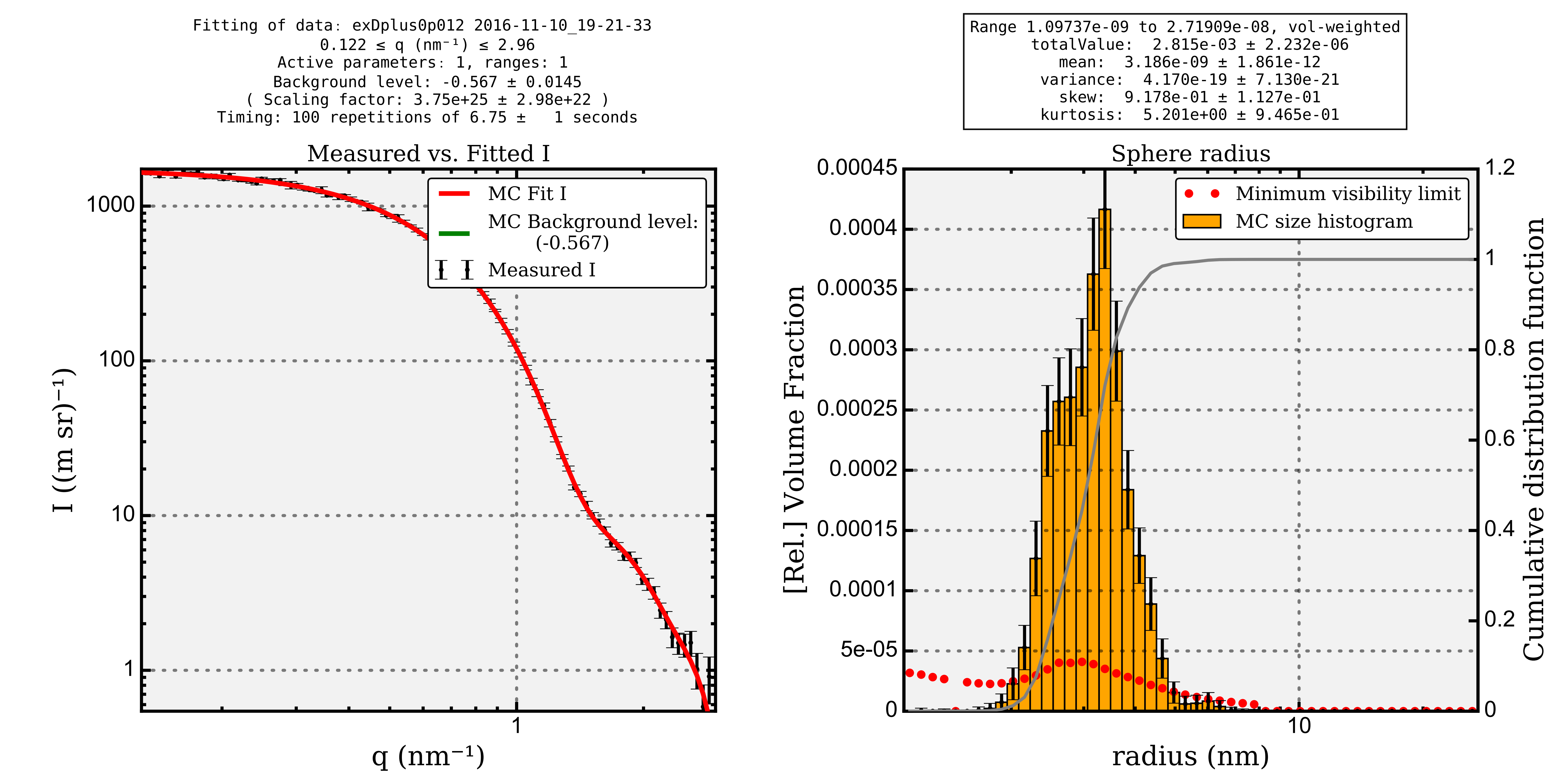
Fitting of data: exDplus0p012 2016-11-10_19-21-33
0.122 ≤ q (nm⁻¹) ≤ 2.96
Active parameters: 1, ranges: 1
Background level: -0.567 ± 0.0145
(Scaling factor: 3.75e+25 ± 2.98e+22)
Timing: 100 repetitions of 6.75 ± 1 seconds
Measured vs. Fitted I
MC Fit I
MC Background level: (-0.567)
Measured I
I ((m sr)⁻¹)
q (nm⁻¹)
1000
100
10
1
Range 1.09737e-09 to 2.71909e-08, vol-weighted
totalValue: 2.815e-03 ± 2.232e-06
mean: 3.186e-09 ± 1.861e-12
variance: 4.170e-19 ± 7.130e-21
skew: 9.178e-01 ± 1.127e-01
kurtosis: 5.201e+00 ± 9.465e-01
Sphere radius
Minimum visibility limit
MC size histogram
[Rel.] Volume Fraction
Cumulative distribution function
radius (nm)
0.00045
0.0004
0.00035
0.0003
0.00025
0.0002
0.00015
0.0001
5e-05
0
1.2
1
0.8
0.6
0.4
0.2
10
