# Supplementary material for: Nanoparticle size distribution quantification: results of a small-angle X-ray scattering inter-laboratory comparison
Source: J Appl Crystallogr. 2017 Aug 18;50(Pt 5):1280–8. doi: 10.1107/S160057671701010X (PMC5627679; doi:10.1107/S160057671701010X)
Supplement: Supplementary file 1 [file j-50-01280-sup1.zip › QPrecision/data/exDplus0p035 2016-11-15_11-24-16/exDplus0p035 2016-11-15_11-24-16.pdf]

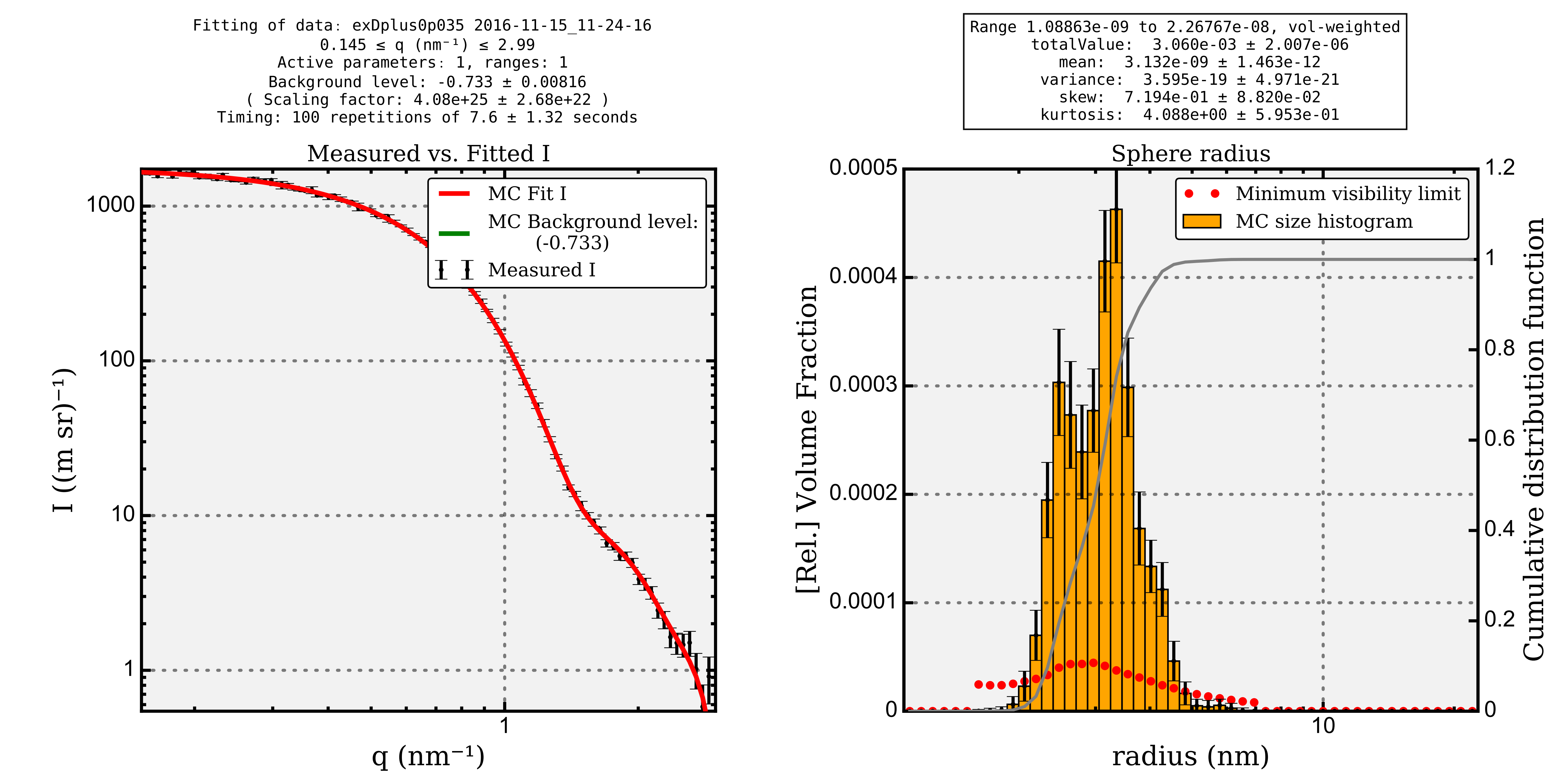

Fitting of data: exDplus0p035 2016-11-15_11-24-16
0.145 ≤ q (nm⁻¹) ≤ 2.99
Active parameters: 1, ranges: 1
Background level: -0.733 ± 0.00816
(Scaling factor: 4.08e+25 ± 2.68e+22)
Timing: 100 repetitions of 7.6 ± 1.32 seconds
Measured vs. Fitted I
MC Fit I
MC Background level: (-0.733)
Measured I
I ((m sr)⁻¹)
1000
100
10
1
q (nm⁻¹)
Range 1.08863e-09 to 2.26767e-08, vol-weighted
totalValue: 3.060e-03 ± 2.007e-06
mean: 3.132e-09 ± 1.463e-12
variance: 3.595e-19 ± 4.971e-21
skew: 7.194e-01 ± 8.820e-02
kurtosis: 4.088e+00 ± 5.953e-01
Sphere radius
Minimum visibility limit
MC size histogram
[Rel.] Volume Fraction
0.0005
0.0004
0.0003
0.0002
0.0001
0
10
radius (nm)
Cumulative distribution function
1.2
1
0.8
0.6
0.4
0.2
0
