# Supplementary material for: Nanoparticle size distribution quantification: results of a small-angle X-ray scattering inter-laboratory comparison
Source: J Appl Crystallogr. 2017 Aug 18;50(Pt 5):1280–8. doi: 10.1107/S160057671701010X (PMC5627679; doi:10.1107/S160057671701010X)
Supplement: Supplementary file 1 [file j-50-01280-sup1.zip › QPrecision/data/exDplus0p0925 2016-11-14_15-27-41/exDplus0p0925 2016-11-14_15-27-41.pdf]

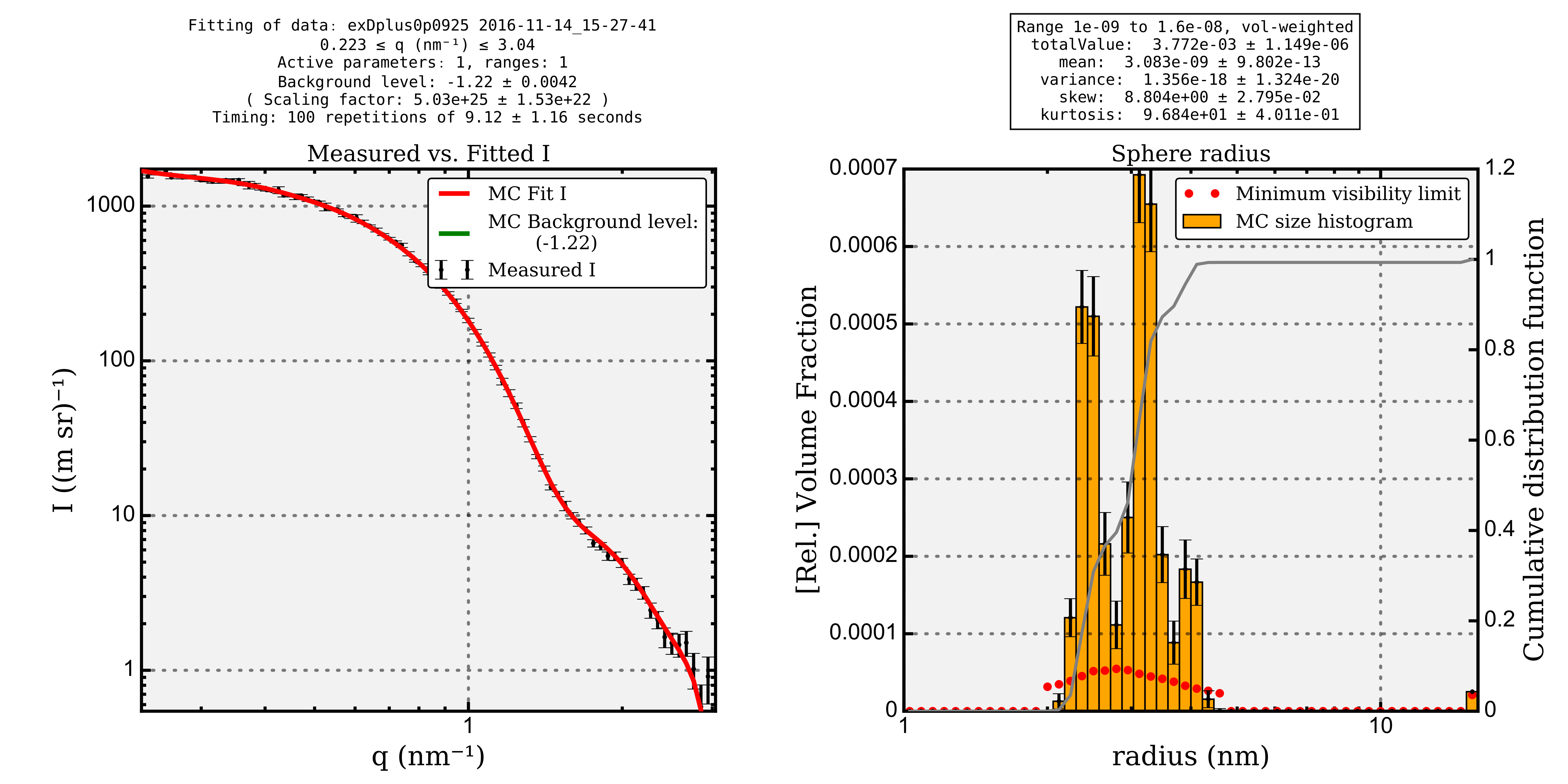

Fitting of data: exDplus0p0925 2016-11-14_15-27-41
0.223 ≤ q (nm⁻¹) ≤ 3.04
Active parameters: 1, ranges: 1
Background level: -1.22 ± 0.0042
(Scaling factor: 5.03e+25 ± 1.53e+22)
Timing: 100 repetitions of 9.12 ± 1.16 seconds
Measured vs. Fitted I
MC Fit I
MC Background level:
(-1.22)
Measured I
I ((m sr)⁻¹)
q (nm⁻¹)
1000
100
10
1
Range 1e-09 to 1.6e-08, vol-weighted
totalValue: 3.772e-03 ± 1.149e-06
mean: 3.083e-09 ± 9.802e-13
variance: 1.356e-18 ± 1.324e-20
skew: 8.804e+00 ± 2.795e-02
kurtosis: 9.684e+01 ± 4.011e-01
Sphere radius
Minimum visibility limit
MC size histogram
[Rel.] Volume Fraction
Cumulative distribution function
radius (nm)
0.0007
0.0006
0.0005
0.0004
0.0003
0.0002
0.0001
0
1.2
1
0.8
0.6
0.4
0.2
10
