# Supplementary material for: Nanoparticle size distribution quantification: results of a small-angle X-ray scattering inter-laboratory comparison
Source: J Appl Crystallogr. 2017 Aug 18;50(Pt 5):1280–8. doi: 10.1107/S160057671701010X (PMC5627679; doi:10.1107/S160057671701010X)
Supplement: Supplementary file 1 [file j-50-01280-sup1.zip › QPrecision/data/exDplus0p0925 2016-11-15_12-04-52/exDplus0p0925 2016-11-15_12-04-52.pdf]

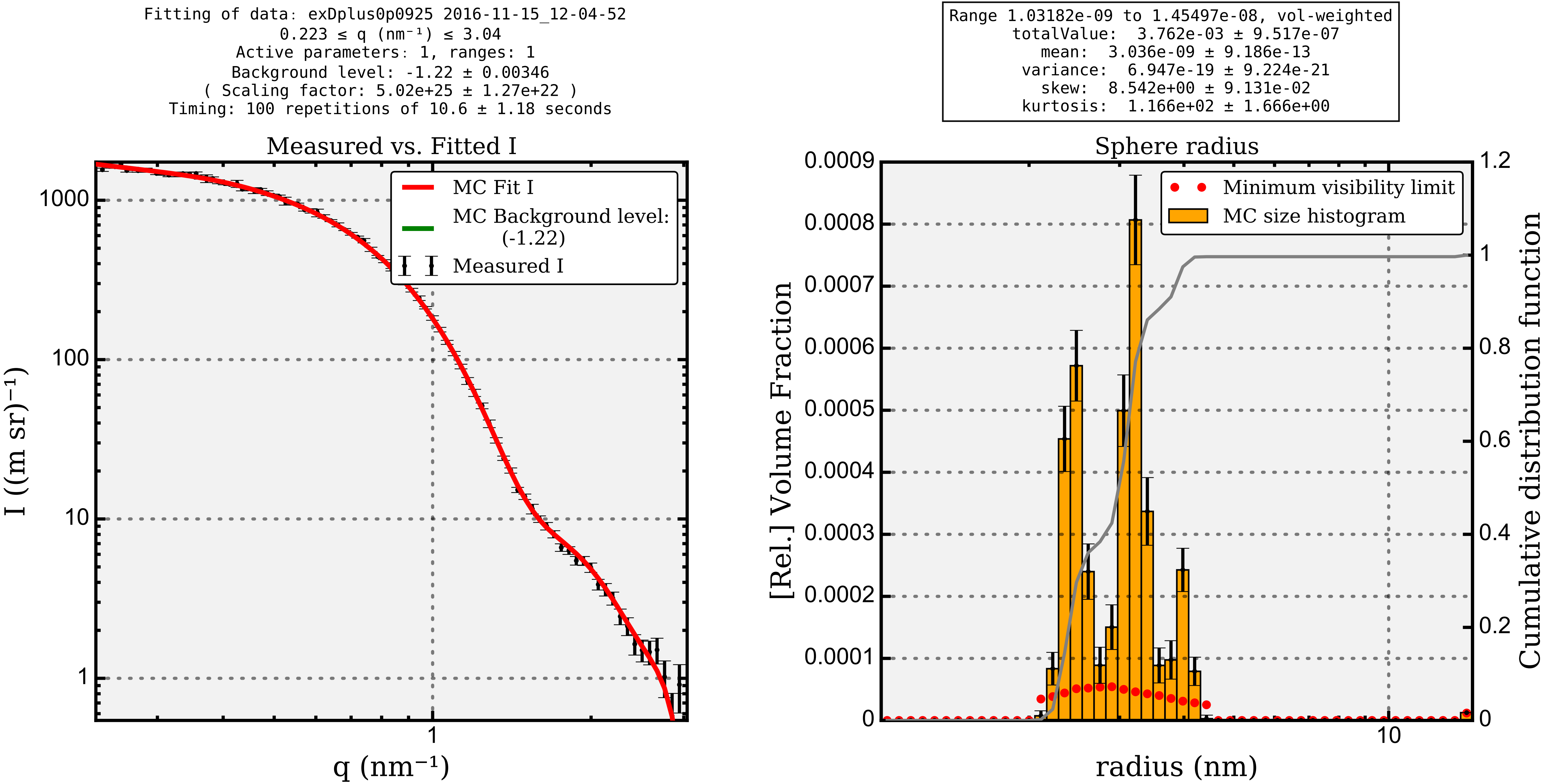
Fitting of data: exDplus0p0925 2016-11-15_12-04-52
0.223 ≤ q (nm⁻¹) ≤ 3.04
Active parameters: 1, ranges: 1
Background level: -1.22 ± 0.00346
(Scaling factor: 5.02e+25 ± 1.27e+22)
Timing: 100 repetitions of 10.6 ± 1.18 seconds
Measured vs. Fitted I
MC Fit I
MC Background level:
(-1.22)
Measured I
I ((m sr)⁻¹)
q (nm⁻¹)
1000
100
10
1
Range 1.03182e-09 to 1.45497e-08, vol-weighted
totalValue: 3.762e-03 ± 9.517e-07
mean: 3.036e-09 ± 9.186e-13
variance: 6.947e-19 ± 9.224e-21
skew: 8.542e+00 ± 9.131e-02
kurtosis: 1.166e+02 ± 1.666e+00
Sphere radius
Minimum visibility limit
MC size histogram
[Rel.] Volume Fraction
Cumulative distribution function
radius (nm)
0.0009
0.0008
0.0007
0.0006
0.0005
0.0004
0.0003
0.0002
0.0001
0
10
1.2
1
0.8
0.6
0.4
0.2
0
