# Supplementary material for: Nanoparticle size distribution quantification: results of a small-angle X-ray scattering inter-laboratory comparison
Source: J Appl Crystallogr. 2017 Aug 18;50(Pt 5):1280–8. doi: 10.1107/S160057671701010X (PMC5627679; doi:10.1107/S160057671701010X)
Supplement: Supplementary file 1 [file j-50-01280-sup1.zip › QPrecision/data/exDtimes0p985 2016-11-10_11-56-40/exDtimes0p985 2016-11-10_11-56-40.pdf]

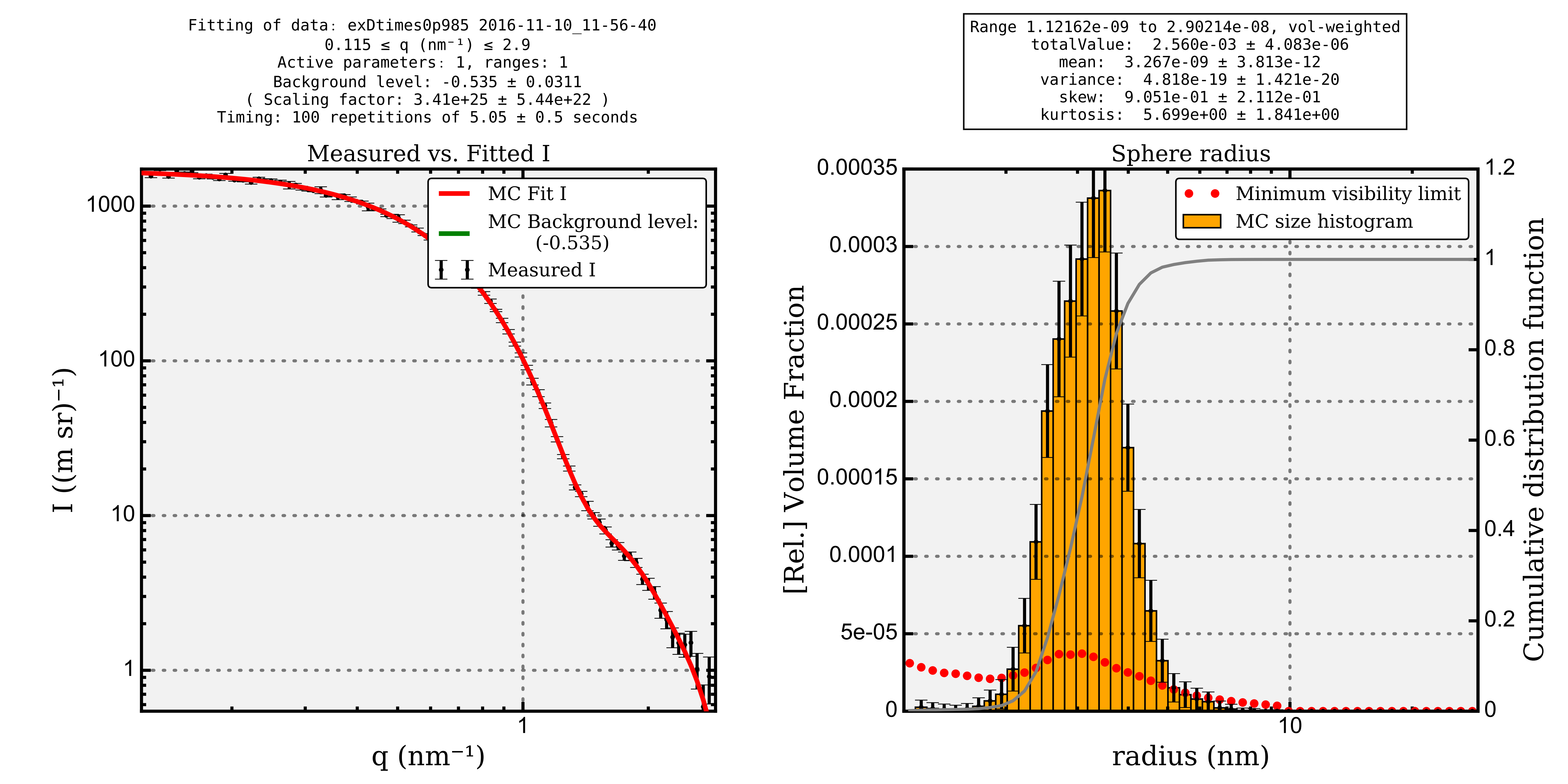

Fitting of data: exDtimes0p985 2016-11-10_11-56-40
0.115 ≤ q (nm⁻¹) ≤ 2.9
Active parameters: 1, ranges: 1
Background level: -0.535 ± 0.0311
(Scaling factor: 3.41e+25 ± 5.44e+22)
Timing: 100 repetitions of 5.05 ± 0.5 seconds
Range 1.12162e-09 to 2.90214e-08, vol-weighted
totalValue: 2.560e-03 ± 4.083e-06
mean: 3.267e-09 ± 3.813e-12
variance: 4.818e-19 ± 1.421e-20
skew: 9.051e-01 ± 2.112e-01
kurtosis: 5.699e+00 ± 1.841e+00
Measured vs. Fitted I
MC Fit I
MC Background level:
(-0.535)
Measured I
I ((m sr)⁻¹)
1000
100
10
1
q (nm⁻¹)
Sphere radius
Minimum visibility limit
MC size histogram
[Rel.] Volume Fraction
0.00035
0.0003
0.00025
0.0002
0.00015
0.0001
5e-05
0
10
radius (nm)
Cumulative distribution function
1.2
1
0.8
0.6
0.4
0.2
0
