# Supplementary material for: Nanoparticle size distribution quantification: results of a small-angle X-ray scattering inter-laboratory comparison
Source: J Appl Crystallogr. 2017 Aug 18;50(Pt 5):1280–8. doi: 10.1107/S160057671701010X (PMC5627679; doi:10.1107/S160057671701010X)
Supplement: Supplementary file 1 [file j-50-01280-sup1.zip › QPrecision/data/exDtimes1p015 2016-11-10_11-42-34/exDtimes1p015 2016-11-10_11-42-34.pdf]

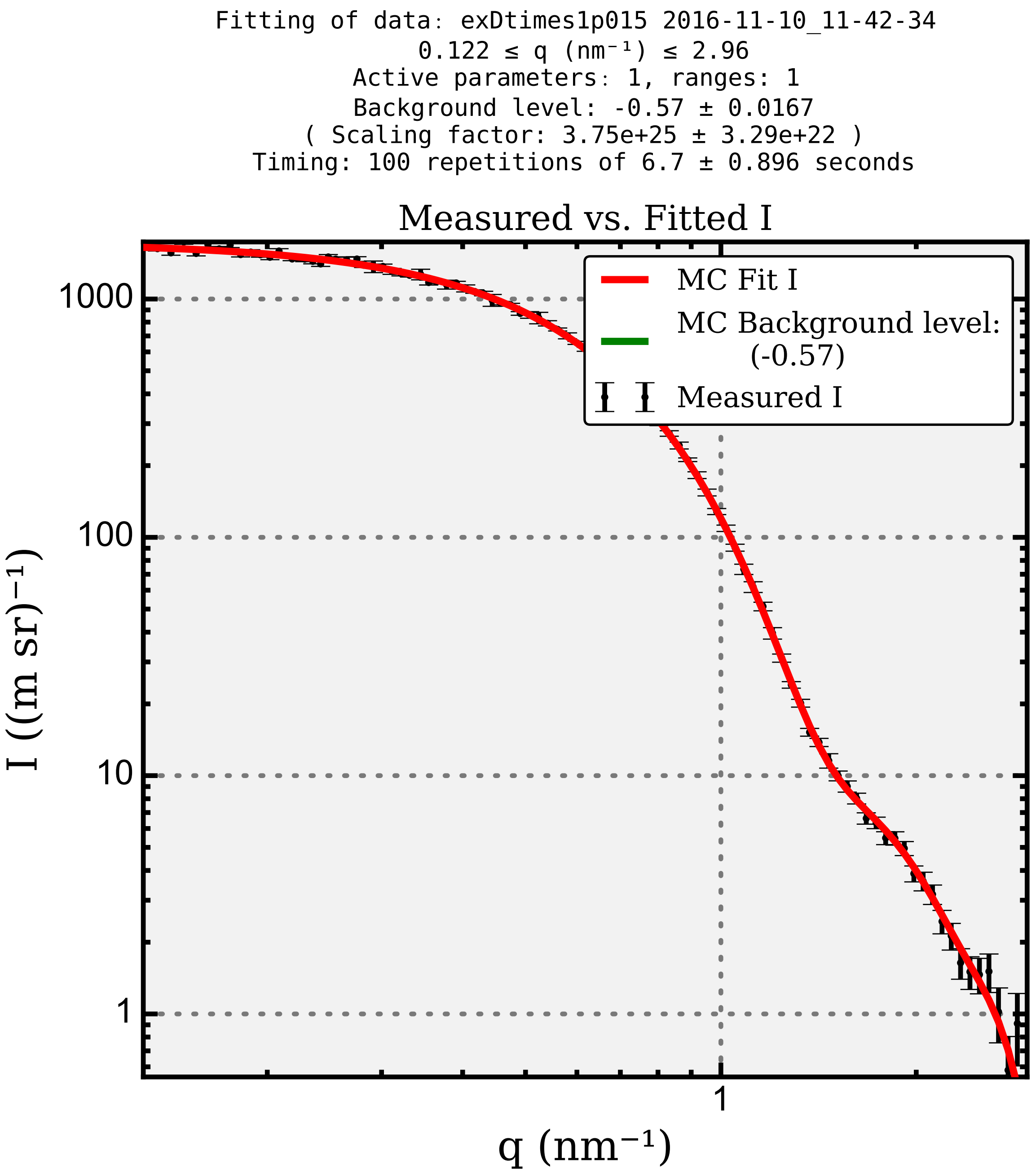
Fitting of data: exDtimes1p015 2016-11-10_11-42-34
0.122 ≤ q (nm⁻¹) ≤ 2.96
Active parameters: 1, ranges: 1
Background level: -0.57 ± 0.0167
(Scaling factor: 3.75e+25 ± 3.29e+22)
Timing: 100 repetitions of 6.7 ± 0.896 seconds
Measured vs. Fitted I
MC Fit I
MC Background level: (-0.57)
Measured I
I ((m sr)⁻¹)
1000
100
10
1
1
q (nm⁻¹)

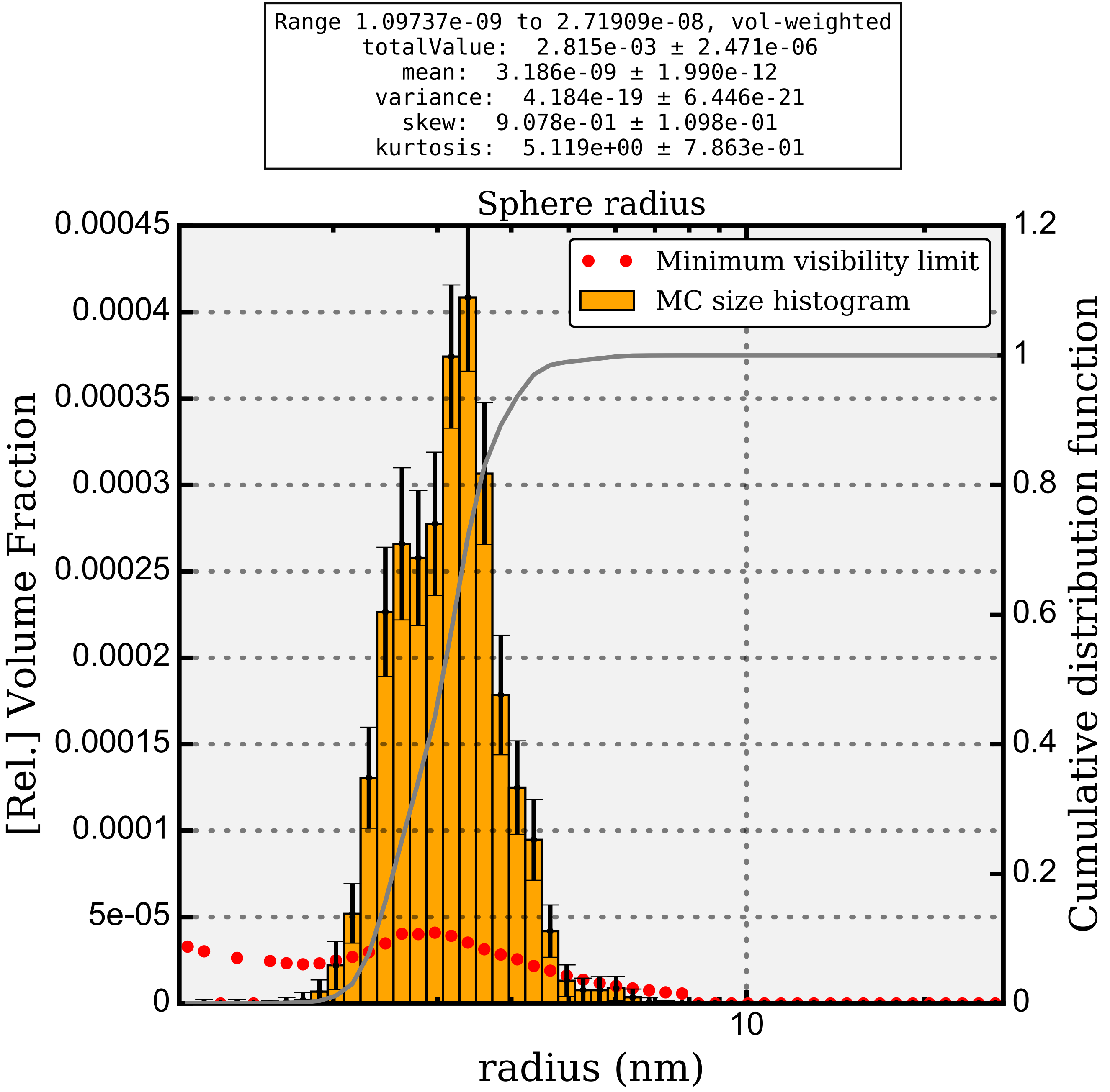
Range 1.09737e-09 to 2.71909e-08, vol-weighted
totalValue: 2.815e-03 ± 2.471e-06
mean: 3.186e-09 ± 1.990e-12
variance: 4.184e-19 ± 6.446e-21
skew: 9.078e-01 ± 1.098e-01
kurtosis: 5.119e+00 ± 7.863e-01
Sphere radius
Minimum visibility limit
MC size histogram
[Rel.] Volume Fraction
0.00045
0.0004
0.00035
0.0003
0.00025
0.0002
0.00015
0.0001
5e-05
0
Cumulative distribution function
1.2
1
0.8
0.6
0.4
0.2
0
10
radius (nm)
