# Supplementary figures and images for: Nanoparticle size distribution quantification: results of a small-angle X-ray scattering inter-laboratory comparison
Source: J Appl Crystallogr. 2017 Aug 18;50(Pt 5):1280–8. doi: 10.1107/S160057671701010X (PMC5627679; doi:10.1107/S160057671701010X)

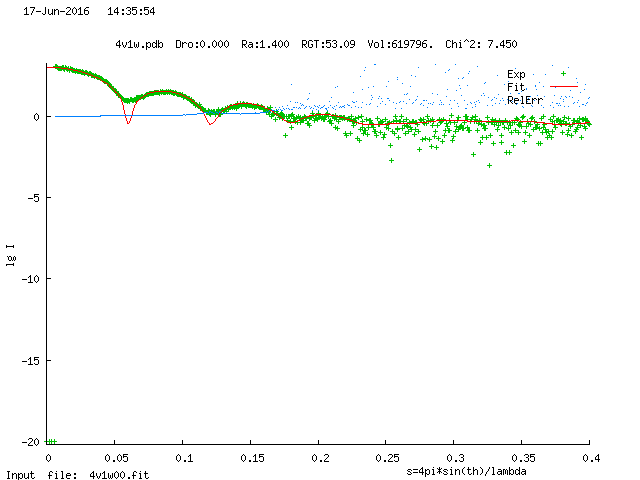

Supplement: Supplementary file 1 [file j-50-01280-sup1.zip › QPrecision/data/Apoferritin/4v1w00.png]

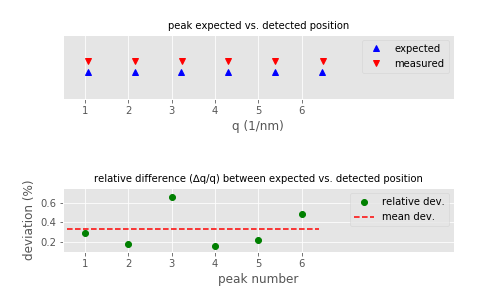

Supplement: Supplementary file 1 [file j-50-01280-sup1.zip › QPrecision/images/AgBehPPos.png]

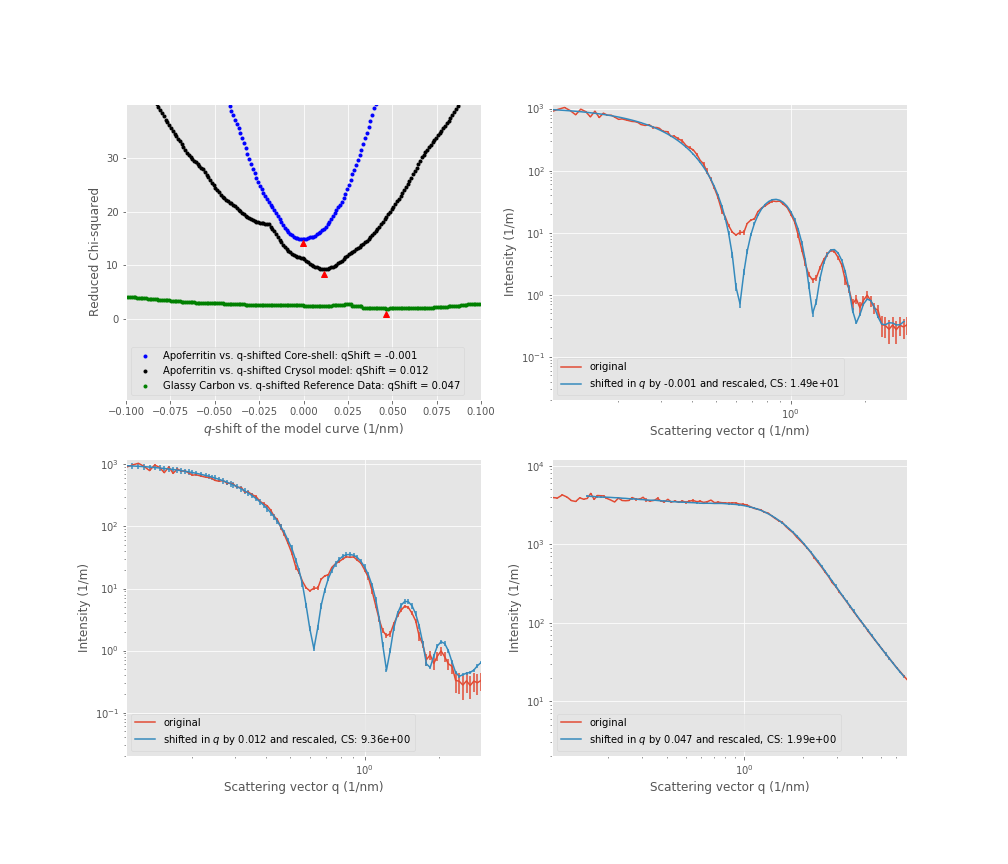

Supplement: Supplementary file 1 [file j-50-01280-sup1.zip › QPrecision/images/AllShift.png]

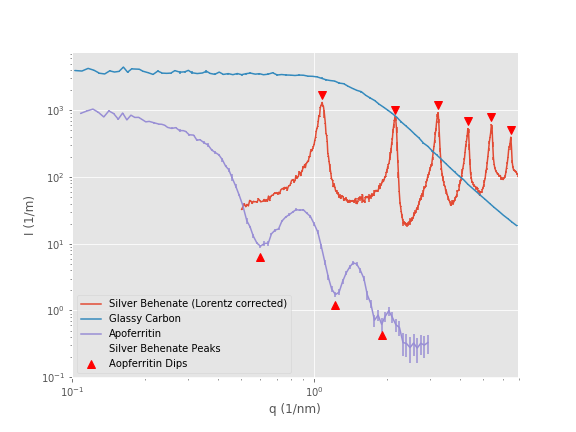

Supplement: Supplementary file 1 [file j-50-01280-sup1.zip › QPrecision/images/Calibrators.png]

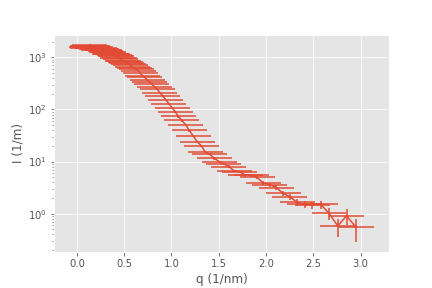

Supplement: Supplementary file 1 [file j-50-01280-sup1.zip › QPrecision/images/QDivShift.png]

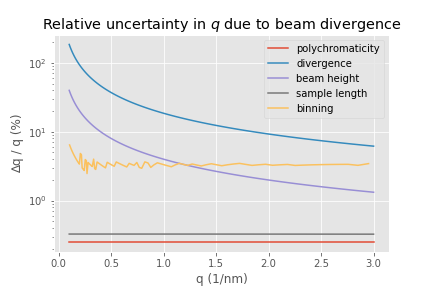

Supplement: Supplementary file 1 [file j-50-01280-sup1.zip › QPrecision/images/QRelContrib.png]

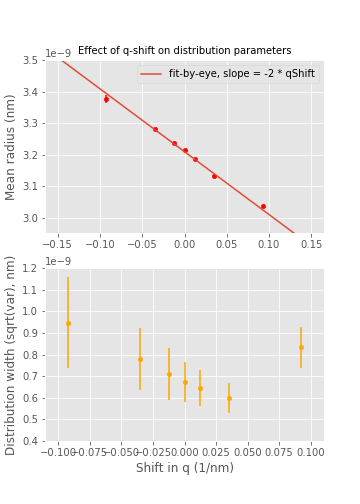

Supplement: Supplementary file 1 [file j-50-01280-sup1.zip › QPrecision/images/QUncEffect.png]

plot of the 28 anonymized datasets on absolute scale  
0.45 % of datapoints are negative and thus omitted.

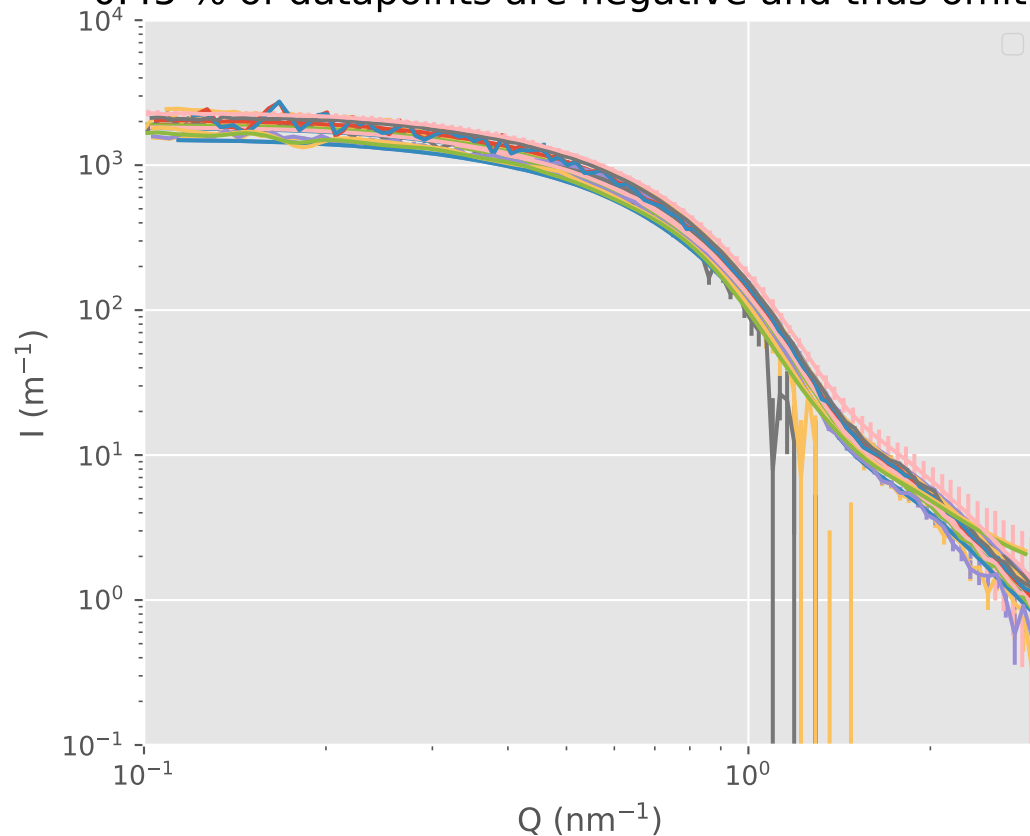

Supplement: Supplementary file 3 [file j-50-01280-sup2.zip › RRAnonData/absoluteCurves.pdf]

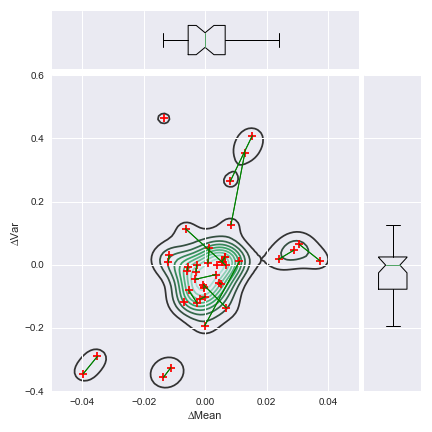

Supplement: Supplementary file 3 [file j-50-01280-sup2.zip › RRAnonData/BivariateKDE.png]

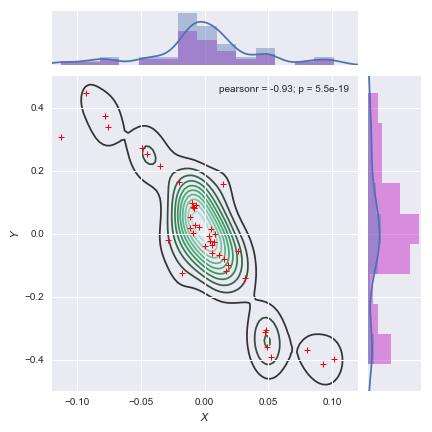

Supplement: Supplementary file 3 [file j-50-01280-sup2.zip › RRAnonData/BivariateKDEn.png]
