# Supplementary material for: Nanoparticle size distribution quantification: results of a small-angle X-ray scattering inter-laboratory comparison
Source: J Appl Crystallogr. 2017 Aug 18;50(Pt 5):1280–8. doi: 10.1107/S160057671701010X (PMC5627679; doi:10.1107/S160057671701010X)
Supplement: Supplementary file 2 [file j-50-01280-sup3.pdf]

JOURNAL OF
APPLIED
CRYSTALLOGRAPHY

Volume 50 (2017)

Supporting information for article:

Nanoparticle size distribution quantification: results of a SAXS [small-angle X-ray scattering] inter-laboratory comparison

Brian R. Pauw, Claudia Kästner and Andreas F. Thünemann

Table of Contents

1. Additional information about samples and fitting values
2. Software Usage Guides (SUGs) for SAXS Data Evaluation Procedures
3. Data evaluation with McSAS
4. An assessment of the calibration of, and uncertainty in q
5. References

[Additional information is also available in two Jupyter notebooks (one describing the q uncertainty calculation, the other dealing with the data input, rebinning and McSAS fit results). It is recommended to open these using the Anaconda python package (https://www.continuum.io/downloads). Unpack the SI-ZIP file and launch the program "Jupyter Notebook" (included in the Anaconda package). With the help of Jupyter Notebook you can open the files QPrecision.ipynb and RoundRobinMcSAS.ipynb.]

1. Additional information about samples and fitting values

We combined the information received from the various participants. We note the following values:

- *Participant:* a number indicating a unique participating laboratory
- *Received Date:* The date on which the measured data was received
- *Sample ID:* The unique ID of the sample vial(s) measured by the laboratory. If a laboratory has measured the sample on a second instrument, 100 is added to the sample vial ID. For example, sample 49 and 149 are samples from the same vial, measured on two instruments. The dataset filenames match this sample ID.
- *Absolute Intensity*: If the data provided by the laboratory has been scaled to absolute units
- *Instrument Type*: "lab" or "synchrotron"
- *Convergence Criterion*: The (McSAS) reduced chi-squared convergence criterion used to define a successful fit. This is 1 when the uncertainties are appropriately estimated, otherwise larger or smaller, depending on the case
- *Outlier*: Marked with an asterisk if the dataset is considered an outlier to the Round Robin study
- *Wavelength*: The wavelength used for the measurement
- *Contrast*: The scattering contrast in reciprocal meters for (bulk) silver in water with that wavelength.

- *Different Capillaries*: Marks whether non-identical capillaries were used for the background and sample measurements.

Table S 1. Information on measurements of the samples. Measurements with absolute intensities, which were used for determination of particle concentrations, are in red colour.

Lab #	Absolute Intensity	Different Capillaries for Sample and Background	Convergence Criterion	Wavelength (m^{-1})	Contrast (m^{-2})	Instrument Type	Received Date (ISO 8601)	Sample ID
0	yes	no	1	1.542e-10	6.8e+15	lab	2016-02-09	[14, 15]
1	yes	no	[1.05, 1.8]	7.108e-11	6.63e+15	lab	2016-02-11	[53, 54]
2*	no	yes	[15.4, 5.4]	NaN	NaN	lab	2016-02-24	[6, 16]
3	no	no	1	NaN	NaN	lab	2016-03-15	[46, 47]
4	yes	no	1	9.9184e-11	6.73e+15	synchrotron	2016-02-18	[10, 11]
5	no	yes	4,3	NaN	NaN	lab	2016-02-17	[28, 32]
6	yes	no	1	1.0332e-10	6.74e+15	synchrotron	2016-02-19	[23, 24]
7	yes	no	1,18	1.542e-10	6.8e+15	lab	2016-02-19	[20, 57]
8	yes	no	4,6	5.90381e-11	6.55e+15	synchrotron	2016-02-22	[62, 63]
9	no	yes	0,1	NaN	NaN	lab	2016-03-03	[43, 45]
10	yes	no	1	1.2398e-10<br>7.74875e-11	6.78e+15<br>6.66e+15	synchrotron	2016-03-11	[36, 37]
11	no	no	1	NaN	NaN	lab	2016-03-16	[30, 31]
12	yes	yes	1,81	1.542e-10	6.8e+15	lab	2016-04-06	[49, 51]

* Identified as an outlier.

13	no	no	1	NaN	NaN	synchrotron	2016-04-06	[149, 151]
14	yes	no	38	1.542e-10	6.8e+15	lab	2016-04-08	[55, 56]
15	no	no	5,5	NaN	NaN	lab	2016-04-08	[155, 156]
16	no	yes	1,2	NaN	NaN	synchrotron	2016-05-07	[17, 18]
17	yes	no	1	9.99839e-11	6.73e+15	synchrotron	2016-04-13	[22, 29]
18	yes	no	1	7.74875e-11	6.66e+15	synchrotron	2016-04-18	[33, 27]
19	yes	yes	6,1	1.72915e-11	6.55e+15	synchrotron	2016-05-13	[34, 35]
20	yes	no	1	1.542e-10	6.8e+15	lab	2016-05-13	[38]
21	yes	no	1,05	1.542e-10	6.8e+15	lab	2016-05-15	[52, 58]
22	no	yes	1,5	NaN	NaN	lab	2016-05-17	[25]
23	yes	no	1	1.542e-10	6.8e+15	lab	2016-05-18	[41]

Table S 2. Summary of the statistical data of the inter-laboratory comparison. The standard error of the mean values of all radii and width values is in the range of 0.01 nm to 0.02 nm.

	IFT				SASfit[a]		McSAS			
Weighting	Number		Volume		Number		Number		Volume	
Statistical value	$R_{n,IFT}$ (nm)	$\sigma_{n,IFT}$ (nm)	$R_{v,IFT}$ (nm)	$\sigma_{v,IFT}$ (nm)	$R_{n,SASfit}$ (nm)	$\sigma_{n,SASfit}$ (nm)	$R_{n,McSAS}$ (nm)	$\sigma_{n,McSAS}$ (nm)	$R_{v,McSAS}$ (nm)	$\sigma_{v,McSAS}$ (nm)
Mean	2.82	0.67	3.22	0.71	2.80	0.60	2.67	0.69	3.18	0.72
Width, SD	0.04	0.02	0.04	0.05	0.07	0.02	0.16	0.08	0.07	0.06
1st quartile Q_1	2.80	0.66	3.20	0.69	2.71	0.59	2.65	0.66	3.17	0.69
Median 2nd quartile Q_2	2.82	0.67	3.21	0.70	2.79	0.60	2.68	0.69	3.18	0.72
3rd quartile Q_3	2.83	0.69	3.23	0.72	2.81	0.60	2.73	0.73	3.20	0.73
interquartile range Q_3-Q_1	0.03	0.03	0.03	0.03	0.03	0.02	0.08	0.07	0.04	0.04
Lower whisker 5%	2.81	0.67	3.20	0.70	2.78	0.59	2.62	0.67	3.16	0.70
Upper whisker 95%	2.83	0.68	3.23	0.73	2.82	0.61	2.72	0.71	3.20	0.74
90% range	0.02	0.01	0.03	0.03	0.04	0.02	0.10	0.04	0.04	0.04

[a]The SASfit software provides estimates of the number weighted particle properties.

Table S 3. Summary of concentration determination of the statistical data of the inter-laboratory comparison with SASfit and McSAS (The current implementation of IFT does not provide an estimate of the concentration).

	SASfit		McSAS	
Weighting	Number	Volume[a]	Number[b]	Volume
Statistical value	$c_{n,SASfit}$ (10^{-6} mol L^{-1})	$c_{v,SASfit}$ (g L^{-1})	$c_{n,McSAS}$ (10^{-6} mol L^{-1})	$c_{v,McSAS}$ (g L^{-1})
Mean	4.20	3.00	3.37	2.86
Width, SD	0.73	0.38	0.37	0.31
1st quartile Q_1	3.55	2.70	3.16	2.69
Median 2nd quartile Q_2	4.28	3.03	3.40	2.89
3rd quartile Q_3	4.63	3.21	3.57	3.04
interquartile range Q_3-Q_1	1.08	1.56	0.41	0.35
Lower whisker 5%	3.91	2.85	3.23	2.75
Upper whisker 95%	4.48	3.14	3.51	2.99
5%-95% range	0.43	0.29	0.28	0.24

[a]The SASfit software provides estimates of the number weighted particle properties. Volume-weighted concentrations were calculated by conversion of number-weighted values. [b]The McSAS software provides volume-weighted concentrations. Number-weighted concentration was calculated by conversion of volume-weighted values from McSAS.

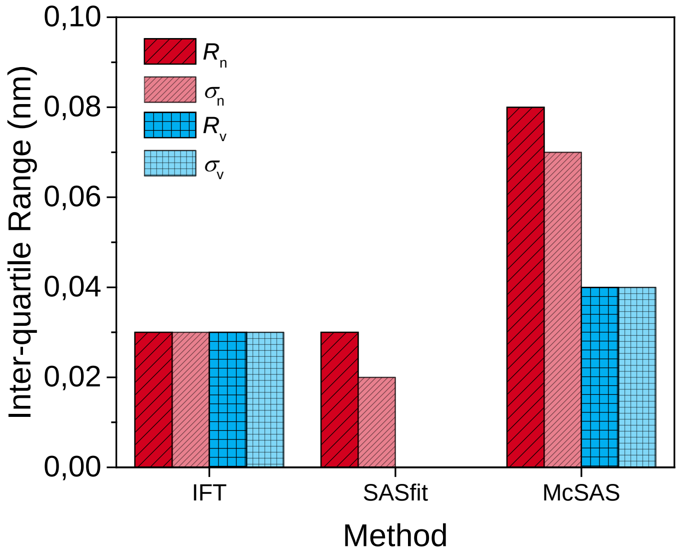

Figure S 1. Inter-quartile ranges of number- and volume-weighted radii and distribution widths derived from IFT, SASfit and McSAS method. The number-weighted values are in given in red, volume-weighted values are in blue color. SASfit provides no volume-weighted values.

Scattering intensities

During the re-binning/anonymization procedure (see main text), a coarse scaling factor was applied to the datasets not on absolute scale, to bring them approximately in line with those on absolute scale.

Minimal variation

For the evaluation of the scaling-independent similarity in scattering profile (main text, Figure 1), we scaled each dataset to best match with a reference dataset. In this case, we use the dataset from sample number 22 as a reference, which is a low-noise dataset. We determine the scaling factor using a least-squares procedure, weighting by the data point uncertainties of both datasets (reference as well as the to-scale dataset). We do not add a flat background to this fitting

procedure as we do not want to modify the datasets. This procedure is available in the RoundRobinMcSAS Jupyter Notebook.

Since the datasets are not necessarily provided with data points at the same q-values, the fitting procedure uses linear interpolation for intensity and uncertainty values between neighboring data points to get a matching set.

2. SUGs for SAXS Data Evaluation Procedures

Indirect Fourier Transformation (IFT)

(a) File specifications

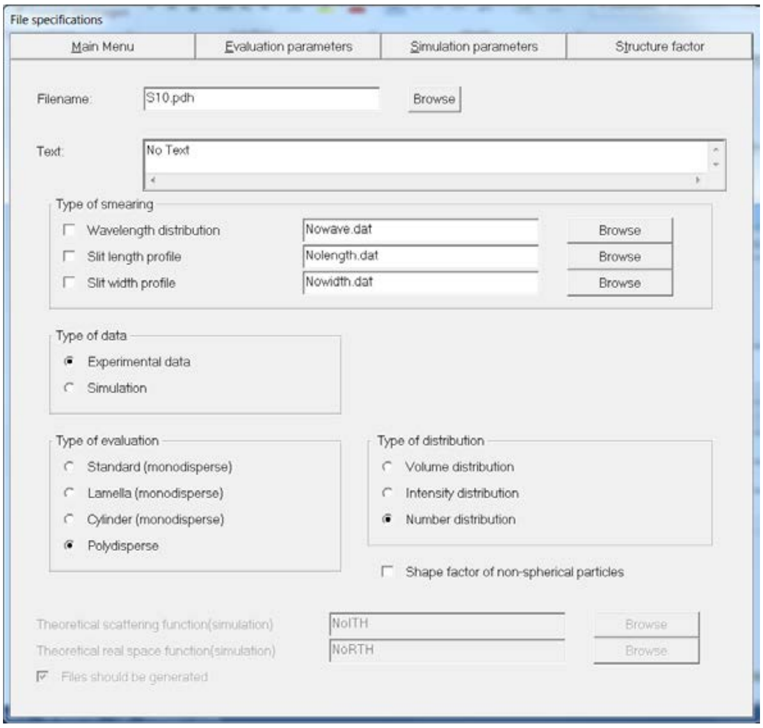

(b) Evaluation parameter

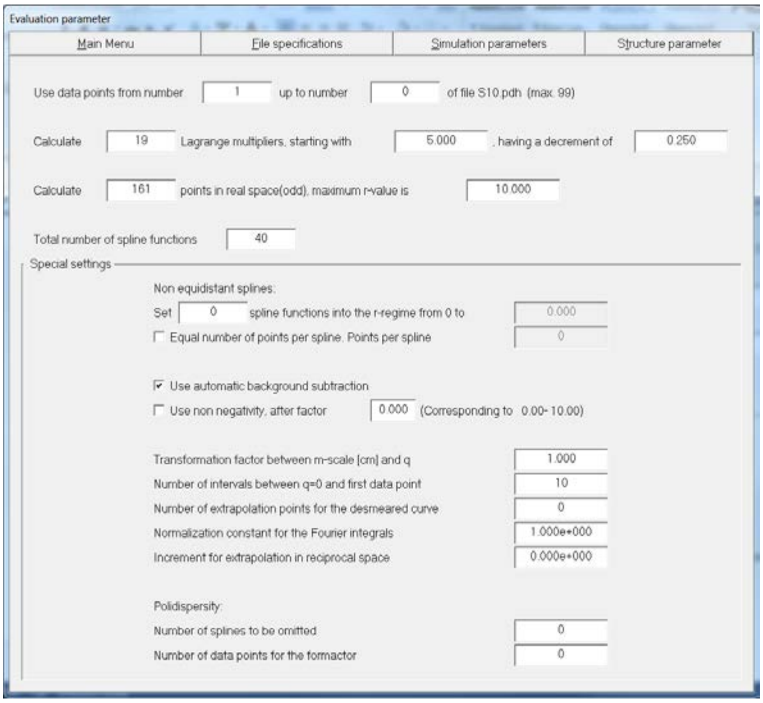

(c) Main menu: Start calculation

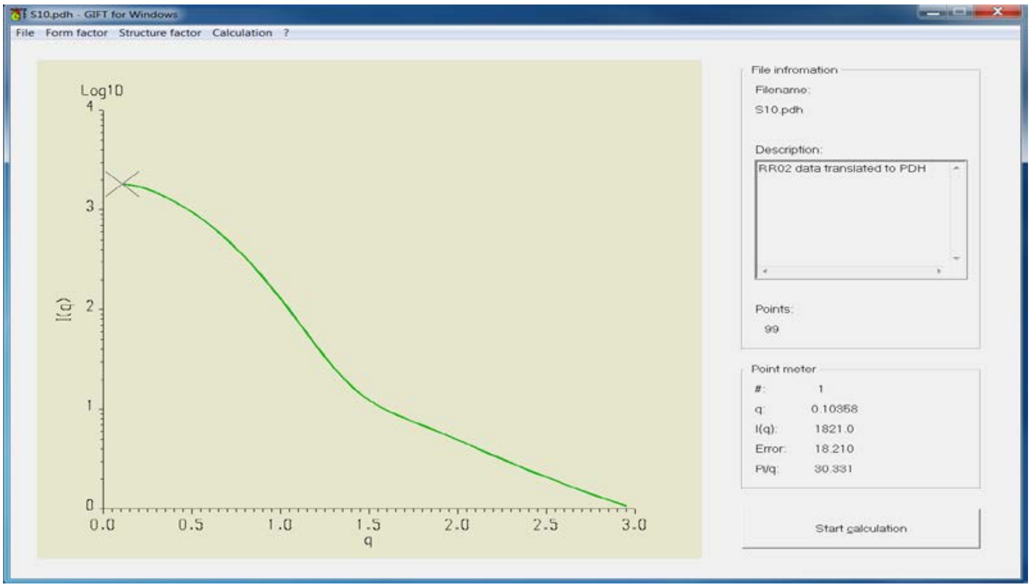

(d) Main menu: result of calculation

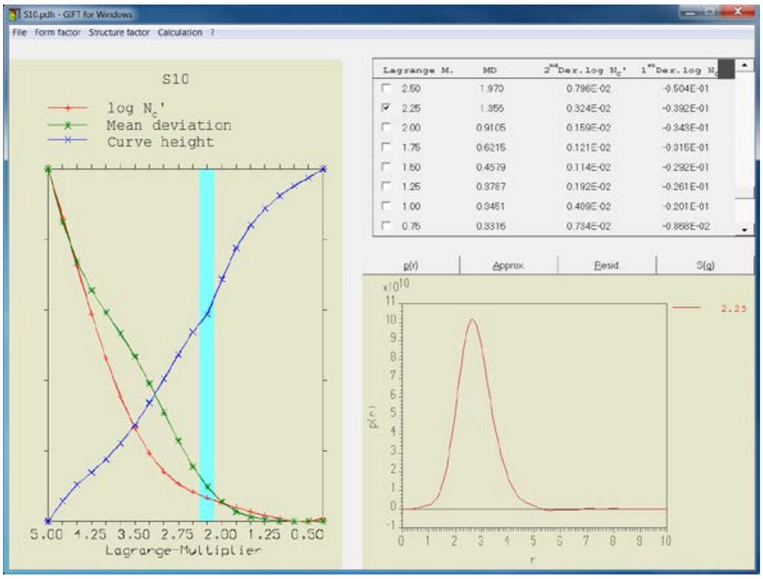

(d1)

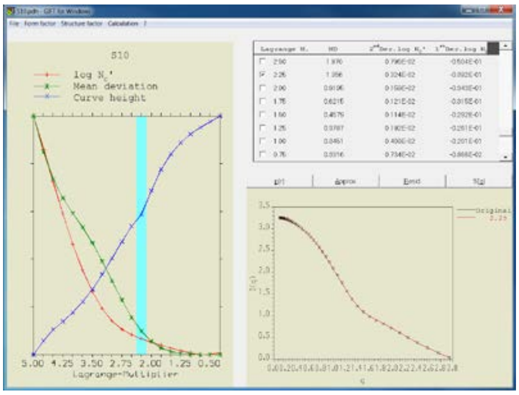

(d2)

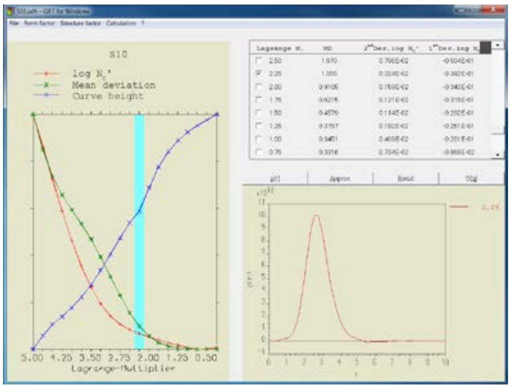

Figure S 2. Software usage guide (SUG) for the SAXS data evaluation when applying the IFT method. This SUG is carried out with the S10 data file for determination of number-weighted size distribution following the following consecutive steps: 1.) Start the evaluation program GIFT, 2.) Load in the data file S10 and click the parameter boxes according to panel (a) (We recommend to use short file paths without special characters when running GIFT for undisturbed data evaluation). 3.) Selection of evaluation parameters in panel (b). 4.) Selection of main menu and start of calculation as shown in panel (c). 5.) Testing of different Lagrange parameters and picking of the most appropriate, which is 2.25 in this example. Several criteria must be fulfilled. First, the experimental curve must be well fitted (panel d1), Second, the distribution must not display strong oscillation with regions of large negative values (panel d2). In this case we were "satisfied" with a Lagrange parameter of 2.25. 6.) Saving the solution under the button "File" and "Save solution as" "S010.por.

The same procedure has to be repeated for extraction of the volume-weighted size distribution (click volume-weighting in step 2, panel a): Note that the range of Lagrange parameters for choosing the optimum parameters has to be adapted properly.

McSAS (Monte Carlo approach for SAS data)

The SUG employed for fitting the datasets using McSAS. Each dataset is loaded into the program using the right-click menu. The size-range to fit over is transferred to the model settings through a double-click on the filename row. Subsequently, the algorithm settings are set to use an appropriate convergence criterion (which is 1 by default, but is modified for a few measurements whose uncertainty estimates were obviously over- or under-estimated). 100 repetitions are chosen of 300 contributions each for the final fit, in order to get a reduced uncertainty on the higher modes of the resulting distributions. The only remaining adaptation is the adjustment of the scattering length density, which is energy dependent. Pressing "start" will let the algorithm run, and the resulting distribution parameters can be extracted from the files written upon successful completion of the optimizations.

(a)

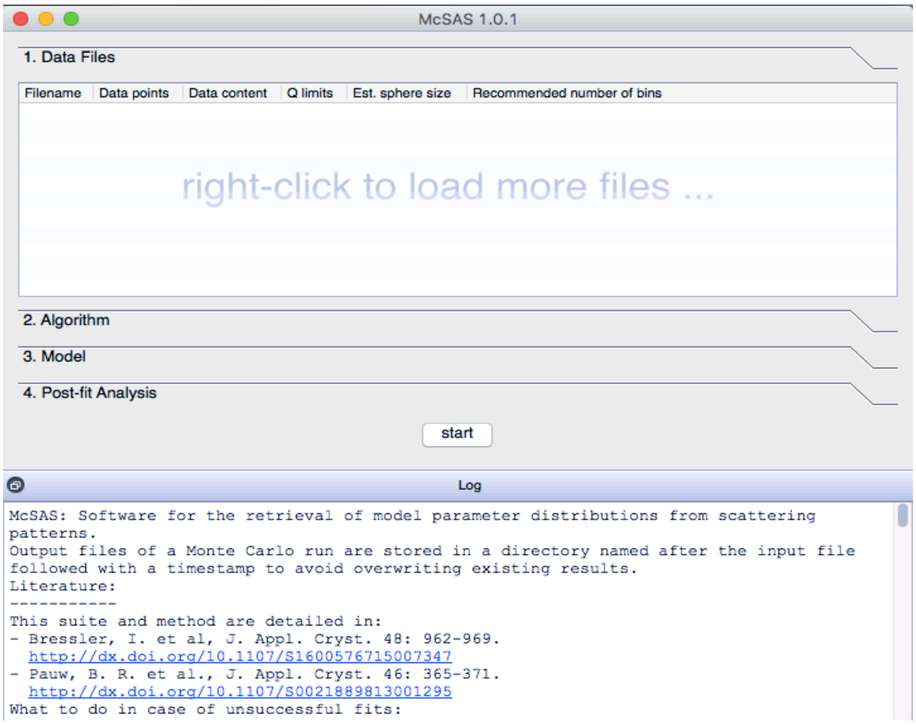

(b)

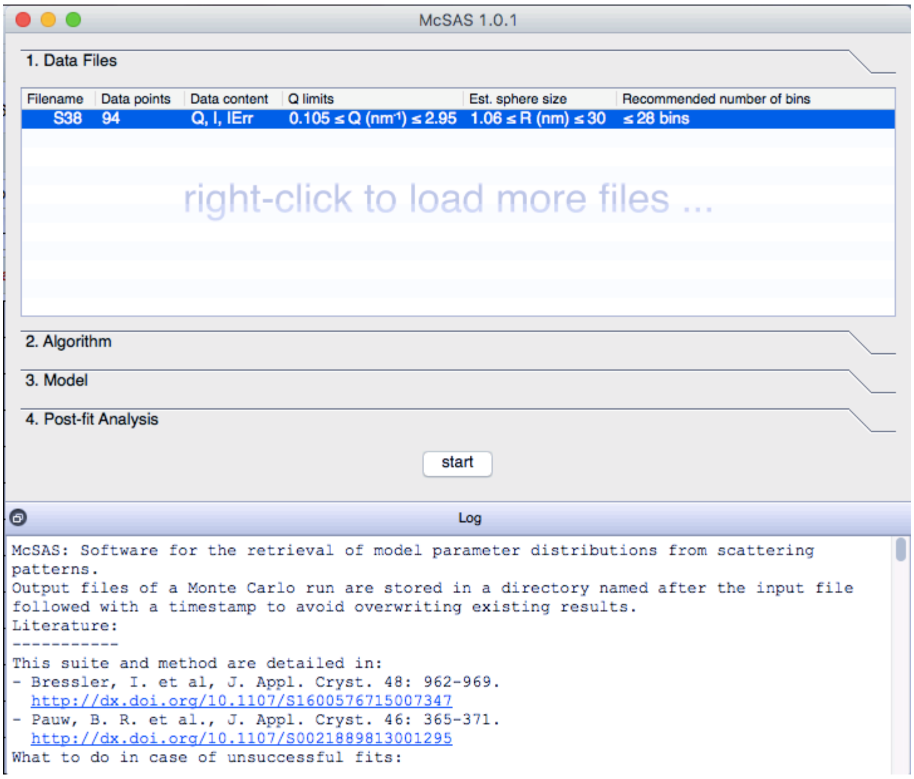
McSAS 1.0.1
1. Data Files
Filename
Data points
Data content
Q limits
Est. sphere size
Recommended number of bins
S38
94
Q, I, IErr
0.105 ≤ Q (nm⁻¹) ≤ 2.95
1.06 ≤ R (nm) ≤ 30
≤ 28 bins
right-click to load more files ...
2. Algorithm
3. Model
4. Post-fit Analysis
start
Log
McSAS: Software for the retrieval of model parameter distributions from scattering patterns.
Output files of a Monte Carlo run are stored in a directory named after the input file followed with a timestamp to avoid overwriting existing results.
Literature:

This suite and method are detailed in:
- Bressler, I. et al, J. Appl. Cryst. 48: 962-969.
http://dx.doi.org/10.1107/S1600576715007347
- Pauw, B. R. et al., J. Appl. Cryst. 46: 365-371.
http://dx.doi.org/10.1107/S0021889813001295
What to do in case of unsuccessful fits:

(c)

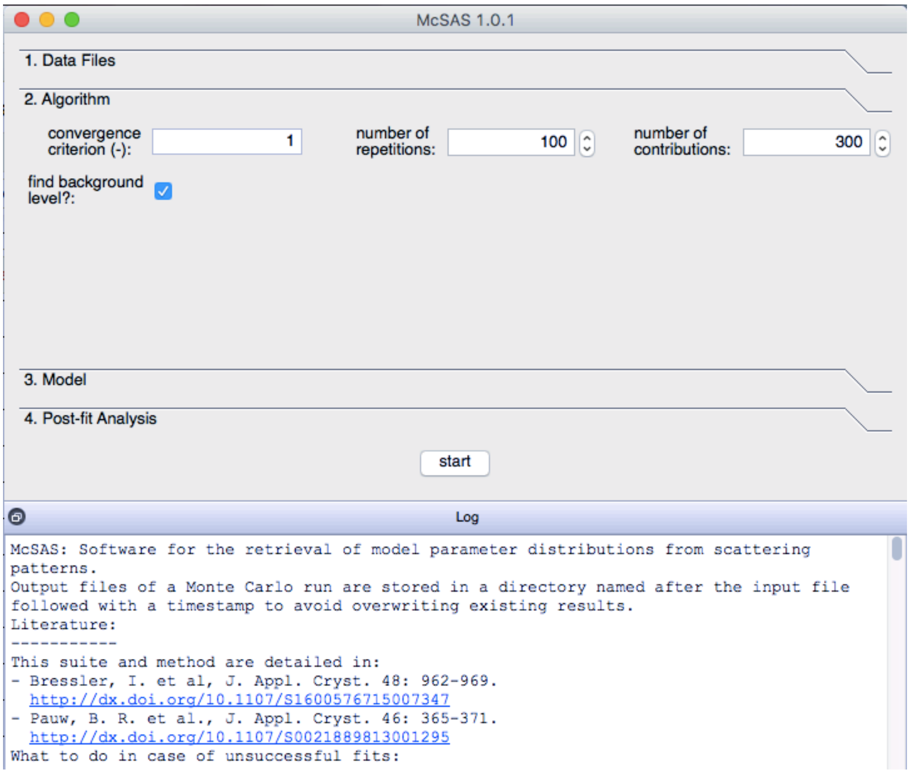
McSAS 1.0.1
1. Data Files
2. Algorithm
convergence criterion (-):
1
number of repetitions:
100
number of contributions:
300
find background level?:
3. Model
4. Post-fit Analysis
start
Log
McSAS: Software for the retrieval of model parameter distributions from scattering patterns.
Output files of a Monte Carlo run are stored in a directory named after the input file followed with a timestamp to avoid overwriting existing results.
Literature:

This suite and method are detailed in:
- Bressler, I. et al, J. Appl. Cryst. 48: 962-969.
http://dx.doi.org/10.1107/S1600576715007347
- Pauw, B. R. et al., J. Appl. Cryst. 46: 365-371.
http://dx.doi.org/10.1107/S0021889813001295
What to do in case of unsuccessful fits:

(d)

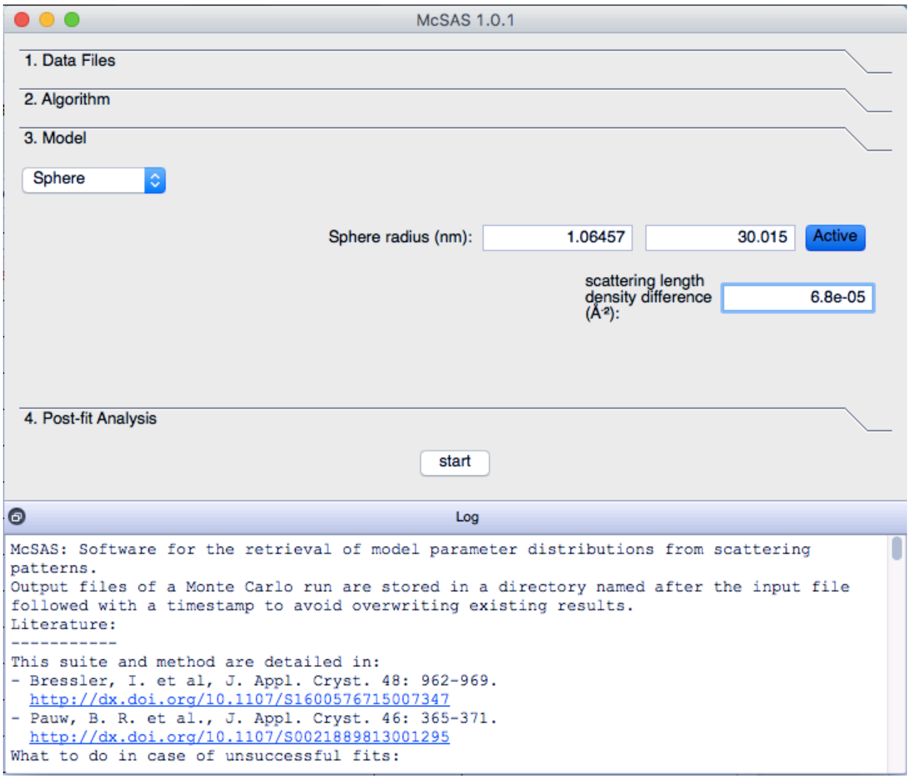
McSAS 1.0.1
1. Data Files
2. Algorithm
3. Model
Sphere
Sphere radius (nm):
1.06457
30.015
Active
scattering length density difference (Å⁻²):
6.8e-05
4. Post-fit Analysis
start
Log
McSAS: Software for the retrieval of model parameter distributions from scattering patterns.
Output files of a Monte Carlo run are stored in a directory named after the input file followed with a timestamp to avoid overwriting existing results.
Literature:

This suite and method are detailed in:
- Bressler, I. et al, J. Appl. Cryst. 48: 962-969.
http://dx.doi.org/10.1107/S1600576715007347
- Pauw, B. R. et al., J. Appl. Cryst. 46: 365-371.
http://dx.doi.org/10.1107/S0021889813001295
What to do in case of unsuccessful fits:

(e)

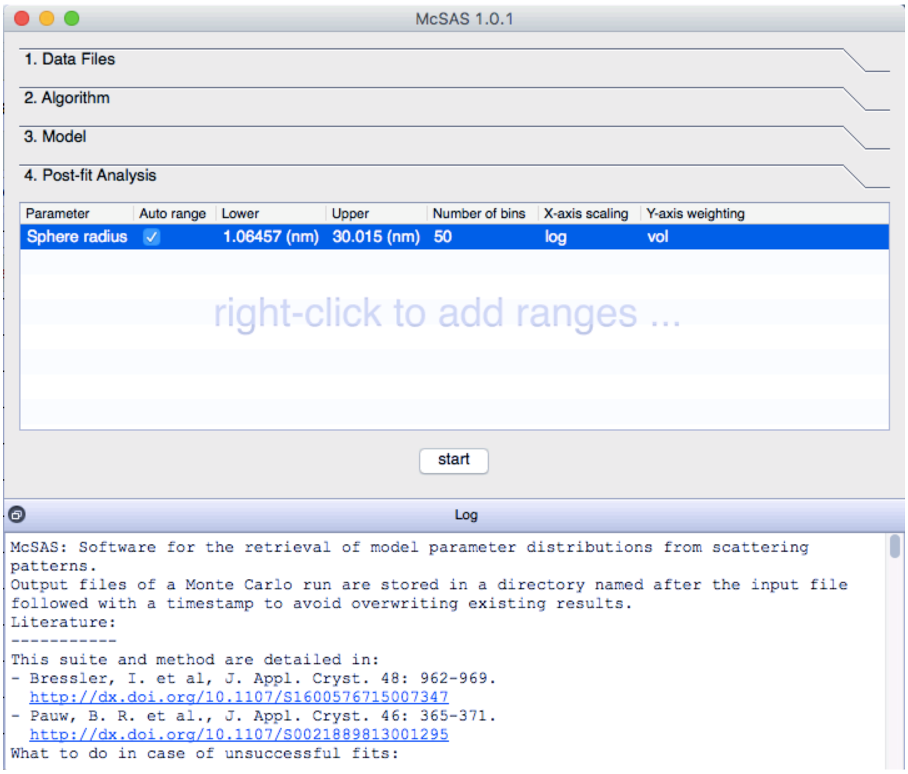
McSAS 1.0.1
1. Data Files
2. Algorithm
3. Model
4. Post-fit Analysis
Parameter
Auto range
Lower
Upper
Number of bins
X-axis scaling
Y-axis weighting
Sphere radius
1.06457 (nm)
30.015 (nm)
50
log
vol
right-click to add ranges ...
start
Log
McSAS: Software for the retrieval of model parameter distributions from scattering patterns.
Output files of a Monte Carlo run are stored in a directory named after the input file followed with a timestamp to avoid overwriting existing results.
Literature:

This suite and method are detailed in:
- Bressler, I. et al, J. Appl. Cryst. 48: 962-969.
http://dx.doi.org/10.1107/S1600576715007347
- Pauw, B. R. et al., J. Appl. Cryst. 46: 365-371.
http://dx.doi.org/10.1107/S0021889813001295
What to do in case of unsuccessful fits:

(f)

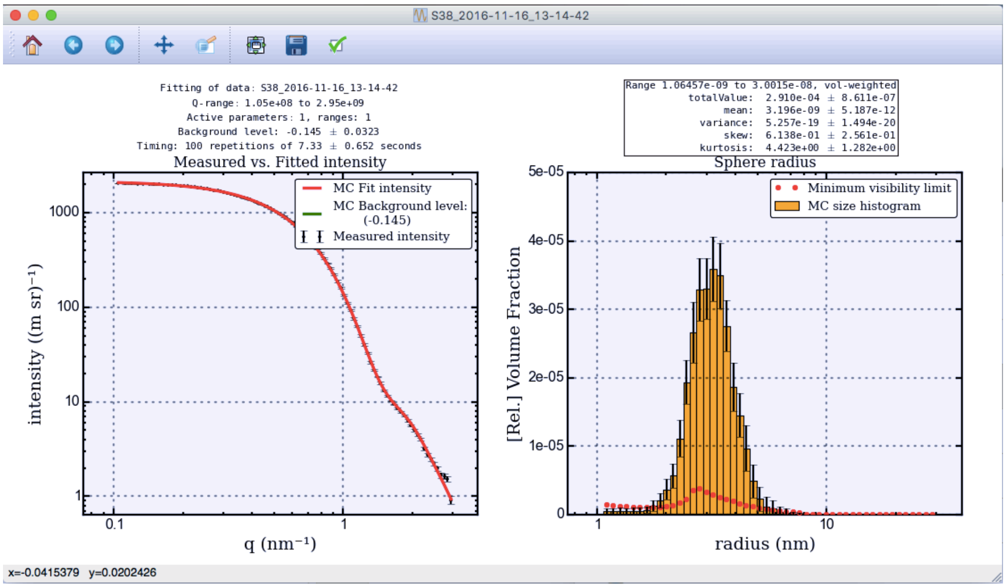

Figure S 3. SUG for the SAXS data evaluation when applying the McSAS method. (a) McSAS default window, in which the datafiles can be selected and loaded. (b) One datafile has been loaded into the program. A double-click on the highlighted row will transfer the estimated sphere sizes to the model radius input. (c) The settings for the optimization algorithm. Most files can be fit using a convergence criterion of 1, except for those with over- or under-estimated data point uncertainties. (d) The model settings (with automatically transferred radii sizes). The scattering length density has to be entered manually, and is wavelength dependent. (e) The post-fit analysis settings, which have not been modified from the default. (f) The result window after 100 optimization steps.

<u>SASfit (classical curve fitting)</u>

The evaluation procedure with SASfit is described in detail in the publication of Breßler et al.(Bressler *et al.*, 2015). A step-by-step example of using SASfit is given below.

(a)

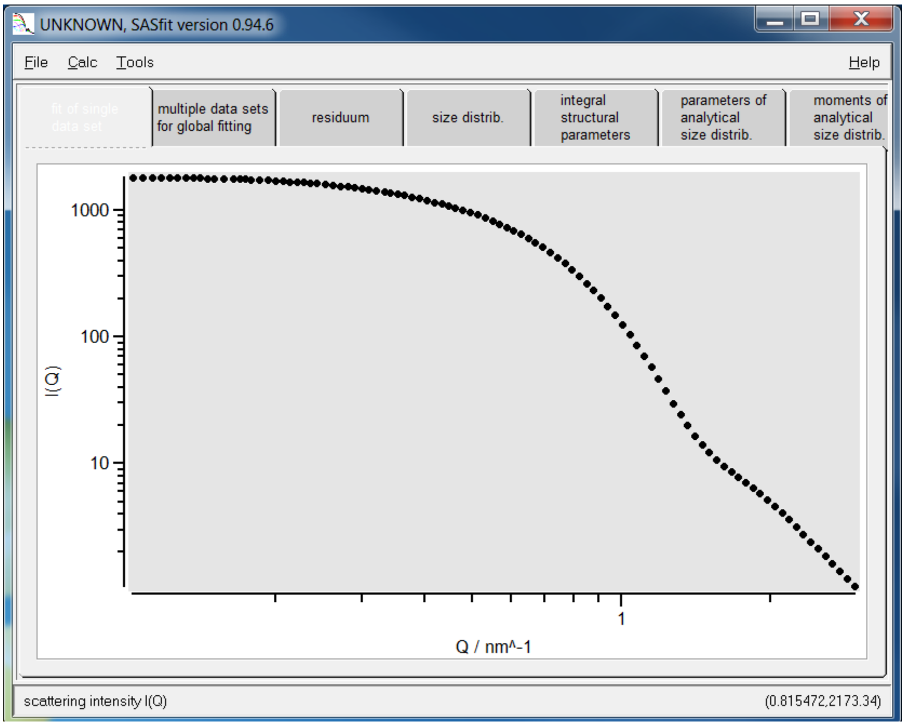
UNKNOWN, SASfit version 0.94.6
File
Calc
Tools
Help
multiple data sets for global fitting
residuum
size distrib.
integral structural parameters
parameters of analytical size distrib.
moments of analytical size distrib.
1000
100
10
I(Q)
1
Q / nm^-1
scattering intensity I(Q)
(0.815472,2173.34)

(b)

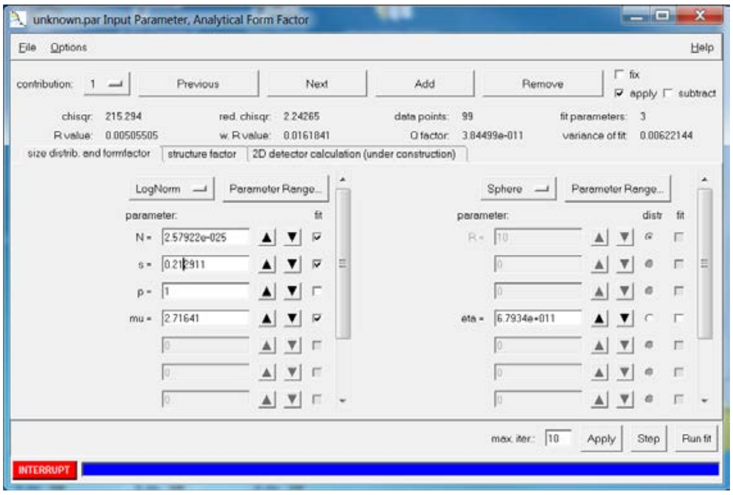
unknown.par Input Parameter, Analytical Form Factor
File
Options
Help
contribution: 1
Previous
Next
Add
Remove
fix
apply
subtract
chisqr: 215.294
red. chisqr: 2.24265
data points: 99
fit parameters: 3
R value: 0.00505505
w. R value: 0.0161841
Q factor: 3.84499e-011
variance of fit: 0.00622144
size distrib. and formfactor
structure factor
2D detector calculation (under construction)
LogNorm
Parameter Range...
Sphere
Parameter Range...
parameter:
fit
distr
N = 2.57922e-025
s = 0.212911
p = 1
mu = 2.71641
R = 10
eta = 6.7934e+011
max. iter.: 10
Apply
Step
Run fit
INTERRUPT

(c)

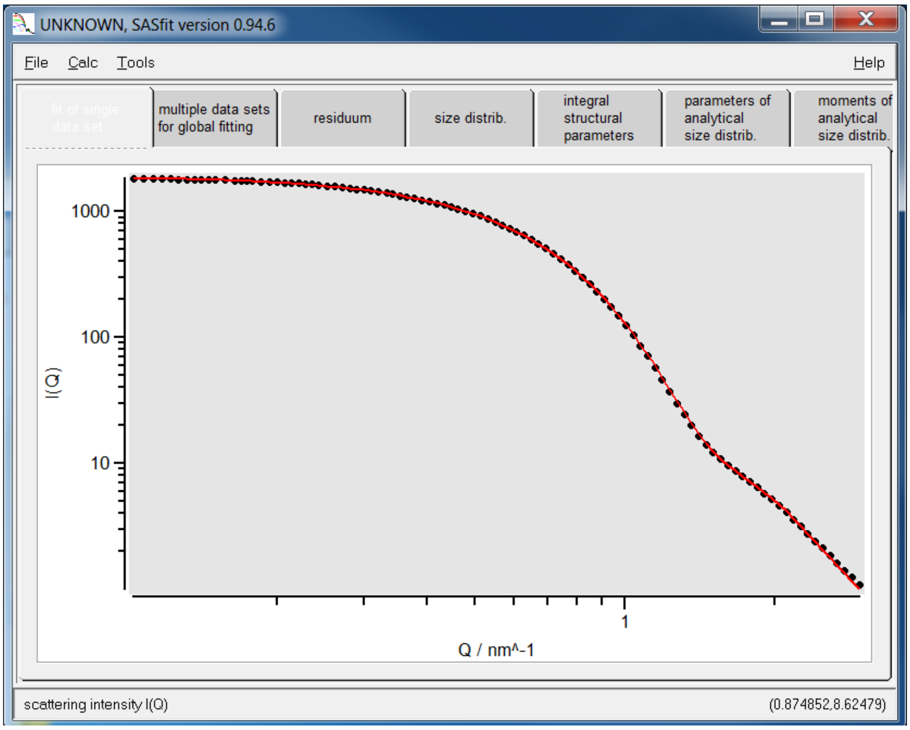

(d)

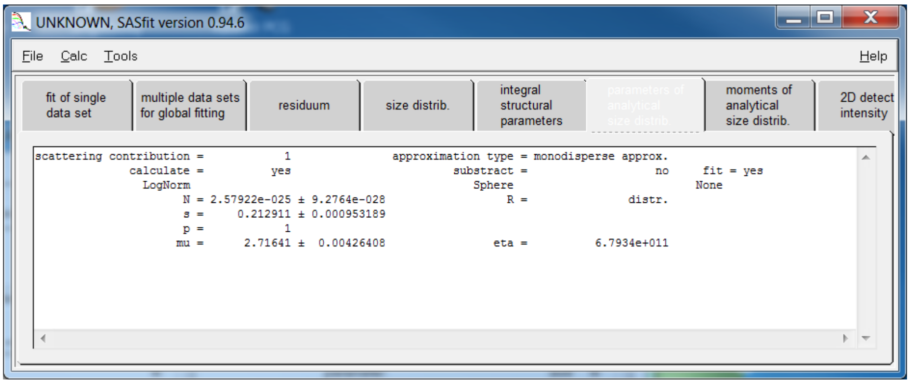

Figure S 4. SUG for the SAXS data evaluation when applying curve fitting using SASfit with log-normal particle size distribution. (a) SASfit default window, in which the datafile can be selected and loaded (click the File button, click the Single Data Set button, browse and insert the data file, e.g. S10). (b) One datafile has been loaded into the program choose the fitting model spheres with lognormal size distribution (click Calc button, Single Data Set button, Fit button). Fixed parameters are p =1 and the contrast of silver in water eta = 6.7934e+011 cm^{-2}). The fitted parameters are N =2.57922e-25, s = 0.21911 and mu = 2.71641. (c) The resulting fit curve and data set can be saved to a file via File > Export > *I(q)* or by moving the curser to the

curve, click right mouse button and chose one of the given options (e.g. copy to the clipboard). (d) The model fitted values and their estimated uncertainties are available at parameters of analytical size distribution. The size distribution curve data can be saved via File > Export < N(R)

3. Data evaluation with McSAS (from RoundRobinMcSAS notebook)

Here, we focus on the results from McSAS. These results are stored alongside each .csv dataset, in a subdirectory. We read the two result files, one for the number-weighted, and one for the volume-weighted population statistics. We then calculate the deviation from the median mean, and the deviation from the median variance for each dataset, adding them to the table for subsequent plotting. Based on these datasets, we can do some simple listing. Of note is that the datasets measured by some laboratories on their second instrument, has had "100" added to the dataset number.

Volume-weighted results

The following section displays the results from the McSAS fitting procedure. This section in particular focuses on the volume-weighted parameters (mean, variance), the next will consider the number-weighted distribution modes. The plot shows the fractional deviation of each datasets from the median mean, and median variance.

It is possible to filter the datasets based on various criteria. This has been demonstrated for two selectors: outliers are rejected, and the plotting is repeated twice to distinguish between laboratory-based instruments and synchrotron-based instruments.

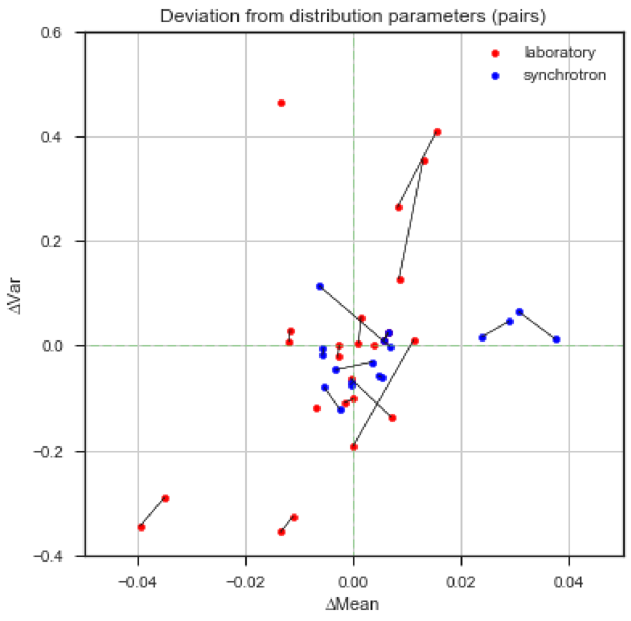

Figure S 5. Measurement-specific deviation from the median mean and median variance values (volume-weighted).

We can also determine some statistical values that are indicative of the spread of the results. The results from the various calculation methods below varies a little, and therefore these have not been used in the publication. Instead, the published results on the spread follow the analysis procedure of SASfit and GIFT.

Number-weighted values

Here, we do the same for the number-weighted values. A distinct correlation can be seen between the deviation in the variance, and the deviation from the mean. This highlights that the transformation (in McSAS at least) to number-weighted values brings with it a significant bias. This is likely due to the surface- or volume-weighted nature of the SAXS-data from size-disperse samples.

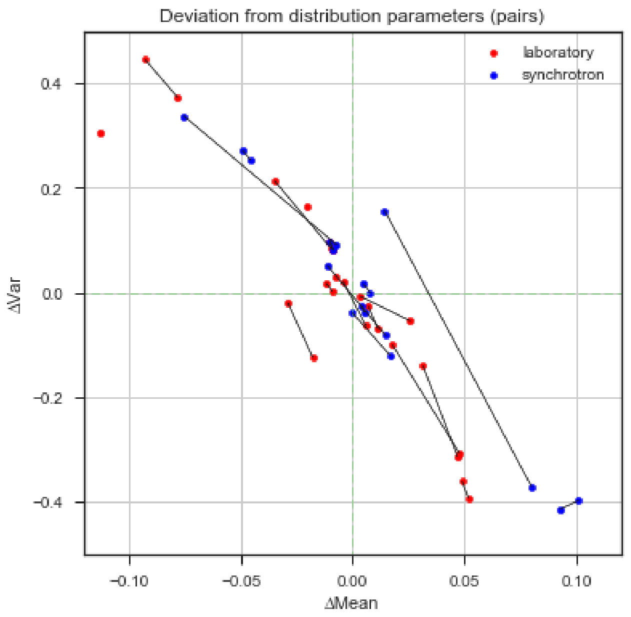

Figure S 6. Measurement-specific deviation from the median mean and median variance values (volume-weighted). This time, the data shows a clear correlation between the deviation in the mean and the deviation in the variance, demonstrating that number-weighting is less appropriate for SAXS data.

Absolute intensities / total mass fractions

We also want to analyze the spread of the volume fractions. This is only applicable to the laboratories which provided their data in absolute units. For each of these, the contrast value has been determined for the photon energy that was used.

It was expected that the spread might be dependent on whether the instrument was based in a laboratory or at the synchrotron. However, within the sample size, the results between the two do not vary significantly.

Two plots are shown, one showing the separate volume fraction results between laboratories and synchrotrons (Figure S 7), and one showing the mass fraction of all the laboratories (**Figure S 8**).

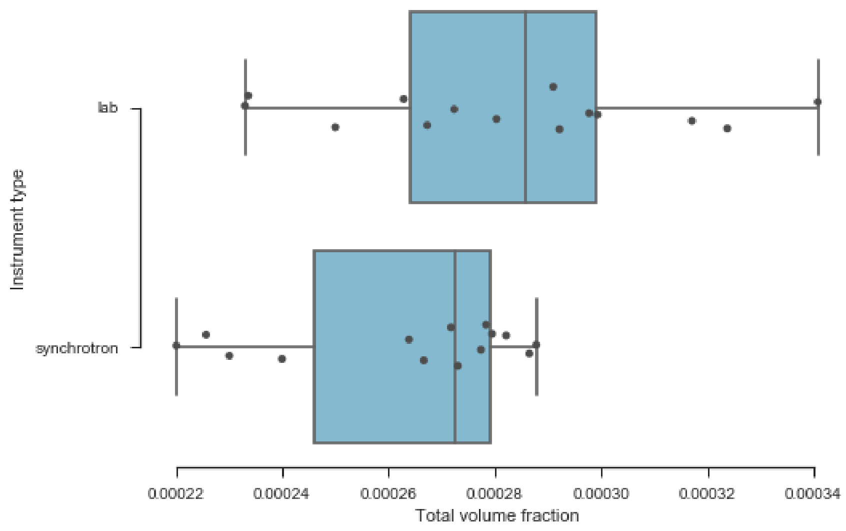

Figure S 7. The spread between the found volume fractions, showing no significant difference between the synchrotron and laboratory measurements.

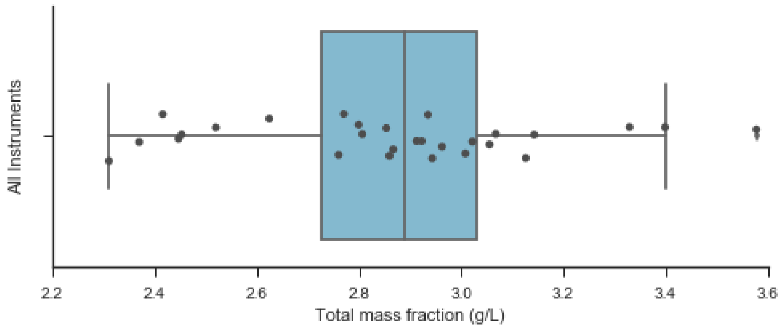

Figure S 8. The mass fractions determined from all the absolute-scale measurements.

Statistics on mass fractions

We also determine some indicative values of the spread of the mass fraction results, calculated using two different methods.

1 standard deviation from pop (abs. int., relative): 0.111

1 standard deviation (abs. int., relative): 0.108

4. An assessment of the calibration of, and uncertainty in q

Introduction

The core determinant for the precision and accuracy in the established radii is the variation we might encounter in q. This encompasses the shift and scaling of the q-axis, the per-data point accuracy in position, as well as the spread of arriving photons in q. The latter is colloquially known as "smearing", effecting a blurring, or smoothing of the scattering pattern. We will address each of these contributions.

It should be noted that we are using the term “uncertainty” for all effects on q in this evaluation. From the viewpoint of the single photon, this is correct: each arriving photon carries a scattering vector value which is made less precise due to all the effects mentioned below. From the viewpoint of the scattering pattern, consisting of a multitude of detected photons this is not quite correct: the smearing effects result in a population spread of the arriving photon’s scattering vectors, which is well defined and whose precision can be improved with knowledge on the smearing contributors (e.g. by means of the previously mentioned “desmearing” methods). Uncertainty resulting from other contributors, such as the uncertainty due to finite pixel sizes, cannot be reduced in this manner. For this estimation, however, we treat all as pure uncertainty contributors, and do not distinguish between the two variants.

The smearing

The smearing occurs due to uncertainties in the q value of a given detected photon. Over an ensemble of photons, this arrival position uncertainty distribution is typically centrosymmetric and ideally narrow. It leads to a reduction of definition (sharpness) in the detected pattern, similar to the "blurring" effect in image editing software, and is colloquially known as

"smearing". Analogous to the image editing example, the process can be partially reverted through an edge sharpening operation, in scattering parlance referred to as "desmearing". This desmearing process, however, is mathematically challenging, magnifies uncertainties, and can introduce or miss features in the data.

Smearing has a few causes, such as practical geometric limitations, wavelength dispersion, and detector imprecision. The centrosymmetric nature of the effect, and the optional desmearing step, however, can practically reduce the significance of the smearing effects. Nevertheless, it is always prudent to estimate the severity of the smearing contributions in order to provide a ballpark figure for the real uncertainty.

The per-data point position accuracy

One of the contributors to the smearing mentioned above is not just part of the smearing contribution, but also forms the lower limit of the uncertainty in q. Its fundament lies in the accumulation of photons with almost-but-not-quite-identical q values for a given data point. This per-data point position uncertainty is typically determined by the detector pixel size for raw data from pixel detectors, or bin size for binned data.

The scaling and shifting accuracies

For every instrument, practical limits on the knowledge of the instrument geometry leads to an uncertainty in the scaling and shift of the q axis. It is possible to estimate these two contributions practically using available calibration samples.

The plan

This section evaluates each of the three main effects in turn. We will first estimate the effects contributing to the smearing of our data. This section also contains the per-data point uncertainty that forms the minimum q uncertainty, which will therefore not be repeated. Secondly, we show our practical estimations for the scaling and shifting accuracy using a variety of calibration samples. Lastly, we will estimate the effect of these uncertainties on the size distribution parameters derived from a scattering pattern of silver nanoparticles.

The smearing

The geometry of the instrument

The Kratky camera employed for our measurements, has an approximate (fixed) sample-to-detector distance l_{sd} of 307 mm and a sample radius r_s of 1 mm. The pixel size on the Dectris Mythen detector attached to our Kratky camera is 50 µm, with no point-spread function. The X-ray spectrum reflected by the multilayer optics largely consists of Copper $k_{\alpha 1}$ and $k_{\alpha 2}$ radiation, whose mixture gives us a weighted apparent wavelength of 0.1542(2) nm. When we assume perpendicularity of the detector with the point of normal incidence at the beam center, the $q_{pixel} = \frac{4\pi}{\lambda \sin(\theta_{pixel})} = 6.64 \times 10^{-3}$ nm^{-1}. The useful data in these experiments has been defined to start at $q = 0.1$ nm^{-1}. Presented data has been desmeared using the procedure implemented in the Anton Paar SAXSQuant software accompanying the instrument. For this desmearing procedure, a beam profile has been collected using the employed collimation settings. Three desmearing iterations have been used.

The finite sample thickness contribution

We can determine the sample thickness-induced uncertainty in $q, \Delta q$, using:

$$\Delta q = 4\pi\lambda\ (\sin\theta_1 - \sin\theta_2)$$

where

$$\theta_1 = \frac{1}{2}\arctan\left(\frac{l_d}{l_{sd} - r_s}\right)$$

and

$$\theta_2 = \frac{1}{2}\arctan\left(\frac{l_d}{l_{sd} + r_s}\right)$$

where l_d is the distance on the detector from beam center to the point of interest.

We calculate l_d from our reference q vector using:

$$l_d = l_s d \tan\left(2\arcsin\left(\frac{q\lambda}{4\pi}\right)\right)$$

Δq due to the sample thickness: 2.80×10^{-3} nm^{-1}.

The finite beam height contribution

This is the issue of the beam not being infinitely thin. That means that scattering originates from multiple heights in the sample. This is calculated virtually identically to the above correction, except that we are adding the radius of the beam r_b to l_d instead of $l_s d$:

$$\theta_1 = \frac{1}{2}\arctan\left(\frac{l_d - r_b}{l_{sd}}\right)$$

and

$$\theta_2 = \frac{1}{2}\arctan\left(\frac{l_d + r_b}{l_{sd}}\right)$$

Δq due to beam finite beam height: 3.98 × 10^{-2} nm^{-1}.

The uncertainty originating from the beam divergence

The beam divergence is dictated by the collimation as well as the optics. In our instrument, the beam is focused on the beamstop, and using that information together with the width of the beam at the sample position, allows us to estimate the effect of divergence. This calculation can be done quite similar to the estimates from before, but where we add the divergence angle from the straight beam θ_d to the angles:

$$\theta_1 = \frac{1}{2}\arctan\left(\frac{l_d}{l_{sd}}\right) + \theta_d$$

and

$$\theta_1 = \frac{1}{2}\arctan\left(\frac{l_d}{l_{sd}}\right) - \theta_d$$

where

$$\theta_d = \arctan\left(\frac{r_b}{l_{sd}}\right)$$

Δq due to beam divergence: 0.185 nm^{-1}.

The polychromaticity contribution

The emission lines reflected by the mirror contain both the $k_{\alpha,1}$ as well as the $k_{\alpha,2}$ energies (actually, we find that the mirror also reflects small amounts of higher order energies from bremsstrahlung, but these are not significant for the discussion on the polychromaticity

contributions). These two energies bring about a small change in wavelength, and therefore q-vector. It is very small, but we include it here for completeness.

We estimate the thereby induced uncertainty in q, Δq, using:

$$\Delta q = (\frac{4\pi}{\lambda_1} - \frac{4\pi}{\lambda_2})\sin\theta$$

where

$$\theta = \arcsin\left(\frac{q\lambda}{4\pi}\right)$$

Copper $k_{\alpha,1}$ wavelength: 1.5405 × 10^{-10} nm, Copper $k_{\alpha,2}$ wavelength: 1.5444 × 10^{-10} nm

Relative uncertainty $\Delta q/q$ due to wavelength differences: 2.49 × 10^{-3}.

The uncertainties originating from pixel or data point widths

The finite pixel or data point size gives us a width in q that can affect the resolution of the smaller scattering vectors.

We distinguish between pixels and data points, as the data in multiple pixels may have ended up in a single data point during the averaging or re-binning procedure. Geometrically, we have a lower limit of the precision defined by the pixel width. As given in the geometry section, this means that we have an uncertainty of no less than $6.6 \times 10^{-3}\mathrm{nm}^{-1}$ at $q = 0.1$, or 7 %. For the pixels higher up in q, this uncertainty will gradually decrease.

The minimum q-point distance in our example, binned dataset is the same at $q = 0.1$, implying that we have not lost precision through the re-binning procedure for the first data point. For the later data points, however, more and more pixels have been grouped together to form a single data point.

Along the q-vector of our measurement, the relative $\Delta q/q$ span of each data point can be determined:

Mean relative uncertainty introduced by the binning (full width): 3.53%.

Smearing effect evaluation:

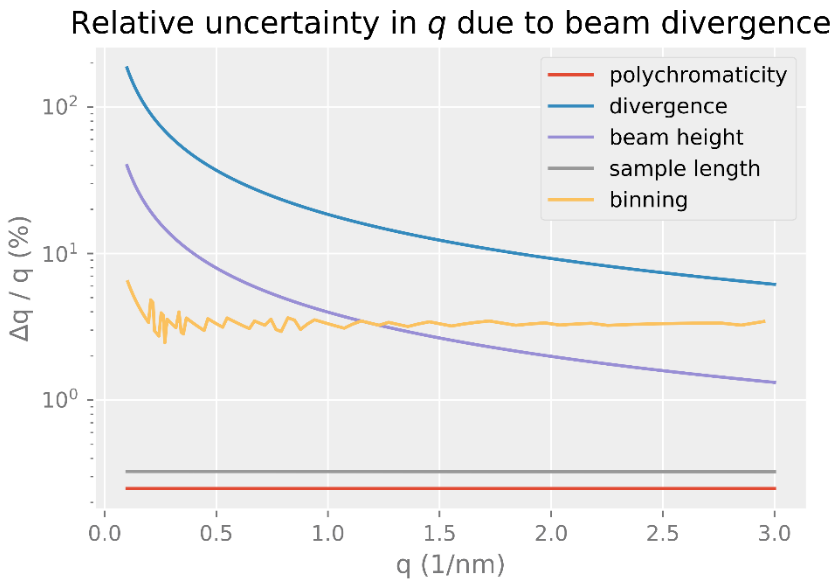

Figure S 9. Relative contribution of each uncertainty contributor to *q*.

Using the largest estimator (that of the divergence), the pattern of the round-robin silver nanoparticle dispersion becomes:

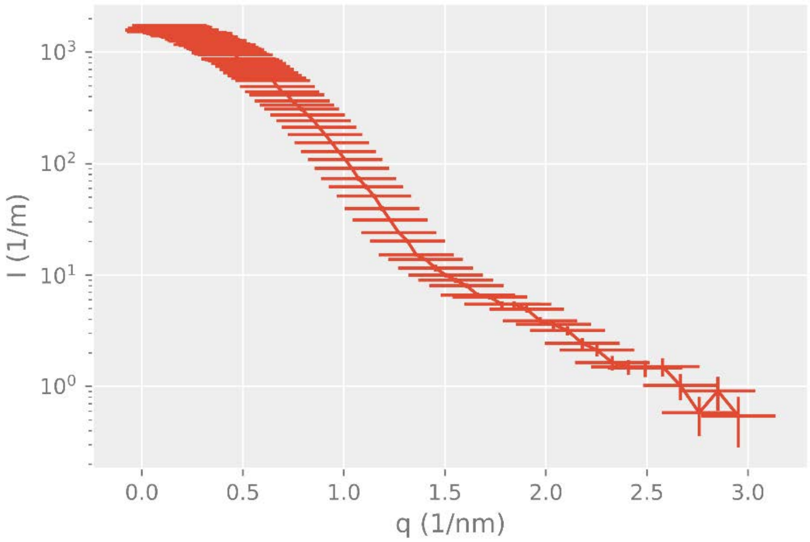

Figure S 10. The effect of divergence on an example scattering pattern: a smearing with the broadness as indicated in the plot.

Discussion

There is only one dominant uncertainty contributor to the smearing contribution, which is the divergence. As this contribution is significantly larger than the remaining contributors, the total uncertainty budget is accurately described by this sole contributor, and a calculation of the total uncertainty is not necessary.

The effect of the divergence can be shown on our example silver nanoparticle dataset in Figure S 10, where it is a significant contribution. Indeed, it is so large that it raises questions about the validity of the estimation in practice. The effect in practice would be a broadening or smearing of the scattering pattern by a given divergence distribution function, the width of which is as described above.

Some possible arguments on why it may not be as bad as this worst case scenario could be: 1) The beam convergence angle is weighted towards the horizontal, and so it is not as bad as

predicted, or 2) We did not take into account the single-sided limitation to the divergence introduced by the Kratky collimation.

The worst of the arguments (in terms of traceability) is that we perform desmearing on the data received from the instrument. That means that *some* of the broadening in q has been counteracted at the penalty of higher uncertainties in the intensity. Which of the various smearing effects, besides the beam width, are correlated, affected and/or mitigated by this feature-sharpening procedure is not fully understood by the author. The thus-introduced reduction in our q uncertainty is very difficult to assess. We are therefore in this case limited to using practical estimators for the uncertainties, at least in the case of the Kratky-class instruments. A detailed analysis of resolution and flux of slit collimation instruments is given by Fritz et. al. (Fritz & Bergmann, 2006).

For an instrument based on a parallel beam, we could consider evaluation of the direct beam dimensions on the detector, which immediately encompasses three out of five of the considered effects. This is not possible in this case, as the focusing of the beam on the beamstop (close to the detector) "hides" the divergence effect.

Instead, we would like to use an evaluation sample which would show narrow diffraction lines on the detector such as tendons or optical gratings. These samples would allow evaluation of the broadening effects of the beam. However, these are not at hand for our slit-focused instrument.

Of note is the uncertainty introduced by the data point binning. This is not only a smearing contributor, but also the lower limit of the real uncertainty in q. Using the current binning scheme, it is a constant contribution at about 3.5% of the q value.

The scaling and shifting accuracies

The accuracy of the scaling and shift our q vector may be assessed using calibrators. These are:

1. Calibrant-based: silver behenate, apoferritin, glassy carbon

2. Geometrical considerations

The purpose of these calibrants is to determine the practical variance in q position for data points along the detector, and the shift of the entire q scale.

Evaluation of the effects of the uncertainties in *q*

We measured silver behenate, apoferritin and glassy carbon to practically assess the calibration and uncertainties of q.

Silver behenate is a commonly used sample for distance calibration, and is particularly convenient on the Kratky camera since it allows the assessment of a total of six peaks within the measurement range. Comparing the positions of the six peak maxima with the expected values allows the proving of two geometrical assumptions: the sample-to-detector distance, and the beam centre position. An offset in the beam centre will show up as a consistent divergence of the subsequent detected reflections with respect to their expected values. Likewise, the determination of the maxima will give us not an accurate, but at least a workable estimate on the data point q variance.

One criticism of silver behenate as an absolute q calibrant is its instability: it can absorb water, thereby changing the crystal lattice somewhat. Some have indicated its components might also be degraded (affected) by light. An alternative calibrant, available in large quantities and of very well-defined shape is (equestrian) apoferritin. This core-shell protein exhibits clear features in the scattering pattern and scatters well. Its fundamental pattern can be calculated

using existing bead-model techniques, and modeled using a core-shell sphere function. We use this here to double-check our silver behenate calibration.

Glassy carbon is not typically used for q calibration, being primarily intended for absolute intensity calibration. However, based on the initial results in the subsequent experiments, it may be possible to use this for both with reasonable accuracy. Its easy availability, high scattering, stability and solid state makes it ideally suitable for calibrating instruments without liquid handling facilities.

Silver behenate

We evaluate the q-uncertainty along the detector of silver behenate by determining the q-position of the data point with the largest intensity for each peak. No peak fitting is done (on purpose), as we are trying to simultaneously assess the worst-case practical q-uncertainty between data points in this way. Unfortunately, we cannot easily maintain the same binning as for the "standard" dataset (i.e. a goal of 100 data points for the range 0.1 - 3 nm^{-1}) as the peaks span a much different range. Instead, we will consider the 3.5% q-uncertainty introduced by the standard binning procedure to be a lower limit of the uncertainty estimate determined herein.

The q-positions of the data points with maximum intensity in the silver behenate sample are:

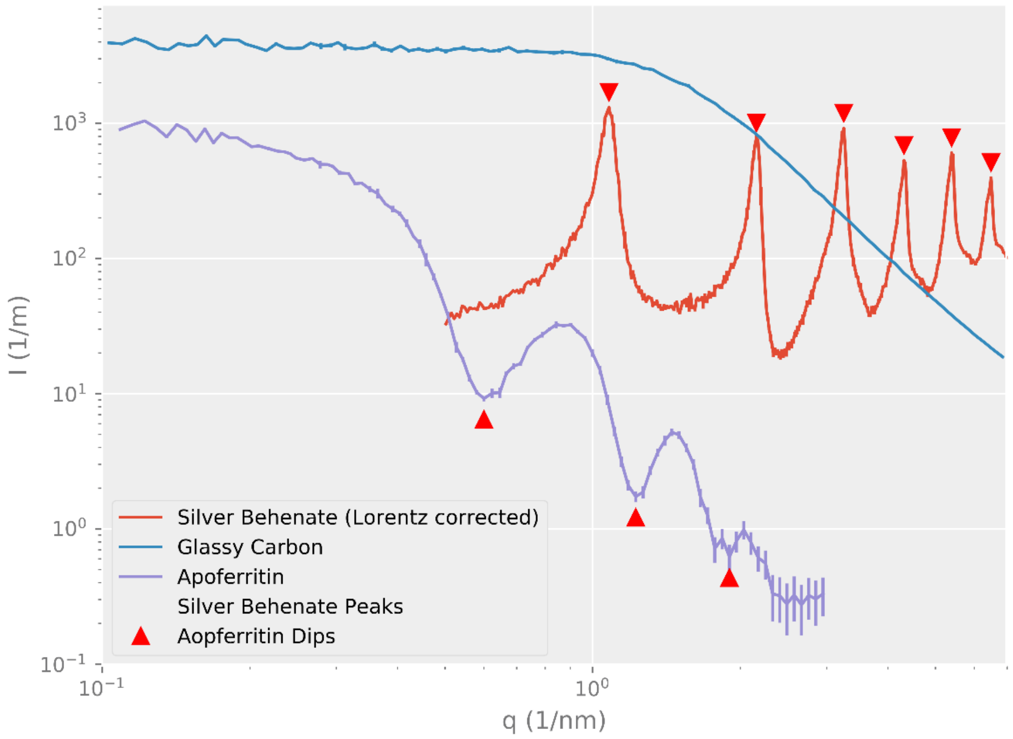

Figure S 11. The practical calibrators used: glassy carbon, silver behenate (Lorentz-corrected), and Apoferritin. Maxima and minima of the latter two have been indicated with red triangles.

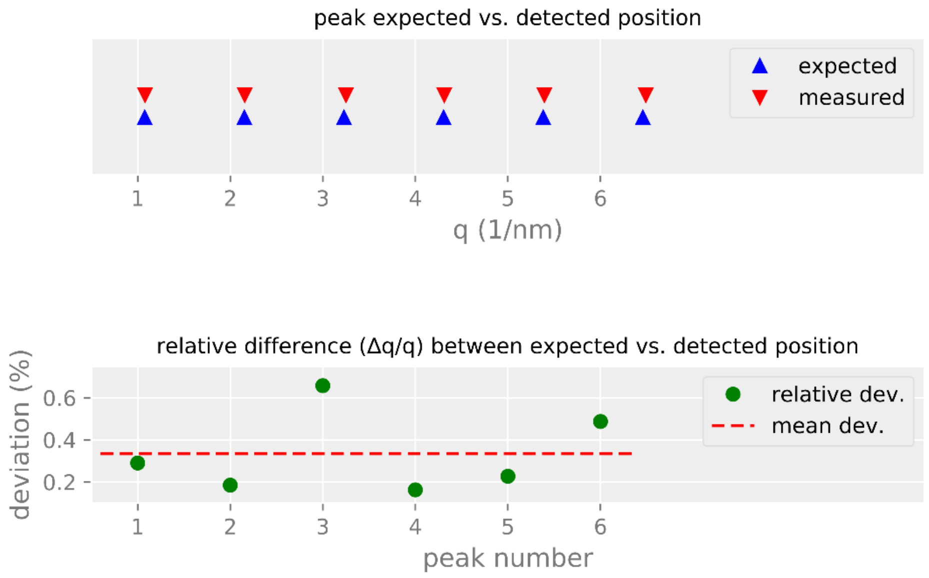

Figure S 12. Top: Expected versus positions detected in practice, of the silver behenate peaks. Bottom: Relative difference of the expected versus detected position of the silver behenate, hinting towards a slight miscalibration (0.35%).

This result shows that our q-calibration is a slightly off: the measured peak positions are consistently higher than the expected values. However, this deviation is an order of magnitude less than the position uncertainty introduced by the binning (3.5%), and therefore well within limits.

Apoferritin

Apoferritin is not as popular as silver behenate as a calibrant, but it is more convenient in several ways:

1. Its scattering pattern can be calculated from crystallographic models to good agreement.
2. It has a distinct feature (dip) in the scattering pattern at lower q than silver behenate.

3. It is very stable, with a well-defined structure in solution.

4. Its liquid state means it is easily injected into an existing liquid-sample configured instrument.

5. It scatters quite strongly, requiring little measurement time.

6. It is quite cheap.

To model it, we follow Zhou et al. (Zhou *et al.*, 2005) and Leiterer et al. (Leiterer *et al.*, 2008) and approximate its structure as a core-shell sphere with an inner radius of 3.75 nm, and an outer radius of 6.2 nm. We then use the pattern-shifting method to see how far we are off in our calibration. We apply the same using a CRYSOL-modeled scattering pattern from the published PDB structure.

The pattern-shifting method

In this method (also introduced here), we take a given measured pattern, complete with uncertainties, and compare it to a calibration dataset. The measured pattern or calibration dataset is shifted by a given amount in q. We then compare the fit of the shifted pattern after scaling the intensity, and calculate the goodness-of-fit parameter χ_r^2. In order to calculate this, we do need to perform a (linear) interpolation on the intensity over q, along with the uncertainties, which can create a bit of numerical "noise" in the result.

For a pattern for which there is no perfectly matching calibration dataset, we can only determine the best-fit q-shift. This can serve as a basic q-calibration method. One remaining way to then estimate the uncertainty on the fitting parameter, is to find the width at $\chi_r^2 = 2\chi_{r,min}^2$. This is a method used in, for example, p.62 of the Ph.D. thesis of Gleber (Gleber, 2013).

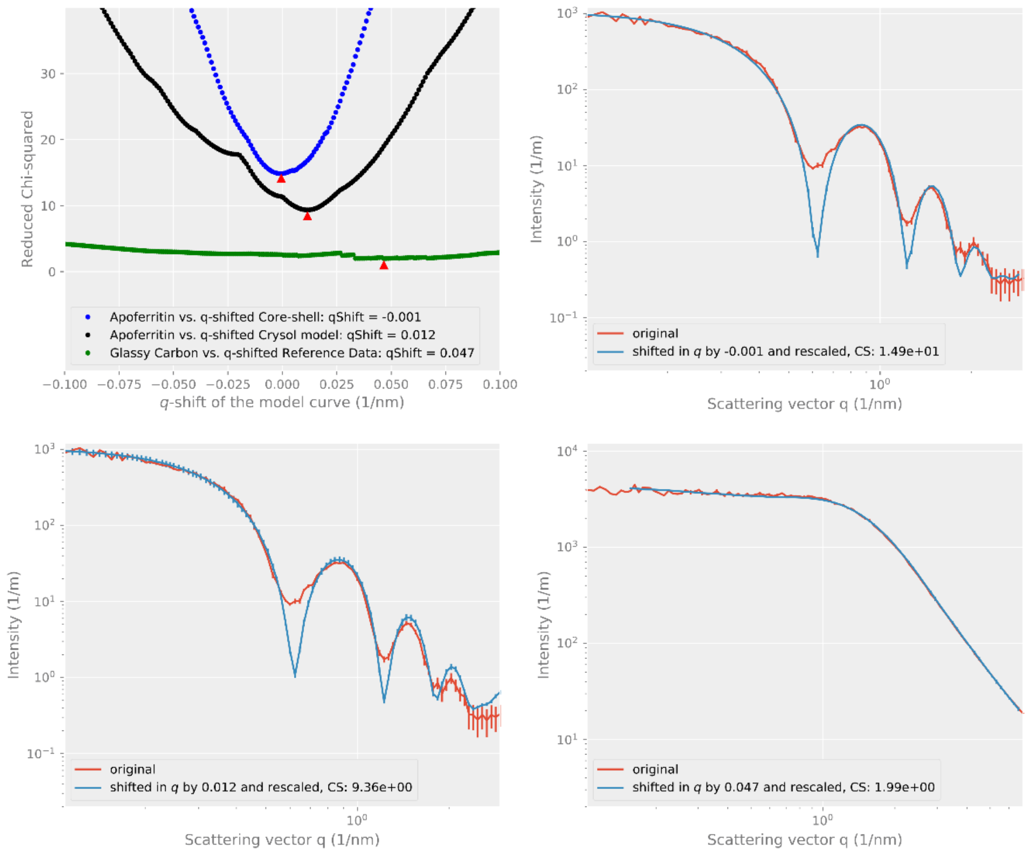

Figure S 13. Top left: The reduced chi-squared values resulting from the pattern-shift method, applied to Apoferritin vs. models, and glassy carbon vs. its calibration dataset. Top right: The Apoferritin data compared to the core-shell model, shifted by its best-fit in *q*. Bottom left: The Apoferritin data compared to the Crysol model, shifted by its best-fit in *q*. Bottom right: The glassy carbon dataset compared to the reference dataset, shifted by its best-fit in *q*.

This shift method shows that our data is well aligned to match the core-shell approximation to the apoferritin model, and confirms that any shift in our *q* axis is well below the size in *q* that our data points describe, although it is hard to estimate the variance using this simplified model. Similar information is obtained when we apply the same method using a scattering curve

calculated from the PDB crystal structure, although that pattern appears to be shifted by 0.012 nm^{-1}, which is more than 50% smaller than the resolution at which the CRYSOL model has been calculated.

Estimating the mean for both apoferritin fit curves above at the position where χ_r^2=2$\chi_{r,min}^2$, that would be an uncertainty of $\approx \pm$ 0.035 (estimates are 0.03 and 0.04 for the curves).

Glassy carbon

Of interest is to note that, when we consider all the data points as an ensemble, we are very sensitive to shifts of the entire axis. This indicates that we may even be able to use glassy carbon as our calibration sample. Like apoferritin, we are not using it to determine the sample-to-detector distance, but rather the shift of the entire q axis.

Unfortunately, the results for glassy carbon are nowhere near as nice as they could be. The results show that we have a shift of nearly 0.05 nm^{-1}, and are still not reaching a $\chi_r^2 \leq 1$. This is far out of line of the results from both the silver behenate as well as the apoferritin, and therefore we tentatively conclude that glassy carbon does not lend itself well to calibration of the q axis. On a positive note, our absolute intensity calibration using water scales the subsequently measured glassy carbon data to within 10% of its reference values.

Evaluation of the worst-case (divergence-limited) scenario

In order to estimate the effect of the divergence-induced shift on the size determination, we shifted the scattering pattern collected on the Kratky instrument from the Round Robin silver nanoparticle dispersion in q. Two patterns were thus generated, one with a negative shift by half the divergence-induced shift, and one with a positive shift by the same amount.

Note: the fits were performed on a reduced number of data points, to ensure the approximate same minimum q is used for the fits (the negatively-shifted data otherwise contains excessively small q values).

Evaluation of the calibration-limited scenarios

Another estimate would be using the largest realistic value from the practical q-shift measurements, i.e. the one determined from the shift between the apoferritin data and the CRYSOL model, which is q_{shift} = 0.012 nm^{-1}. As before, all analyses have their low-q value limited to remove range inconsistencies.

The second calibration-derived estimate used is the shift of q_{shift} = 0.035 nm^{-1}, as deduced from the calibration of apoferritin.

Evaluation of the binning-limited scenario

The last estimate uses simply the binning-induced uncertainty in q. As this width is a constant fraction (3.5%) of the scattering pattern rather than a shift, the estimation is done on patterns whose q-vector has been magnified and shrunk by 1.75%.

The effect of this on the results is as follows:

- The mean radius determined from the shrunk, normal and magnified q axes are: 3.267(4), 3.214(3), 3.186(2) nm
- The width (square root of the variance) of the same, are: 7(1), 6.7(9), 6.5(8) nm

The binning, therefore, introduces an uncertainty of approximately -1/+2 % in the mean, which is the same magnitude as the round-robin uncertainty. Concerning the distribution widths, the

binning appears to introduce an uncertainty of -8/+6 %. This is, however, significantly smaller than the uncertainty in determination of the widths.

Table S 4. Effects of various *q*-shifts on the resulting McSAS fitting parameters. Sigma is the square root of the variance.

Shift (nm^{-1})	Mean (m)	Mean Uncertainty (m)	Variance (m^2)	Variance Uncertainty (m^2)	Sigma (m)	Sigma Uncertainty (m)
0.0000	3.21e-09	2.56e-12	4.53e-19	8.44e-21	6.73e-10	9.19e-11
-0.0120	3.24e-09	4.01e-12	5.04e-19	1.40e-20	7.10e-10	1.18e-10
0.0120	3.19e-09	1.86e-12	4.17e-19	7.13e-21	6.46e-10	8.44e-11
-0.0350	3.28e-09	5.41e-12	6.08e-19	2.10e-20	7.80e-10	1.45e-10
0.0350	3.13e-09	1.46e-12	3.59e-19	4.98e-21	6.00e-10	7.05e-11
-0.0925	3.38e-09	1.33e-11	8.99e-19	4.43e-20	9.48e-10	2.10e-10
0.0925	3.04e-09	9.19e-13	6.95e-19	9.22e-21	8.34e-10	9.60e-11

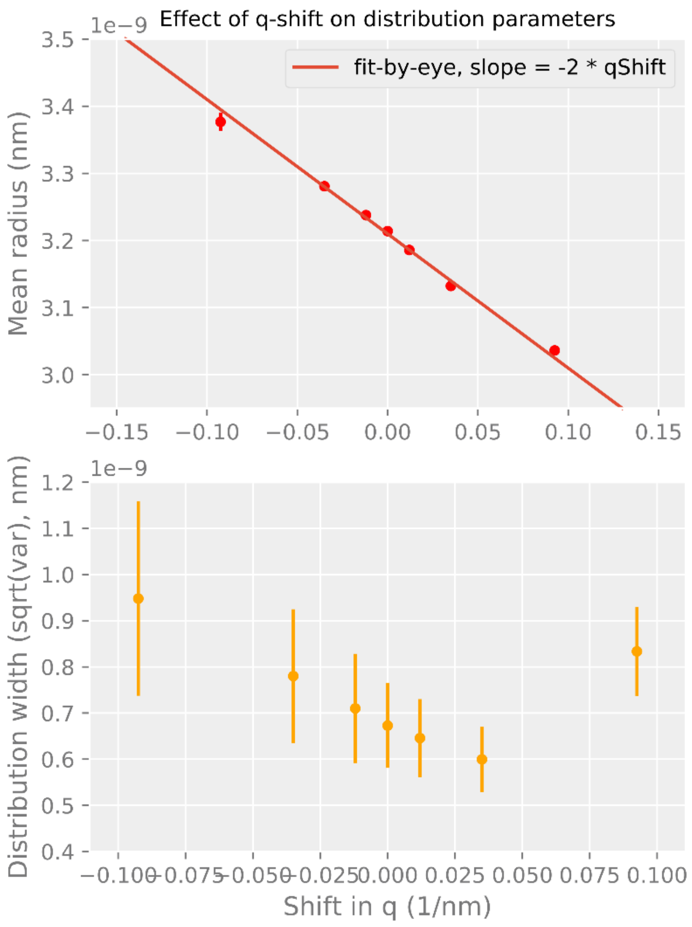

Figure S 14. The effect of a shift in q on the McSAS-determined mean radius (top) and distribution widths (bottom).

Overall uncertainty estimate

The silver behenate measurement shows that we are well within expected values for the peak positions, and so we conclude that the sample-to-detector distance is accurately defined. In other words, we have no uncertainty contributions leading to a shrinking or magnification of the scale outside from the binning-induced effects, and can concern ourselves mainly with the uncertainty in the q-shift of the entire axis.

The binning-induced (smallest possible) uncertainty on the q scaling is on the order of three percent (mostly defined by the data point width and the binning). This is superseded by the practically determined possible zero-q shift from the apoferritin measurement of up to 0.012 nm^{-1}. In total, these uncertainty contributions can affect the determined mean radii by about 1%, but the effect on the distribution width is within noise levels (Figure S 14, and Table S 4).

When we consider the zero-position uncertainty as derived from the χ_r^2-behaviour of the apoferritin models vs. the apoferritin measurement, we can estimate an uncertainty in the q shift of $q \pm 0.035\ \text{nm}^{-1}$. This can introduce a maximum uncertainty in the mean radius of up to 2.5%, and in the distribution width of up to 35% in the worst case.

Lastly, the divergence contribution is very large, introducing a potential smearing in the data with a width of almost 0.1 nm^{-1}. The effects of such a shift on the radii would be quite dramatic, but we assume that the effects thereof are centrosymmetric, and (largely) compensated by the desmearing procedure in this particular application.

The aforementioned q-uncertainty contributions can, therefore, realistically affect the determined distribution parameters by up to a few percent for the mean radius, and possibly up to 35% on the distribution width determination (worst case scenario). The latter, however, is hard to distinguish from the numerical noise inherent in its determination.

This uncertainty budget should ideally be recalculated for every measurement. It is expected to be less severe for instruments with a more parallel beam, and a higher data accuracy (allowing for smaller uncertainty estimates on the determined parameter). At the very least, this derivation is a valuable exercise which brings great insight into the instrumental errors which can constrain our technique.

4. References

Bressler, I., Kohlbrecher, J. & Thunemann, A. F. (2015). *Journal of Applied Crystallography* **48**, 1587-1598.

Fritz, G. & Bergmann, A. (2006). *Journal of Applied Crystallography* **39**, 64-71.

Gleber, G. (2013). thesis*,* Technische Universität Berlin, Berlin.

Leiterer, J., Delissen, F., Emmerling, F., Thunemann, A. F. & Panne, U. (2008). *Analytical and Bioanalytical Chemistry* **391**, 1221-1228.

Zhou, J., Deyhim, A., Krueger, S. & Gregurick, S. K. (2005). *Comput. Phys. Commun.* **170**, 186-204.
