# Supplementary material for: Nanoparticle size distribution quantification: results of a small-angle X-ray scattering inter-laboratory comparison
Source: J Appl Crystallogr. 2017 Aug 18;50(Pt 5):1280–8. doi: 10.1107/S160057671701010X (PMC5627679; doi:10.1107/S160057671701010X)
Supplement: Supplementary file 3 [file j-50-01280-sup2.zip › RRAnonData/csv/S10_2016-12-02_20-37-51/S10_2016-12-02_20-37-51.pdf]

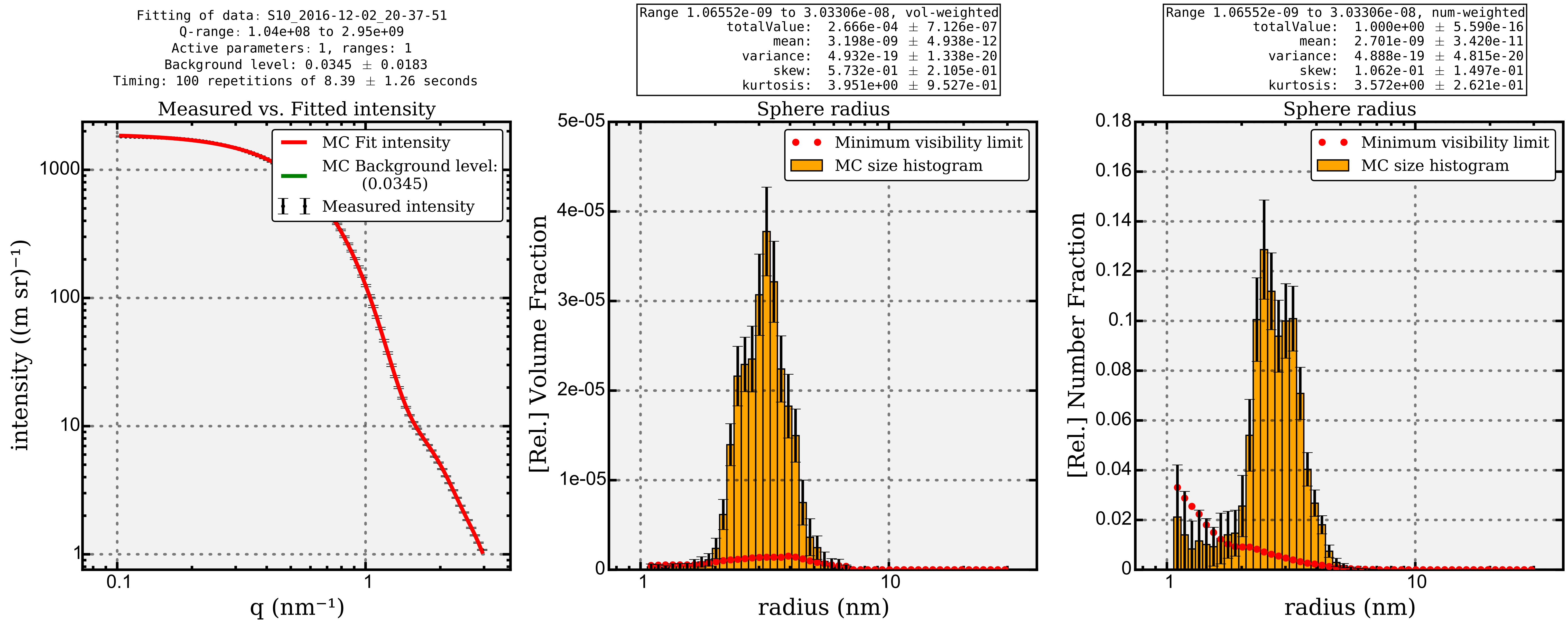
Fitting of data: S10_2016-12-02_20-37-51
Q-range: 1.04e+08 to 2.95e+09
Active parameters: 1, ranges: 1
Background level: 0.0345 ± 0.0183
Timing: 100 repetitions of 8.39 ± 1.26 seconds
Measured vs. Fitted intensity
MC Fit intensity
MC Background level: (0.0345)
Measured intensity
intensity ((m sr)⁻¹)
1000
100
10
1
0.1
1
q (nm⁻¹)
Range 1.06552e-09 to 3.03306e-08, vol-weighted
totalValue: 2.666e-04 ± 7.126e-07
mean: 3.198e-09 ± 4.938e-12
variance: 4.932e-19 ± 1.338e-20
skew: 5.732e-01 ± 2.105e-01
kurtosis: 3.951e+00 ± 9.527e-01
Sphere radius
Minimum visibility limit
MC size histogram
[Rel.] Volume Fraction
5e-05
4e-05
3e-05
2e-05
1e-05
0
1
10
radius (nm)
Range 1.06552e-09 to 3.03306e-08, num-weighted
totalValue: 1.000e+00 ± 5.590e-16
mean: 2.701e-09 ± 3.420e-11
variance: 4.888e-19 ± 4.815e-20
skew: 1.062e-01 ± 1.497e-01
kurtosis: 3.572e+00 ± 2.621e-01
Sphere radius
Minimum visibility limit
MC size histogram
[Rel.] Number Fraction
0.18
0.16
0.14
0.12
0.1
0.08
0.06
0.04
0.02
0
1
10
radius (nm)
