# Supplementary material for: Nanoparticle size distribution quantification: results of a small-angle X-ray scattering inter-laboratory comparison
Source: J Appl Crystallogr. 2017 Aug 18;50(Pt 5):1280–8. doi: 10.1107/S160057671701010X (PMC5627679; doi:10.1107/S160057671701010X)
Supplement: Supplementary file 3 [file j-50-01280-sup2.zip › RRAnonData/csv/S11_2016-12-02_20-52-01/S11_2016-12-02_20-52-01.pdf]

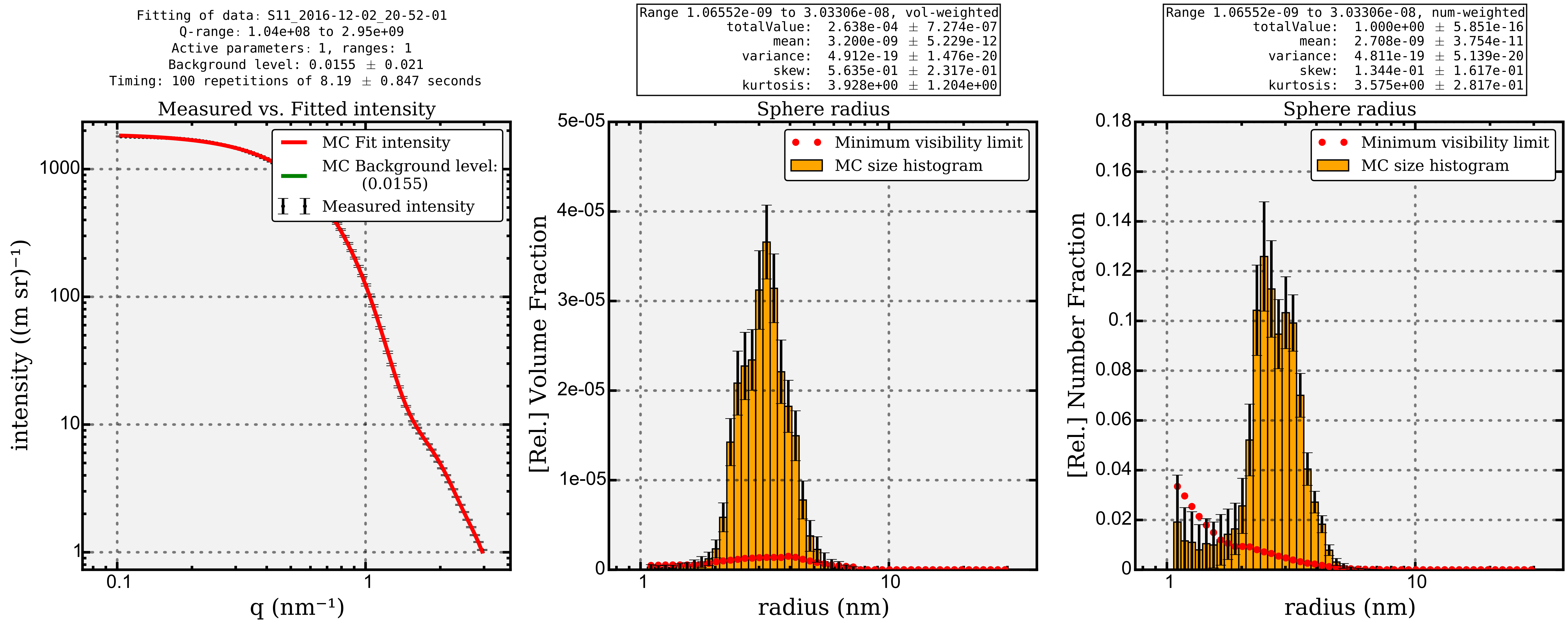
Fitting of data: S11_2016-12-02_20-52-01
Q-range: 1.04e+08 to 2.95e+09
Active parameters: 1, ranges: 1
Background level: 0.0155 ± 0.021
Timing: 100 repetitions of 8.19 ± 0.847 seconds
Measured vs. Fitted intensity
MC Fit intensity
MC Background level: (0.0155)
Measured intensity
intensity ((m sr)⁻¹)
q (nm⁻¹)
1000
100
10
1
0.1
1
Range 1.06552e-09 to 3.03306e-08, vol-weighted
totalValue: 2.638e-04 ± 7.274e-07
mean: 3.200e-09 ± 5.229e-12
variance: 4.912e-19 ± 1.476e-20
skew: 5.635e-01 ± 2.317e-01
kurtosis: 3.928e+00 ± 1.204e+00
Sphere radius
Minimum visibility limit
MC size histogram
[Rel.] Volume Fraction
radius (nm)
5e-05
4e-05
3e-05
2e-05
1e-05
0
1
10
Range 1.06552e-09 to 3.03306e-08, num-weighted
totalValue: 1.000e+00 ± 5.851e-16
mean: 2.708e-09 ± 3.754e-11
variance: 4.811e-19 ± 5.139e-20
skew: 1.344e-01 ± 1.617e-01
kurtosis: 3.575e+00 ± 2.817e-01
Sphere radius
Minimum visibility limit
MC size histogram
[Rel.] Number Fraction
radius (nm)
0.18
0.16
0.14
0.12
0.1
0.08
0.06
0.04
0.02
0
1
10
