# Supplementary material for: Nanoparticle size distribution quantification: results of a small-angle X-ray scattering inter-laboratory comparison
Source: J Appl Crystallogr. 2017 Aug 18;50(Pt 5):1280–8. doi: 10.1107/S160057671701010X (PMC5627679; doi:10.1107/S160057671701010X)
Supplement: Supplementary file 3 [file j-50-01280-sup2.zip › RRAnonData/csv/S149_2016-12-03_10-23-18/S149_2016-12-03_10-23-18.pdf]

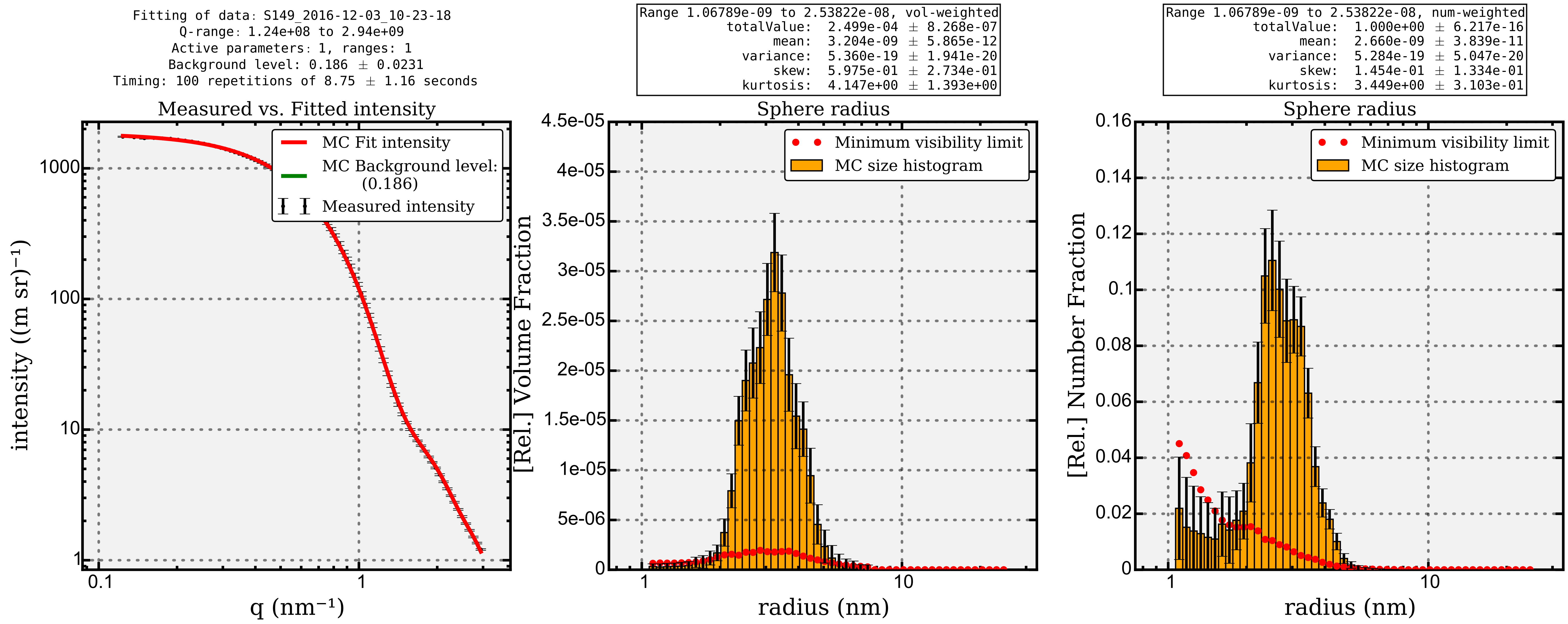

Fitting of data: S149_2016-12-03_10-23-18
Q-range: 1.24e+08 to 2.94e+09
Active parameters: 1, ranges: 1
Background level: 0.186 ± 0.0231
Timing: 100 repetitions of 8.75 ± 1.16 seconds
Measured vs. Fitted intensity
MC Fit intensity
MC Background level: (0.186)
Measured intensity
intensity ((m sr)⁻¹)
q (nm⁻¹)
Range 1.06789e-09 to 2.53822e-08, vol-weighted
totalValue: 2.499e-04 ± 8.268e-07
mean: 3.204e-09 ± 5.865e-12
variance: 5.360e-19 ± 1.941e-20
skew: 5.975e-01 ± 2.734e-01
kurtosis: 4.147e+00 ± 1.393e+00
Sphere radius
Minimum visibility limit
MC size histogram
[Rel.] Volume Fraction
radius (nm)
Range 1.06789e-09 to 2.53822e-08, num-weighted
totalValue: 1.000e+00 ± 6.217e-16
mean: 2.660e-09 ± 3.839e-11
variance: 5.284e-19 ± 5.047e-20
skew: 1.454e-01 ± 1.334e-01
kurtosis: 3.449e+00 ± 3.103e-01
Sphere radius
Minimum visibility limit
MC size histogram
[Rel.] Number Fraction
radius (nm)
