# Supplementary material for: Nanoparticle size distribution quantification: results of a small-angle X-ray scattering inter-laboratory comparison
Source: J Appl Crystallogr. 2017 Aug 18;50(Pt 5):1280–8. doi: 10.1107/S160057671701010X (PMC5627679; doi:10.1107/S160057671701010X)
Supplement: Supplementary file 3 [file j-50-01280-sup2.zip › RRAnonData/csv/S151_2016-12-03_10-38-11/S151_2016-12-03_10-38-11.pdf]

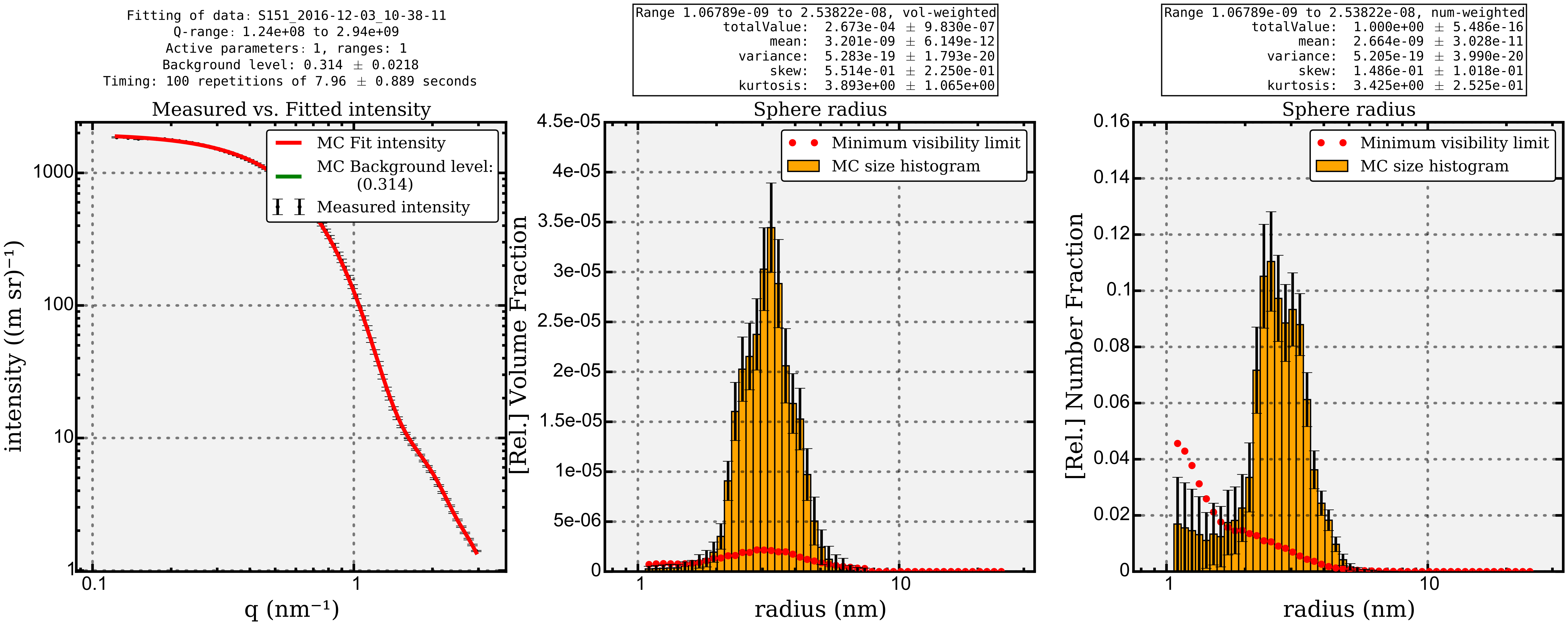
Fitting of data: S151_2016-12-03_10-38-11
Q-range: 1.24e+08 to 2.94e+09
Active parameters: 1, ranges: 1
Background level: 0.314 ± 0.0218
Timing: 100 repetitions of 7.96 ± 0.889 seconds
Measured vs. Fitted intensity
MC Fit intensity
MC Background level: (0.314)
Measured intensity
intensity ((m sr)$^{-1}$)
q (nm^{-1})
Range 1.06789e-09 to 2.53822e-08, vol-weighted
totalValue: 2.673e-04 ± 9.830e-07
mean: 3.201e-09 ± 6.149e-12
variance: 5.283e-19 ± 1.793e-20
skew: 5.514e-01 ± 2.250e-01
kurtosis: 3.893e+00 ± 1.065e+00
Sphere radius
Minimum visibility limit
MC size histogram
[Rel.] Volume Fraction
radius (nm)
Range 1.06789e-09 to 2.53822e-08, num-weighted
totalValue: 1.000e+00 ± 5.486e-16
mean: 2.664e-09 ± 3.028e-11
variance: 5.205e-19 ± 3.990e-20
skew: 1.486e-01 ± 1.018e-01
kurtosis: 3.425e+00 ± 2.525e-01
Sphere radius
Minimum visibility limit
MC size histogram
[Rel.] Number Fraction
radius (nm)
