# Supplementary material for: Nanoparticle size distribution quantification: results of a small-angle X-ray scattering inter-laboratory comparison
Source: J Appl Crystallogr. 2017 Aug 18;50(Pt 5):1280–8. doi: 10.1107/S160057671701010X (PMC5627679; doi:10.1107/S160057671701010X)
Supplement: Supplementary file 3 [file j-50-01280-sup2.zip › RRAnonData/csv/S155_2016-12-03_10-28-52/S155_2016-12-03_10-28-52.pdf]

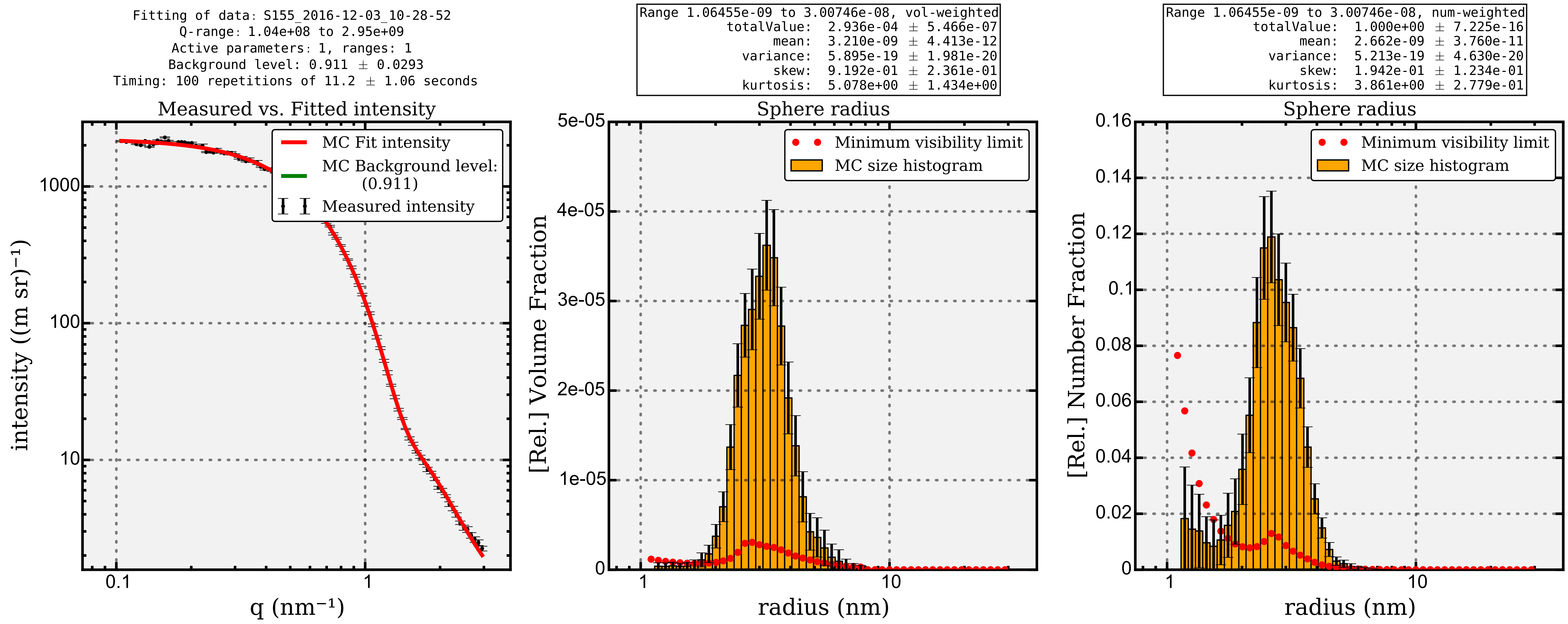
Fitting of data: S155_2016-12-03_10-28-52
Q-range: 1.04e+08 to 2.95e+09
Active parameters: 1, ranges: 1
Background level: 0.911 ± 0.0293
Timing: 100 repetitions of 11.2 ± 1.06 seconds
Measured vs. Fitted intensity
MC Fit intensity
MC Background level: (0.911)
Measured intensity
intensity ((m sr)$^{-1}$)
1000
100
10
0.1
1
q (nm^{-1})
Range 1.06455e-09 to 3.00746e-08, vol-weighted
totalValue: 2.936e-04 ± 5.466e-07
mean: 3.210e-09 ± 4.413e-12
variance: 5.895e-19 ± 1.981e-20
skew: 9.192e-01 ± 2.361e-01
kurtosis: 5.078e+00 ± 1.434e+00
Sphere radius
Minimum visibility limit
MC size histogram
[Rel.] Volume Fraction
5e-05
4e-05
3e-05
2e-05
1e-05
0
1
10
radius (nm)
Range 1.06455e-09 to 3.00746e-08, num-weighted
totalValue: 1.000e+00 ± 7.225e-16
mean: 2.662e-09 ± 3.760e-11
variance: 5.213e-19 ± 4.630e-20
skew: 1.942e-01 ± 1.234e-01
kurtosis: 3.861e+00 ± 2.779e-01
Sphere radius
Minimum visibility limit
MC size histogram
[Rel.] Number Fraction
0.16
0.14
0.12
0.1
0.08
0.06
0.04
0.02
0
1
10
radius (nm)
