# Supplementary material for: Nanoparticle size distribution quantification: results of a small-angle X-ray scattering inter-laboratory comparison
Source: J Appl Crystallogr. 2017 Aug 18;50(Pt 5):1280–8. doi: 10.1107/S160057671701010X (PMC5627679; doi:10.1107/S160057671701010X)
Supplement: Supplementary file 3 [file j-50-01280-sup2.zip › RRAnonData/csv/S156_2016-12-03_10-47-46/S156_2016-12-03_10-47-46.pdf]

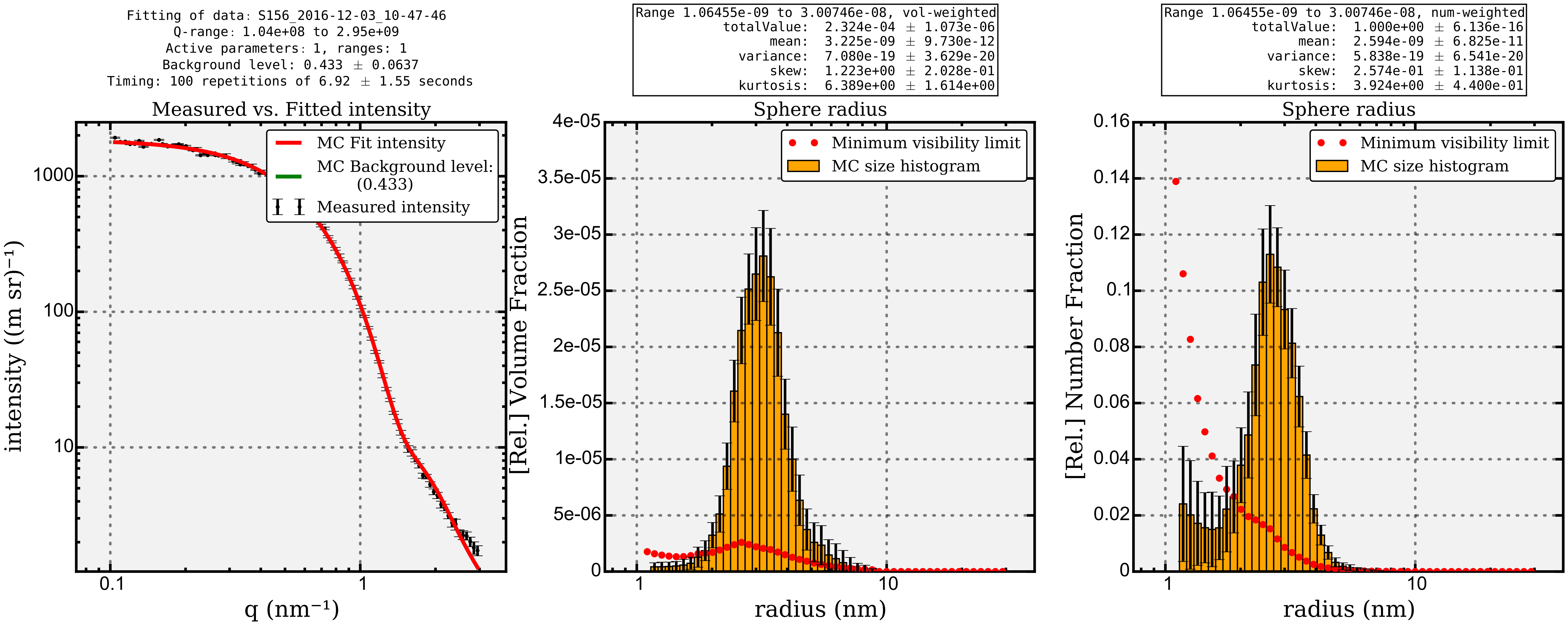
Fitting of data: S156_2016-12-03_10-47-46
Q-range: 1.04e+08 to 2.95e+09
Active parameters: 1, ranges: 1
Background level: 0.433 ± 0.0637
Timing: 100 repetitions of 6.92 ± 1.55 seconds
Measured vs. Fitted intensity
MC Fit intensity
MC Background level: (0.433)
Measured intensity
intensity ((m sr)$^{-1}$)
q (nm^{-1})
1000
100
10
0.1
1
Range 1.06455e-09 to 3.00746e-08, vol-weighted
totalValue: 2.324e-04 ± 1.073e-06
mean: 3.225e-09 ± 9.730e-12
variance: 7.080e-19 ± 3.629e-20
skew: 1.223e+00 ± 2.028e-01
kurtosis: 6.389e+00 ± 1.614e+00
Sphere radius
Minimum visibility limit
MC size histogram
[Rel.] Volume Fraction
radius (nm)
4e-05
3.5e-05
3e-05
2.5e-05
2e-05
1.5e-05
1e-05
5e-06
0
1
10
Range 1.06455e-09 to 3.00746e-08, num-weighted
totalValue: 1.000e+00 ± 6.136e-16
mean: 2.594e-09 ± 6.825e-11
variance: 5.838e-19 ± 6.541e-20
skew: 2.574e-01 ± 1.138e-01
kurtosis: 3.924e+00 ± 4.400e-01
Sphere radius
Minimum visibility limit
MC size histogram
[Rel.] Number Fraction
radius (nm)
0.16
0.14
0.12
0.1
0.08
0.06
0.04
0.02
0
1
10
