# Supplementary material for: Nanoparticle size distribution quantification: results of a small-angle X-ray scattering inter-laboratory comparison
Source: J Appl Crystallogr. 2017 Aug 18;50(Pt 5):1280–8. doi: 10.1107/S160057671701010X (PMC5627679; doi:10.1107/S160057671701010X)
Supplement: Supplementary file 3 [file j-50-01280-sup2.zip › RRAnonData/csv/S15_2016-12-02_20-59-20/S15_2016-12-02_20-59-20.pdf]

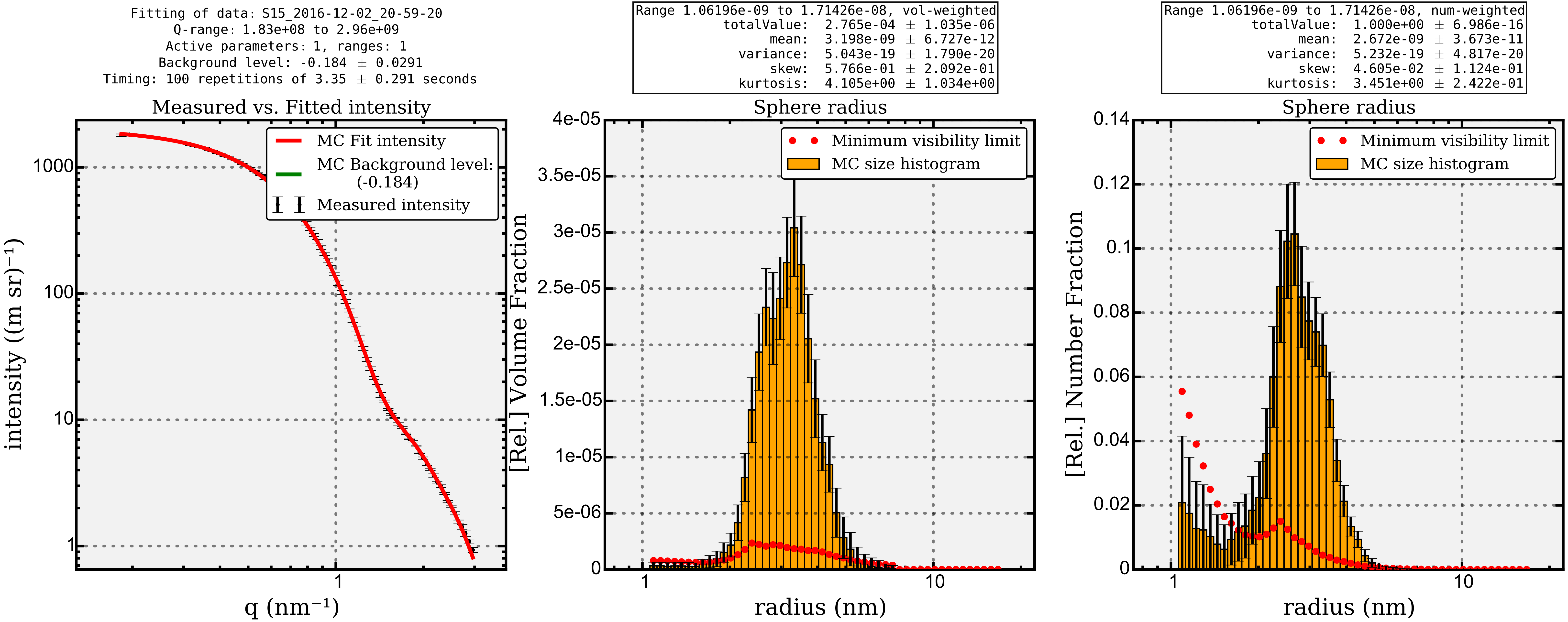
Fitting of data: S15_2016-12-02_20-59-20
Q-range: 1.83e+08 to 2.96e+09
Active parameters: 1, ranges: 1
Background level: -0.184 ± 0.0291
Timing: 100 repetitions of 3.35 ± 0.291 seconds
Measured vs. Fitted intensity
MC Fit intensity
MC Background level: (-0.184)
Measured intensity
intensity ((m sr)⁻¹)
q (nm⁻¹)
1000
100
10
1
Range 1.06196e-09 to 1.71426e-08, vol-weighted
totalValue: 2.765e-04 ± 1.035e-06
mean: 3.198e-09 ± 6.727e-12
variance: 5.043e-19 ± 1.790e-20
skew: 5.766e-01 ± 2.092e-01
kurtosis: 4.105e+00 ± 1.034e+00
Sphere radius
Minimum visibility limit
MC size histogram
[Rel.] Volume Fraction
radius (nm)
4e-05
3.5e-05
3e-05
2.5e-05
2e-05
1.5e-05
1e-05
5e-06
0
10
Range 1.06196e-09 to 1.71426e-08, num-weighted
totalValue: 1.000e+00 ± 6.986e-16
mean: 2.672e-09 ± 3.673e-11
variance: 5.232e-19 ± 4.817e-20
skew: 4.605e-02 ± 1.124e-01
kurtosis: 3.451e+00 ± 2.422e-01
Sphere radius
Minimum visibility limit
MC size histogram
[Rel.] Number Fraction
radius (nm)
0.14
0.12
0.1
0.08
0.06
0.04
0.02
0
1
10
