# Supplementary material for: Nanoparticle size distribution quantification: results of a small-angle X-ray scattering inter-laboratory comparison
Source: J Appl Crystallogr. 2017 Aug 18;50(Pt 5):1280–8. doi: 10.1107/S160057671701010X (PMC5627679; doi:10.1107/S160057671701010X)
Supplement: Supplementary file 3 [file j-50-01280-sup2.zip › RRAnonData/csv/S16_2016-12-02_21-08-43/S16_2016-12-02_21-08-43.pdf]

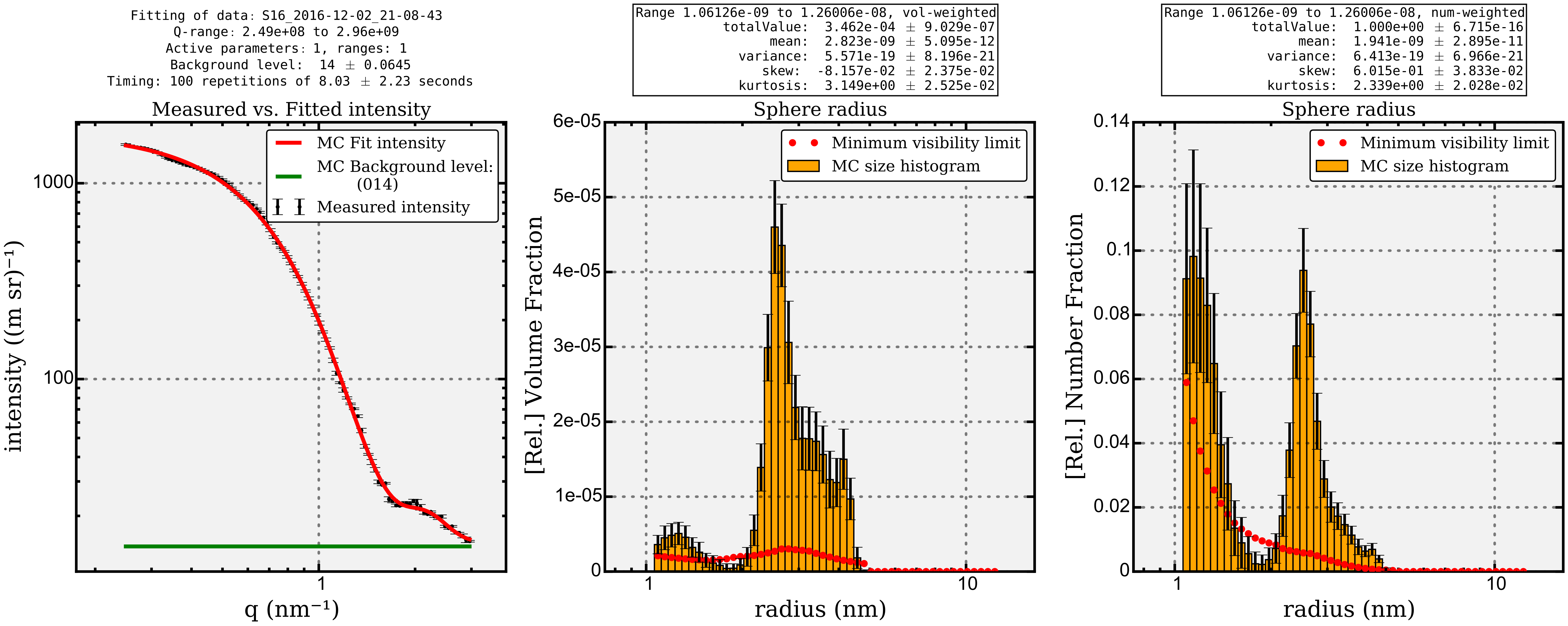

Fitting of data: S16_2016-12-02_21-08-43
Q-range: 2.49e+08 to 2.96e+09
Active parameters: 1, ranges: 1
Background level: 14 ± 0.0645
Timing: 100 repetitions of 8.03 ± 2.23 seconds
Measured vs. Fitted intensity
MC Fit intensity
MC Background level: (014)
Measured intensity
intensity ((m sr)^-1)
q (nm^-1)
Range 1.06126e-09 to 1.26006e-08, vol-weighted
totalValue: 3.462e-04 ± 9.029e-07
mean: 2.823e-09 ± 5.095e-12
variance: 5.571e-19 ± 8.196e-21
skew: -8.157e-02 ± 2.375e-02
kurtosis: 3.149e+00 ± 2.525e-02
Sphere radius
Minimum visibility limit
MC size histogram
[Rel.] Volume Fraction
radius (nm)
Range 1.06126e-09 to 1.26006e-08, num-weighted
totalValue: 1.000e+00 ± 6.715e-16
mean: 1.941e-09 ± 2.895e-11
variance: 6.413e-19 ± 6.966e-21
skew: 6.015e-01 ± 3.833e-02
kurtosis: 2.339e+00 ± 2.028e-02
Sphere radius
Minimum visibility limit
MC size histogram
[Rel.] Number Fraction
radius (nm)
