# Supplementary material for: Nanoparticle size distribution quantification: results of a small-angle X-ray scattering inter-laboratory comparison
Source: J Appl Crystallogr. 2017 Aug 18;50(Pt 5):1280–8. doi: 10.1107/S160057671701010X (PMC5627679; doi:10.1107/S160057671701010X)
Supplement: Supplementary file 3 [file j-50-01280-sup2.zip › RRAnonData/csv/S17_2016-12-02_21-09-37/S17_2016-12-02_21-09-37.pdf]

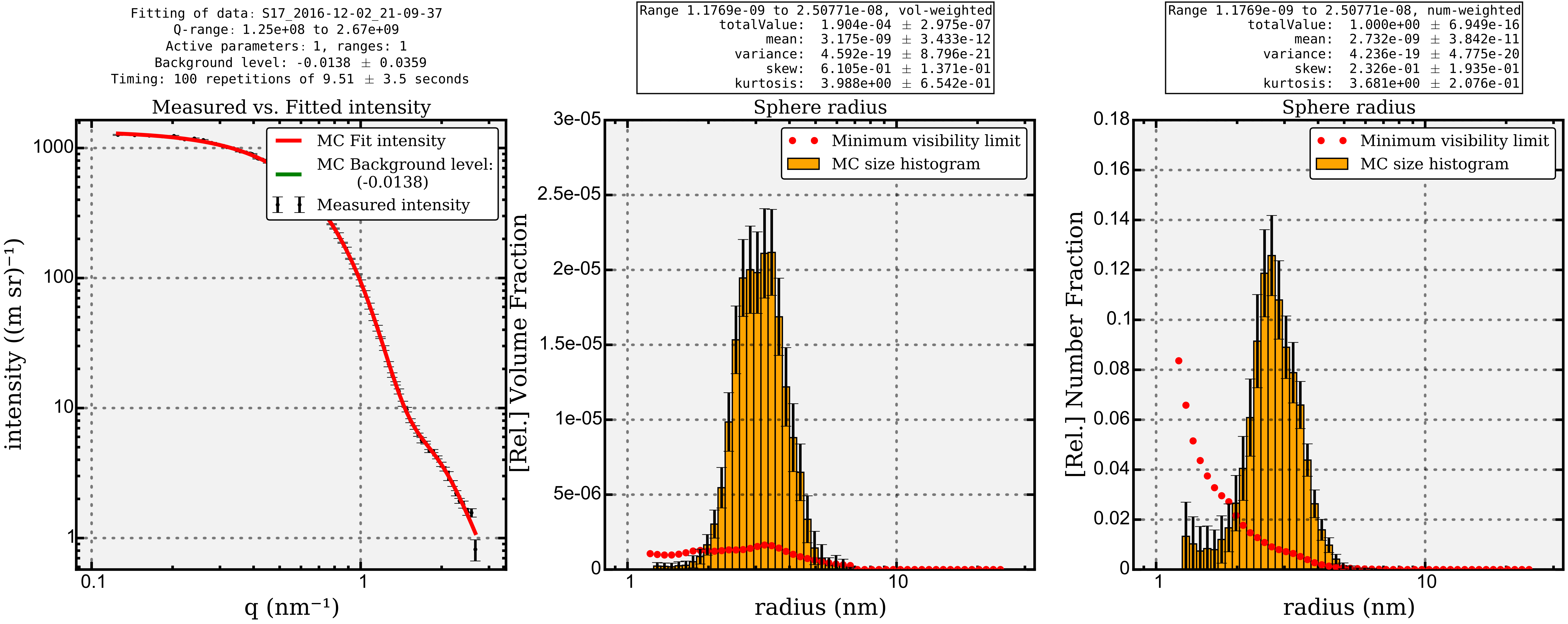
Fitting of data: S17_2016-12-02_21-09-37
Q-range: 1.25e+08 to 2.67e+09
Active parameters: 1, ranges: 1
Background level: -0.0138 ± 0.0359
Timing: 100 repetitions of 9.51 ± 3.5 seconds
Measured vs. Fitted intensity
MC Fit intensity
MC Background level: (-0.0138)
Measured intensity
intensity ((m sr)⁻¹)
q (nm⁻¹)
Range 1.1769e-09 to 2.50771e-08, vol-weighted
totalValue: 1.904e-04 ± 2.975e-07
mean: 3.175e-09 ± 3.433e-12
variance: 4.592e-19 ± 8.796e-21
skew: 6.105e-01 ± 1.371e-01
kurtosis: 3.988e+00 ± 6.542e-01
Sphere radius
Minimum visibility limit
MC size histogram
[Rel.] Volume Fraction
radius (nm)
Range 1.1769e-09 to 2.50771e-08, num-weighted
totalValue: 1.000e+00 ± 6.949e-16
mean: 2.732e-09 ± 3.842e-11
variance: 4.236e-19 ± 4.775e-20
skew: 2.326e-01 ± 1.935e-01
kurtosis: 3.681e+00 ± 2.076e-01
Sphere radius
Minimum visibility limit
MC size histogram
[Rel.] Number Fraction
radius (nm)
