# Supplementary material for: Nanoparticle size distribution quantification: results of a small-angle X-ray scattering inter-laboratory comparison
Source: J Appl Crystallogr. 2017 Aug 18;50(Pt 5):1280–8. doi: 10.1107/S160057671701010X (PMC5627679; doi:10.1107/S160057671701010X)
Supplement: Supplementary file 3 [file j-50-01280-sup2.zip › RRAnonData/csv/S18_2016-12-02_21-25-48/S18_2016-12-02_21-25-48.pdf]

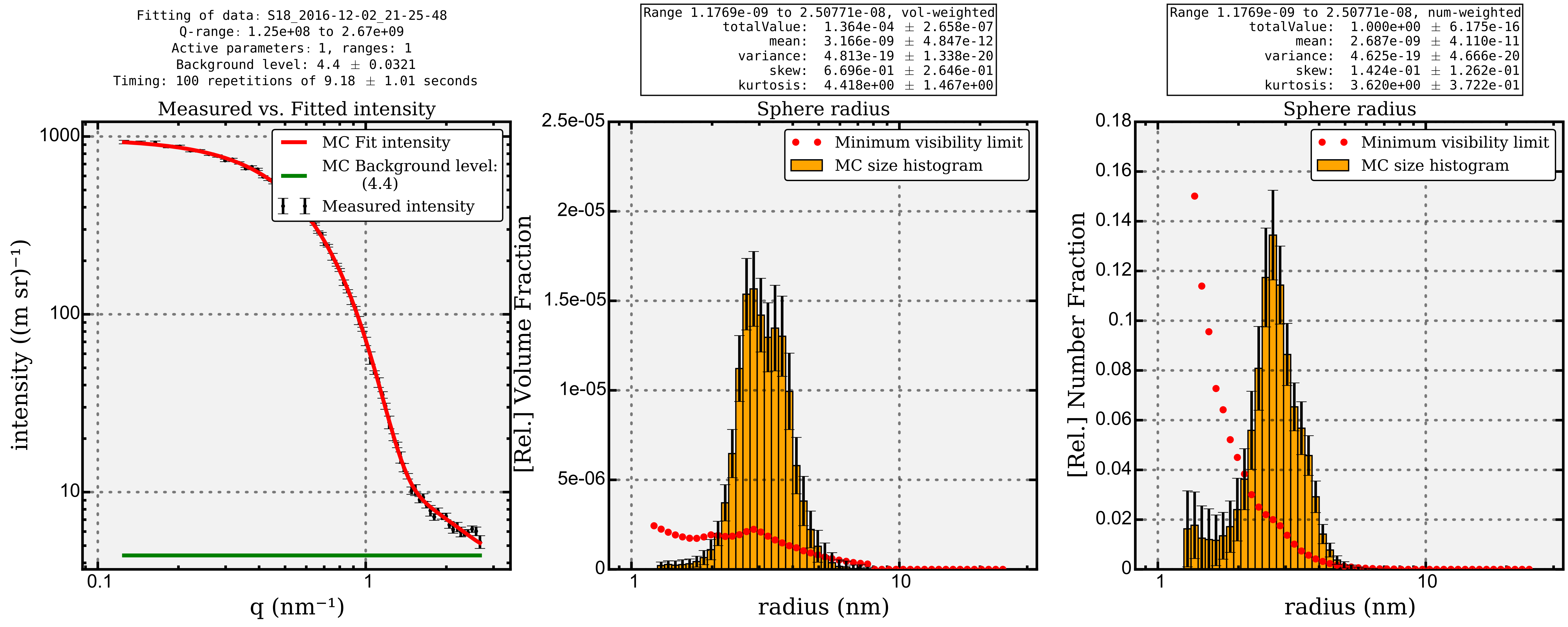
Fitting of data: S18_2016-12-02_21-25-48
Q-range: 1.25e+08 to 2.67e+09
Active parameters: 1, ranges: 1
Background level: 4.4 ± 0.0321
Timing: 100 repetitions of 9.18 ± 1.01 seconds
Measured vs. Fitted intensity
MC Fit intensity
MC Background level: (4.4)
Measured intensity
intensity ((m sr)⁻¹)
q (nm⁻¹)
1000
100
10
0.1
1
Range 1.1769e-09 to 2.50771e-08, vol-weighted
totalValue: 1.364e-04 ± 2.658e-07
mean: 3.166e-09 ± 4.847e-12
variance: 4.813e-19 ± 1.338e-20
skew: 6.696e-01 ± 2.646e-01
kurtosis: 4.418e+00 ± 1.467e+00
Sphere radius
Minimum visibility limit
MC size histogram
[Rel.] Volume Fraction
radius (nm)
2.5e-05
2e-05
1.5e-05
1e-05
5e-06
0
1
10
Range 1.1769e-09 to 2.50771e-08, num-weighted
totalValue: 1.000e+00 ± 6.175e-16
mean: 2.687e-09 ± 4.110e-11
variance: 4.625e-19 ± 4.666e-20
skew: 1.424e-01 ± 1.262e-01
kurtosis: 3.620e+00 ± 3.722e-01
Sphere radius
Minimum visibility limit
MC size histogram
[Rel.] Number Fraction
radius (nm)
0.18
0.16
0.14
0.12
0.1
0.08
0.06
0.04
0.02
0
1
10
