# Supplementary material for: Nanoparticle size distribution quantification: results of a small-angle X-ray scattering inter-laboratory comparison
Source: J Appl Crystallogr. 2017 Aug 18;50(Pt 5):1280–8. doi: 10.1107/S160057671701010X (PMC5627679; doi:10.1107/S160057671701010X)
Supplement: Supplementary file 3 [file j-50-01280-sup2.zip › RRAnonData/csv/S20_2016-12-02_21-22-40/S20_2016-12-02_21-22-40.pdf]

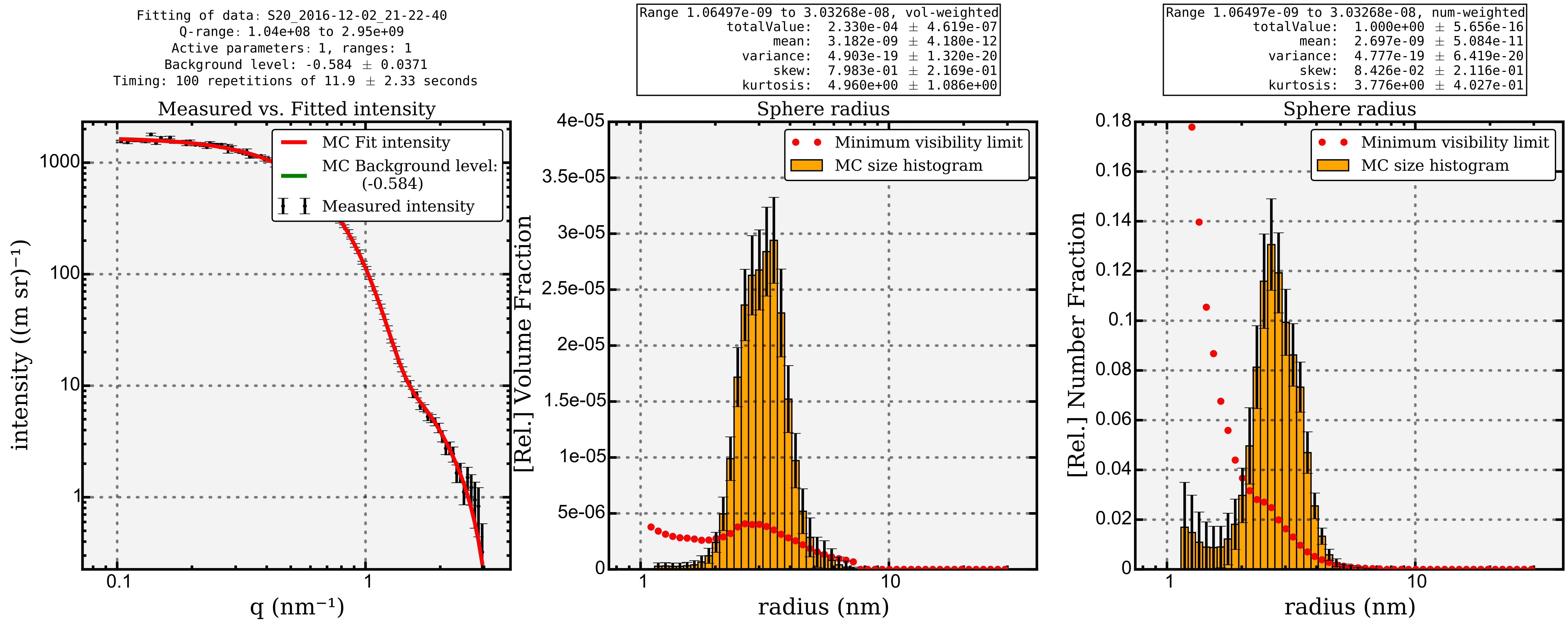
Fitting of data: S20_2016-12-02_21-22-40
Q-range: 1.04e+08 to 2.95e+09
Active parameters: 1, ranges: 1
Background level: -0.584 ± 0.0371
Timing: 100 repetitions of 11.9 ± 2.33 seconds
Measured vs. Fitted intensity
MC Fit intensity
MC Background level: (-0.584)
Measured intensity
intensity ((m sr)$^{-1}$)
1000
100
10
1
0.1
1
q (nm^{-1})
Range 1.06497e-09 to 3.03268e-08, vol-weighted
totalValue: 2.330e-04 ± 4.619e-07
mean: 3.182e-09 ± 4.180e-12
variance: 4.903e-19 ± 1.320e-20
skew: 7.983e-01 ± 2.169e-01
kurtosis: 4.960e+00 ± 1.086e+00
Sphere radius
Minimum visibility limit
MC size histogram
[Rel.] Volume Fraction
4e-05
3.5e-05
3e-05
2.5e-05
2e-05
1.5e-05
1e-05
5e-06
0
1
10
radius (nm)
Range 1.06497e-09 to 3.03268e-08, num-weighted
totalValue: 1.000e+00 ± 5.656e-16
mean: 2.697e-09 ± 5.084e-11
variance: 4.777e-19 ± 6.419e-20
skew: 8.426e-02 ± 2.116e-01
kurtosis: 3.776e+00 ± 4.027e-01
Sphere radius
Minimum visibility limit
MC size histogram
[Rel.] Number Fraction
0.18
0.16
0.14
0.12
0.1
0.08
0.06
0.04
0.02
0
1
10
radius (nm)
