# Supplementary material for: Nanoparticle size distribution quantification: results of a small-angle X-ray scattering inter-laboratory comparison
Source: J Appl Crystallogr. 2017 Aug 18;50(Pt 5):1280–8. doi: 10.1107/S160057671701010X (PMC5627679; doi:10.1107/S160057671701010X)
Supplement: Supplementary file 3 [file j-50-01280-sup2.zip › RRAnonData/csv/S22_2016-12-02_21-23-25/S22_2016-12-02_21-23-25.pdf]

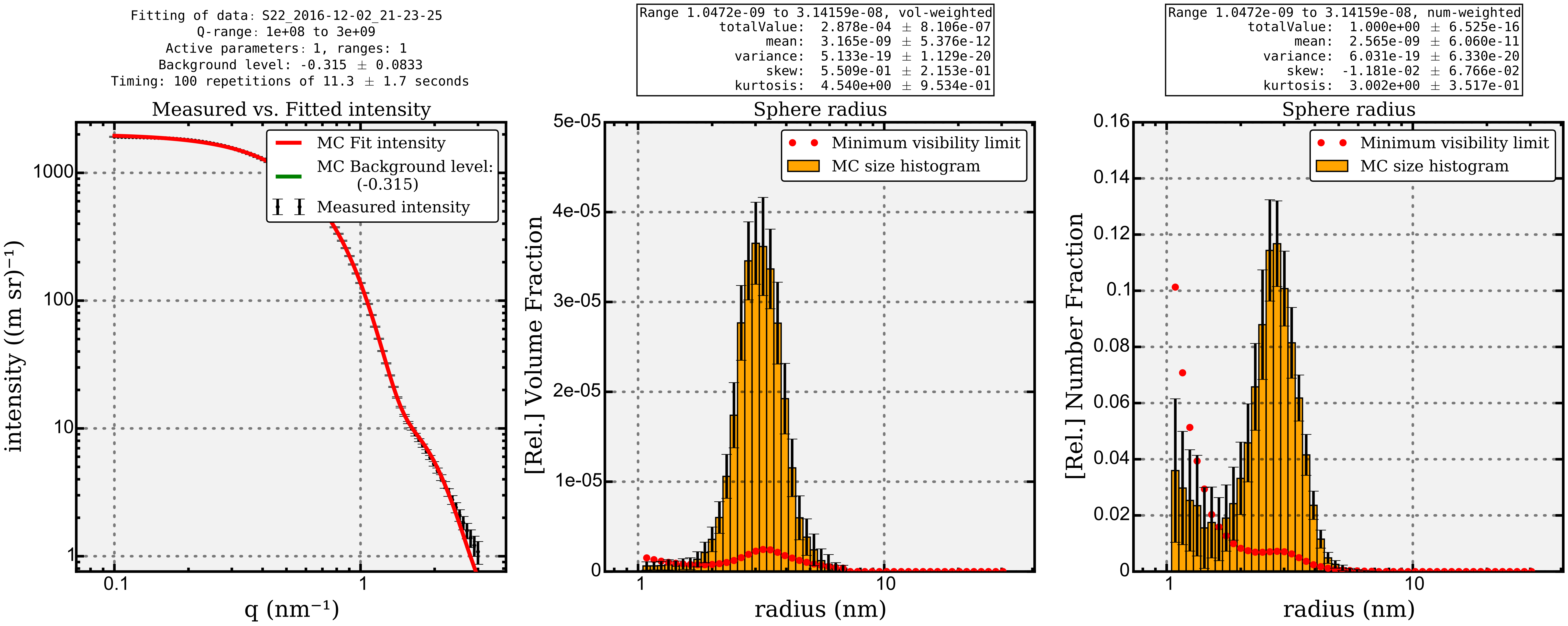

Fitting of data: S22_2016-12-02_21-23-25
Q-range: 1e+08 to 3e+09
Active parameters: 1, ranges: 1
Background level: -0.315 ± 0.0833
Timing: 100 repetitions of 11.3 ± 1.7 seconds
Measured vs. Fitted intensity
MC Fit intensity
MC Background level: (-0.315)
Measured intensity
intensity ((m sr)$^{-1}$)
q (nm^{-1})
Range 1.0472e-09 to 3.14159e-08, vol-weighted
totalValue: 2.878e-04 ± 8.106e-07
mean: 3.165e-09 ± 5.376e-12
variance: 5.133e-19 ± 1.129e-20
skew: 5.509e-01 ± 2.153e-01
kurtosis: 4.540e+00 ± 9.534e-01
Sphere radius
Minimum visibility limit
MC size histogram
[Rel.] Volume Fraction
radius (nm)
Range 1.0472e-09 to 3.14159e-08, num-weighted
totalValue: 1.000e+00 ± 6.525e-16
mean: 2.565e-09 ± 6.060e-11
variance: 6.031e-19 ± 6.330e-20
skew: -1.181e-02 ± 6.766e-02
kurtosis: 3.002e+00 ± 3.517e-01
Sphere radius
Minimum visibility limit
MC size histogram
[Rel.] Number Fraction
radius (nm)
