# Supplementary material for: Nanoparticle size distribution quantification: results of a small-angle X-ray scattering inter-laboratory comparison
Source: J Appl Crystallogr. 2017 Aug 18;50(Pt 5):1280–8. doi: 10.1107/S160057671701010X (PMC5627679; doi:10.1107/S160057671701010X)
Supplement: Supplementary file 3 [file j-50-01280-sup2.zip › RRAnonData/csv/S23_2016-12-02_21-44-07/S23_2016-12-02_21-44-07.pdf]

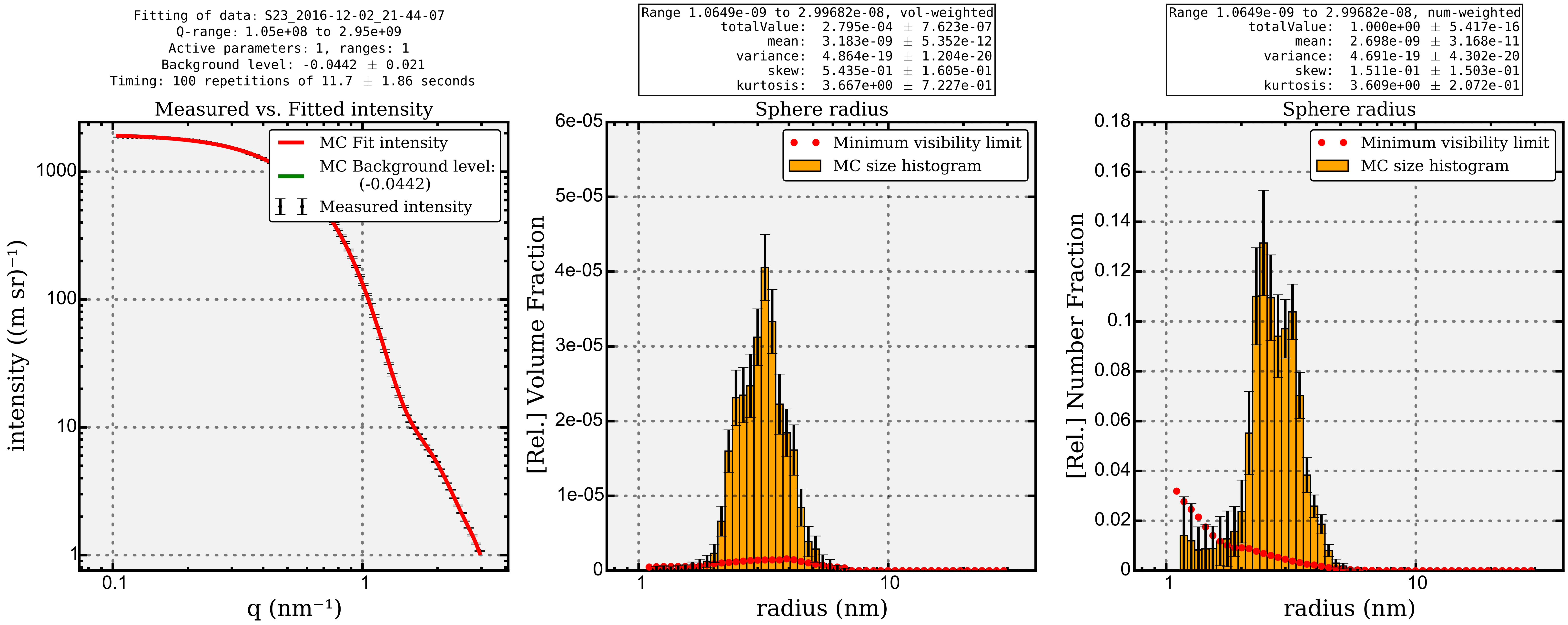
Fitting of data: S23_2016-12-02_21-44-07
Q-range: 1.05e+08 to 2.95e+09
Active parameters: 1, ranges: 1
Background level: -0.0442 ± 0.021
Timing: 100 repetitions of 11.7 ± 1.86 seconds
Measured vs. Fitted intensity
MC Fit intensity
MC Background level: (-0.0442)
Measured intensity
intensity ((m sr)⁻¹)
q (nm⁻¹)
1000
100
10
1
0.1
1
Range 1.0649e-09 to 2.99682e-08, vol-weighted
totalValue: 2.795e-04 ± 7.623e-07
mean: 3.183e-09 ± 5.352e-12
variance: 4.864e-19 ± 1.204e-20
skew: 5.435e-01 ± 1.605e-01
kurtosis: 3.667e+00 ± 7.227e-01
Sphere radius
Minimum visibility limit
MC size histogram
[Rel.] Volume Fraction
radius (nm)
6e-05
5e-05
4e-05
3e-05
2e-05
1e-05
0
1
10
Range 1.0649e-09 to 2.99682e-08, num-weighted
totalValue: 1.000e+00 ± 5.417e-16
mean: 2.698e-09 ± 3.168e-11
variance: 4.691e-19 ± 4.302e-20
skew: 1.511e-01 ± 1.503e-01
kurtosis: 3.609e+00 ± 2.072e-01
Sphere radius
Minimum visibility limit
MC size histogram
[Rel.] Number Fraction
radius (nm)
0.18
0.16
0.14
0.12
0.1
0.08
0.06
0.04
0.02
0
1
10
