# Supplementary material for: Nanoparticle size distribution quantification: results of a small-angle X-ray scattering inter-laboratory comparison
Source: J Appl Crystallogr. 2017 Aug 18;50(Pt 5):1280–8. doi: 10.1107/S160057671701010X (PMC5627679; doi:10.1107/S160057671701010X)
Supplement: Supplementary file 3 [file j-50-01280-sup2.zip › RRAnonData/csv/S24_2016-12-02_22-03-45/S24_2016-12-02_22-03-45.pdf]

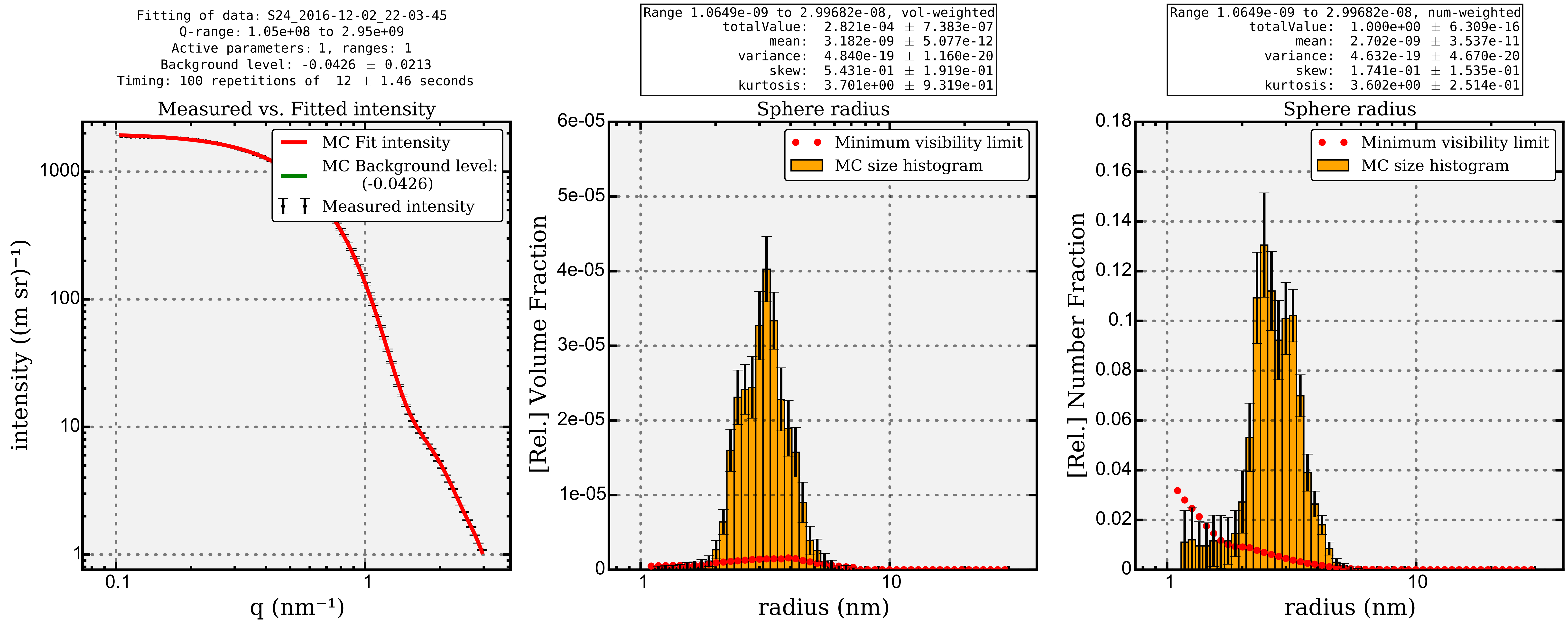
Fitting of data: S24_2016-12-02_22-03-45
Q-range: 1.05e+08 to 2.95e+09
Active parameters: 1, ranges: 1
Background level: -0.0426 ± 0.0213
Timing: 100 repetitions of 12 ± 1.46 seconds
Measured vs. Fitted intensity
MC Fit intensity
MC Background level: (-0.0426)
Measured intensity
intensity ((m sr)$^{-1}$)
q (nm^{-1})
1000
100
10
1
0.1
1
Range 1.0649e-09 to 2.99682e-08, vol-weighted
totalValue: 2.821e-04 ± 7.383e-07
mean: 3.182e-09 ± 5.077e-12
variance: 4.840e-19 ± 1.160e-20
skew: 5.431e-01 ± 1.919e-01
kurtosis: 3.701e+00 ± 9.319e-01
Sphere radius
Minimum visibility limit
MC size histogram
[Rel.] Volume Fraction
radius (nm)
6e-05
5e-05
4e-05
3e-05
2e-05
1e-05
0
1
10
Range 1.0649e-09 to 2.99682e-08, num-weighted
totalValue: 1.000e+00 ± 6.309e-16
mean: 2.702e-09 ± 3.537e-11
variance: 4.632e-19 ± 4.670e-20
skew: 1.741e-01 ± 1.535e-01
kurtosis: 3.602e+00 ± 2.514e-01
Sphere radius
Minimum visibility limit
MC size histogram
[Rel.] Number Fraction
radius (nm)
0.18
0.16
0.14
0.12
0.1
0.08
0.06
0.04
0.02
0
1
10
