# Supplementary material for: Nanoparticle size distribution quantification: results of a small-angle X-ray scattering inter-laboratory comparison
Source: J Appl Crystallogr. 2017 Aug 18;50(Pt 5):1280–8. doi: 10.1107/S160057671701010X (PMC5627679; doi:10.1107/S160057671701010X)
Supplement: Supplementary file 3 [file j-50-01280-sup2.zip › RRAnonData/csv/S25_2016-12-02_22-02-33/S25_2016-12-02_22-02-33.pdf]

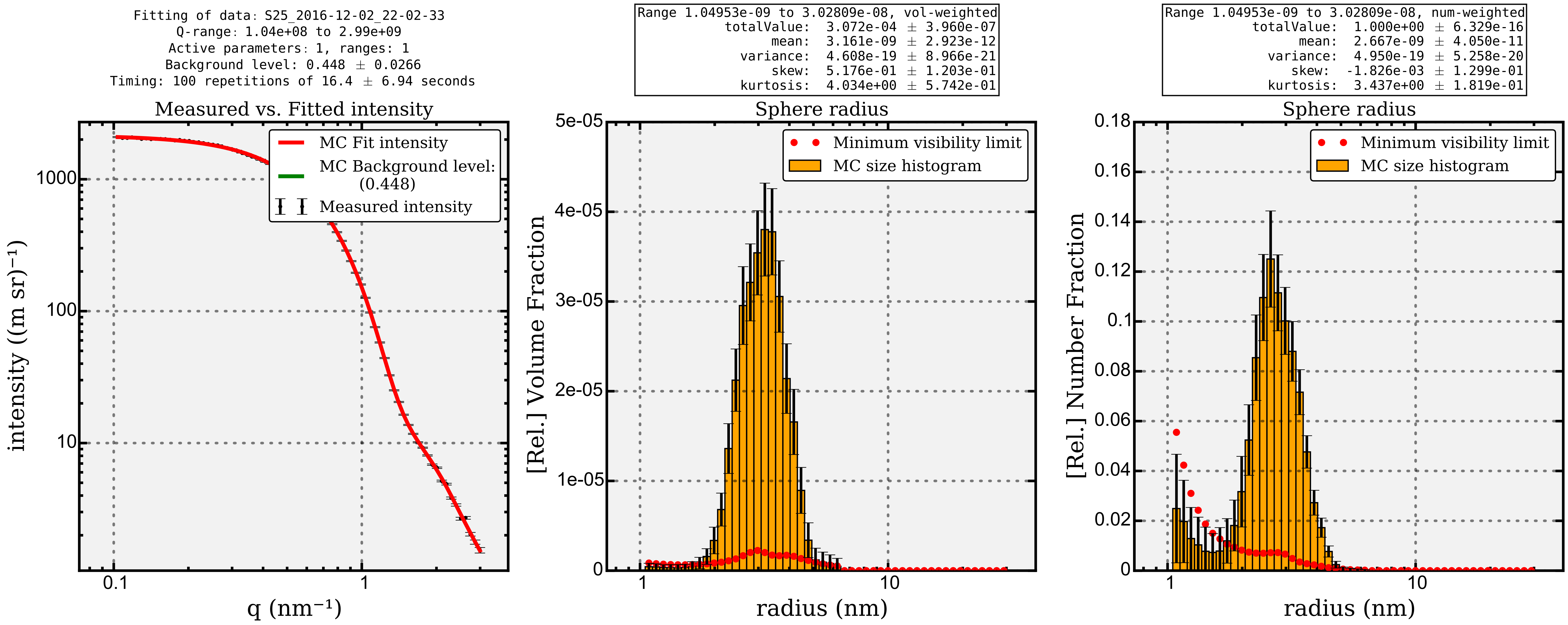

Fitting of data: S25_2016-12-02_22-02-33
Q-range: 1.04e+08 to 2.99e+09
Active parameters: 1, ranges: 1
Background level: 0.448 ± 0.0266
Timing: 100 repetitions of 16.4 ± 6.94 seconds
Measured vs. Fitted intensity
MC Fit intensity
MC Background level: (0.448)
Measured intensity
intensity ((m sr)$^{-1}$)
1000
100
10
0.1
1
q (nm^{-1})
Range 1.04953e-09 to 3.02809e-08, vol-weighted
totalValue: 3.072e-04 ± 3.960e-07
mean: 3.161e-09 ± 2.923e-12
variance: 4.608e-19 ± 8.966e-21
skew: 5.176e-01 ± 1.203e-01
kurtosis: 4.034e+00 ± 5.742e-01
Sphere radius
Minimum visibility limit
MC size histogram
[Rel.] Volume Fraction
5e-05
4e-05
3e-05
2e-05
1e-05
0
1
10
radius (nm)
Range 1.04953e-09 to 3.02809e-08, num-weighted
totalValue: 1.000e+00 ± 6.329e-16
mean: 2.667e-09 ± 4.050e-11
variance: 4.950e-19 ± 5.258e-20
skew: -1.826e-03 ± 1.299e-01
kurtosis: 3.437e+00 ± 1.819e-01
Sphere radius
Minimum visibility limit
MC size histogram
[Rel.] Number Fraction
0.18
0.16
0.14
0.12
0.1
0.08
0.06
0.04
0.02
0
1
10
radius (nm)
