# Supplementary material for: Nanoparticle size distribution quantification: results of a small-angle X-ray scattering inter-laboratory comparison
Source: J Appl Crystallogr. 2017 Aug 18;50(Pt 5):1280–8. doi: 10.1107/S160057671701010X (PMC5627679; doi:10.1107/S160057671701010X)
Supplement: Supplementary file 3 [file j-50-01280-sup2.zip › RRAnonData/csv/S27_2016-12-02_22-04-19/S27_2016-12-02_22-04-19.pdf]

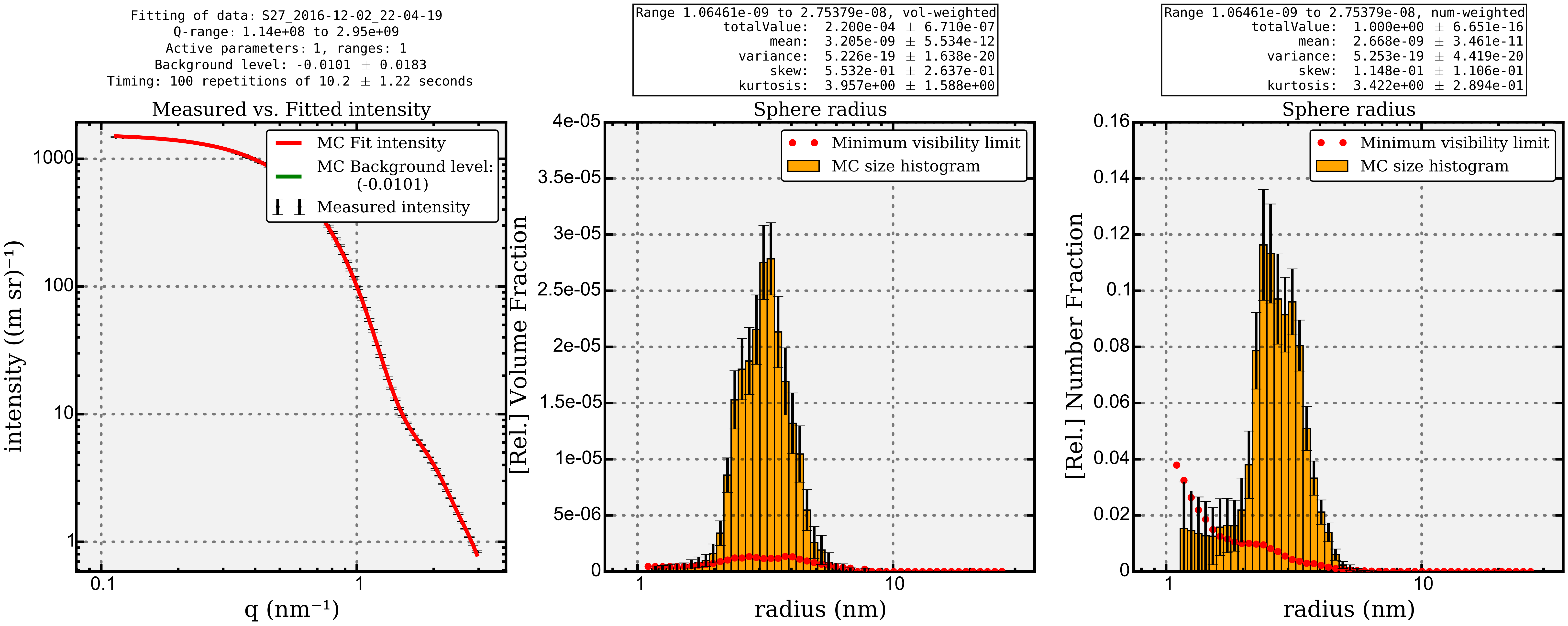

Fitting of data: S27_2016-12-02_22-04-19
Q-range: 1.14e+08 to 2.95e+09
Active parameters: 1, ranges: 1
Background level: -0.0101 ± 0.0183
Timing: 100 repetitions of 10.2 ± 1.22 seconds
Measured vs. Fitted intensity
MC Fit intensity
MC Background level: (-0.0101)
Measured intensity
intensity ((m sr)⁻¹)
q (nm⁻¹)
Range 1.06461e-09 to 2.75379e-08, vol-weighted
totalValue: 2.200e-04 ± 6.710e-07
mean: 3.205e-09 ± 5.534e-12
variance: 5.226e-19 ± 1.638e-20
skew: 5.532e-01 ± 2.637e-01
kurtosis: 3.957e+00 ± 1.588e+00
Sphere radius
Minimum visibility limit
MC size histogram
[Rel.] Volume Fraction
radius (nm)
Range 1.06461e-09 to 2.75379e-08, num-weighted
totalValue: 1.000e+00 ± 6.651e-16
mean: 2.668e-09 ± 3.461e-11
variance: 5.253e-19 ± 4.419e-20
skew: 1.148e-01 ± 1.106e-01
kurtosis: 3.422e+00 ± 2.894e-01
Sphere radius
Minimum visibility limit
MC size histogram
[Rel.] Number Fraction
radius (nm)
