# Supplementary material for: Nanoparticle size distribution quantification: results of a small-angle X-ray scattering inter-laboratory comparison
Source: J Appl Crystallogr. 2017 Aug 18;50(Pt 5):1280–8. doi: 10.1107/S160057671701010X (PMC5627679; doi:10.1107/S160057671701010X)
Supplement: Supplementary file 3 [file j-50-01280-sup2.zip › RRAnonData/csv/S28_2016-12-02_22-26-12/S28_2016-12-02_22-26-12.pdf]

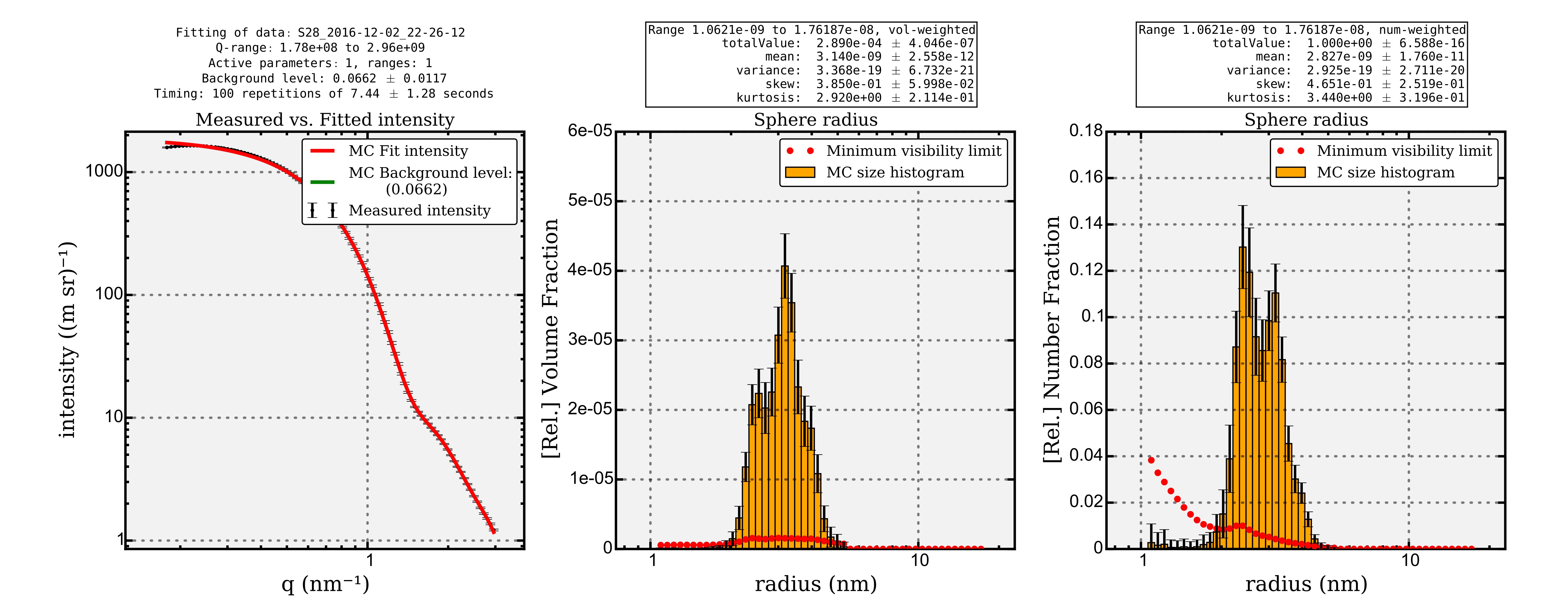

Fitting of data: S28_2016-12-02_22-26-12
Q-range: 1.78e+08 to 2.96e+09
Active parameters: 1, ranges: 1
Background level: 0.0662 ± 0.0117
Timing: 100 repetitions of 7.44 ± 1.28 seconds
Measured vs. Fitted intensity
MC Fit intensity
MC Background level: (0.0662)
Measured intensity
intensity ((m sr)$^{-1}$)
q (nm^{-1})
1000
100
10
1
Range 1.0621e-09 to 1.76187e-08, vol-weighted
totalValue: 2.890e-04 ± 4.046e-07
mean: 3.140e-09 ± 2.558e-12
variance: 3.368e-19 ± 6.732e-21
skew: 3.850e-01 ± 5.998e-02
kurtosis: 2.920e+00 ± 2.114e-01
Sphere radius
Minimum visibility limit
MC size histogram
[Rel.] Volume Fraction
6e-05
5e-05
4e-05
3e-05
2e-05
1e-05
0
1
10
radius (nm)
Range 1.0621e-09 to 1.76187e-08, num-weighted
totalValue: 1.000e+00 ± 6.588e-16
mean: 2.827e-09 ± 1.760e-11
variance: 2.925e-19 ± 2.711e-20
skew: 4.651e-01 ± 2.519e-01
kurtosis: 3.440e+00 ± 3.196e-01
Sphere radius
Minimum visibility limit
MC size histogram
[Rel.] Number Fraction
0.18
0.16
0.14
0.12
0.1
0.08
0.06
0.04
0.02
0
1
10
radius (nm)
