# Supplementary material for: Nanoparticle size distribution quantification: results of a small-angle X-ray scattering inter-laboratory comparison
Source: J Appl Crystallogr. 2017 Aug 18;50(Pt 5):1280–8. doi: 10.1107/S160057671701010X (PMC5627679; doi:10.1107/S160057671701010X)
Supplement: Supplementary file 3 [file j-50-01280-sup2.zip › RRAnonData/csv/S29_2016-12-02_21-42-29/S29_2016-12-02_21-42-29.pdf]

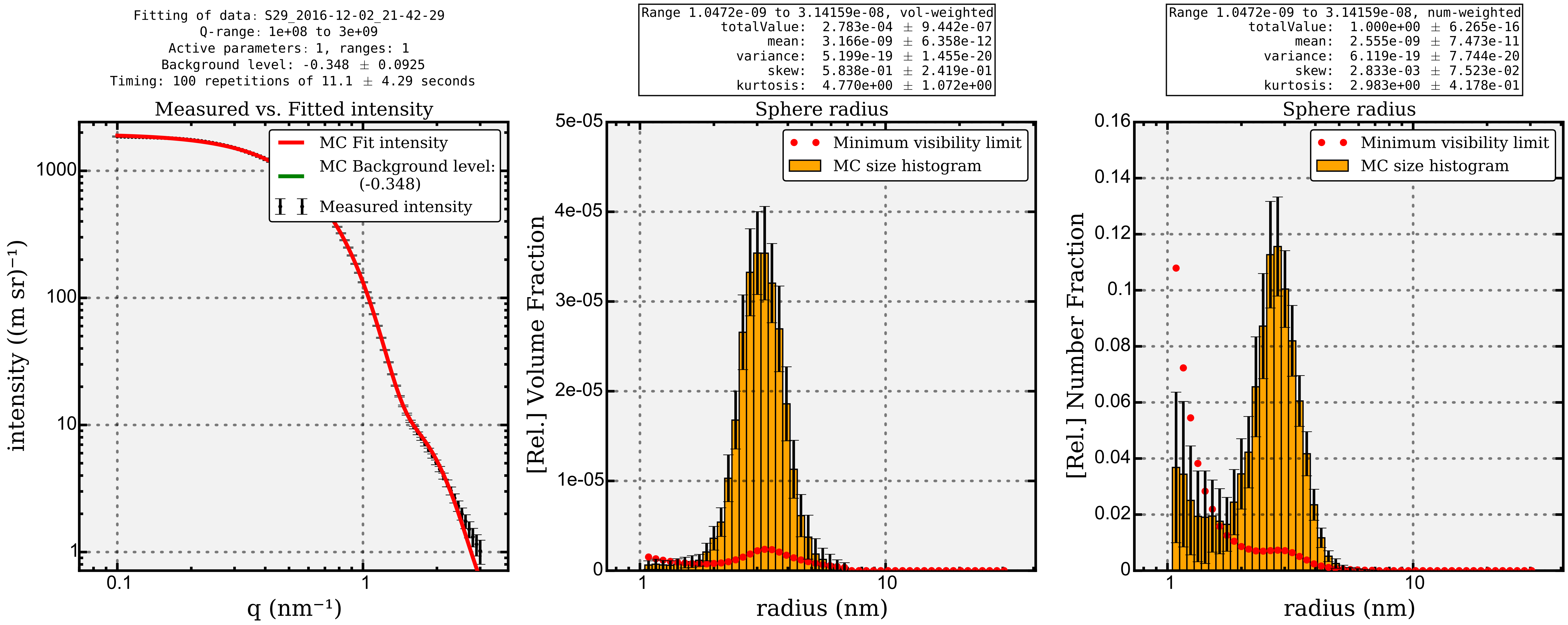

Fitting of data: S29_2016-12-02_21-42-29
Q-range: 1e+08 to 3e+09
Active parameters: 1, ranges: 1
Background level: -0.348 ± 0.0925
Timing: 100 repetitions of 11.1 ± 4.29 seconds
Measured vs. Fitted intensity
MC Fit intensity
MC Background level: (-0.348)
Measured intensity
intensity ((m sr)⁻¹)
1000
100
10
1
0.1
1
q (nm⁻¹)
Range 1.0472e-09 to 3.14159e-08, vol-weighted
totalValue: 2.783e-04 ± 9.442e-07
mean: 3.166e-09 ± 6.358e-12
variance: 5.199e-19 ± 1.455e-20
skew: 5.838e-01 ± 2.419e-01
kurtosis: 4.770e+00 ± 1.072e+00
Sphere radius
Minimum visibility limit
MC size histogram
[Rel.] Volume Fraction
5e-05
4e-05
3e-05
2e-05
1e-05
0
1
10
radius (nm)
Range 1.0472e-09 to 3.14159e-08, num-weighted
totalValue: 1.000e+00 ± 6.265e-16
mean: 2.555e-09 ± 7.473e-11
variance: 6.119e-19 ± 7.744e-20
skew: 2.833e-03 ± 7.523e-02
kurtosis: 2.983e+00 ± 4.178e-01
Sphere radius
Minimum visibility limit
MC size histogram
[Rel.] Number Fraction
0.16
0.14
0.12
0.1
0.08
0.06
0.04
0.02
0
1
10
radius (nm)
