# Supplementary material for: Nanoparticle size distribution quantification: results of a small-angle X-ray scattering inter-laboratory comparison
Source: J Appl Crystallogr. 2017 Aug 18;50(Pt 5):1280–8. doi: 10.1107/S160057671701010X (PMC5627679; doi:10.1107/S160057671701010X)
Supplement: Supplementary file 3 [file j-50-01280-sup2.zip › RRAnonData/csv/S30_2016-12-02_22-31-04/S30_2016-12-02_22-31-04.pdf]

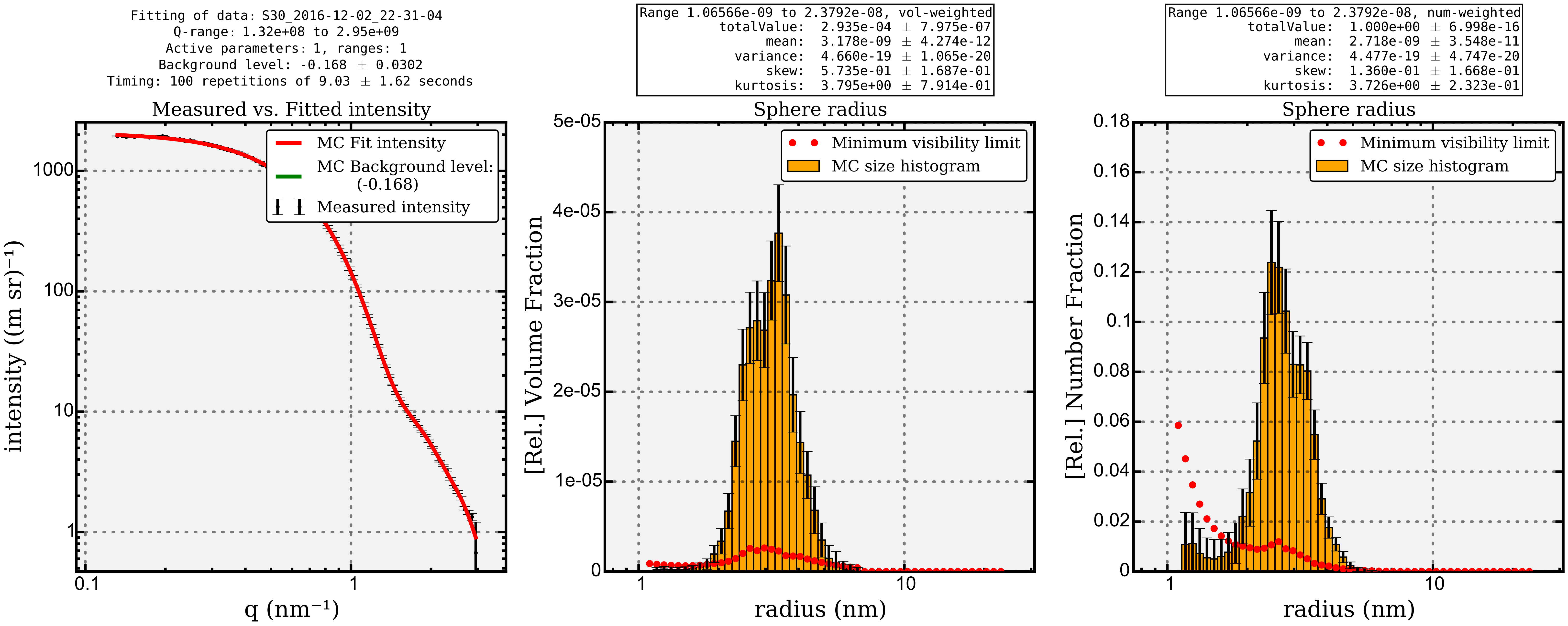
Fitting of data: S30_2016-12-02_22-31-04
Q-range: 1.32e+08 to 2.95e+09
Active parameters: 1, ranges: 1
Background level: -0.168 ± 0.0302
Timing: 100 repetitions of 9.03 ± 1.62 seconds
Measured vs. Fitted intensity
MC Fit intensity
MC Background level: (-0.168)
Measured intensity
intensity ((m sr)$^{-1}$)
q (nm^{-1})
Range 1.06566e-09 to 2.3792e-08, vol-weighted
totalValue: 2.935e-04 ± 7.975e-07
mean: 3.178e-09 ± 4.274e-12
variance: 4.660e-19 ± 1.065e-20
skew: 5.735e-01 ± 1.687e-01
kurtosis: 3.795e+00 ± 7.914e-01
Sphere radius
Minimum visibility limit
MC size histogram
[Rel.] Volume Fraction
radius (nm)
Range 1.06566e-09 to 2.3792e-08, num-weighted
totalValue: 1.000e+00 ± 6.998e-16
mean: 2.718e-09 ± 3.548e-11
variance: 4.477e-19 ± 4.747e-20
skew: 1.360e-01 ± 1.668e-01
kurtosis: 3.726e+00 ± 2.323e-01
Sphere radius
Minimum visibility limit
MC size histogram
[Rel.] Number Fraction
radius (nm)
