# Supplementary material for: Nanoparticle size distribution quantification: results of a small-angle X-ray scattering inter-laboratory comparison
Source: J Appl Crystallogr. 2017 Aug 18;50(Pt 5):1280–8. doi: 10.1107/S160057671701010X (PMC5627679; doi:10.1107/S160057671701010X)
Supplement: Supplementary file 3 [file j-50-01280-sup2.zip › RRAnonData/csv/S31_2016-12-02_22-46-25/S31_2016-12-02_22-46-25.pdf]

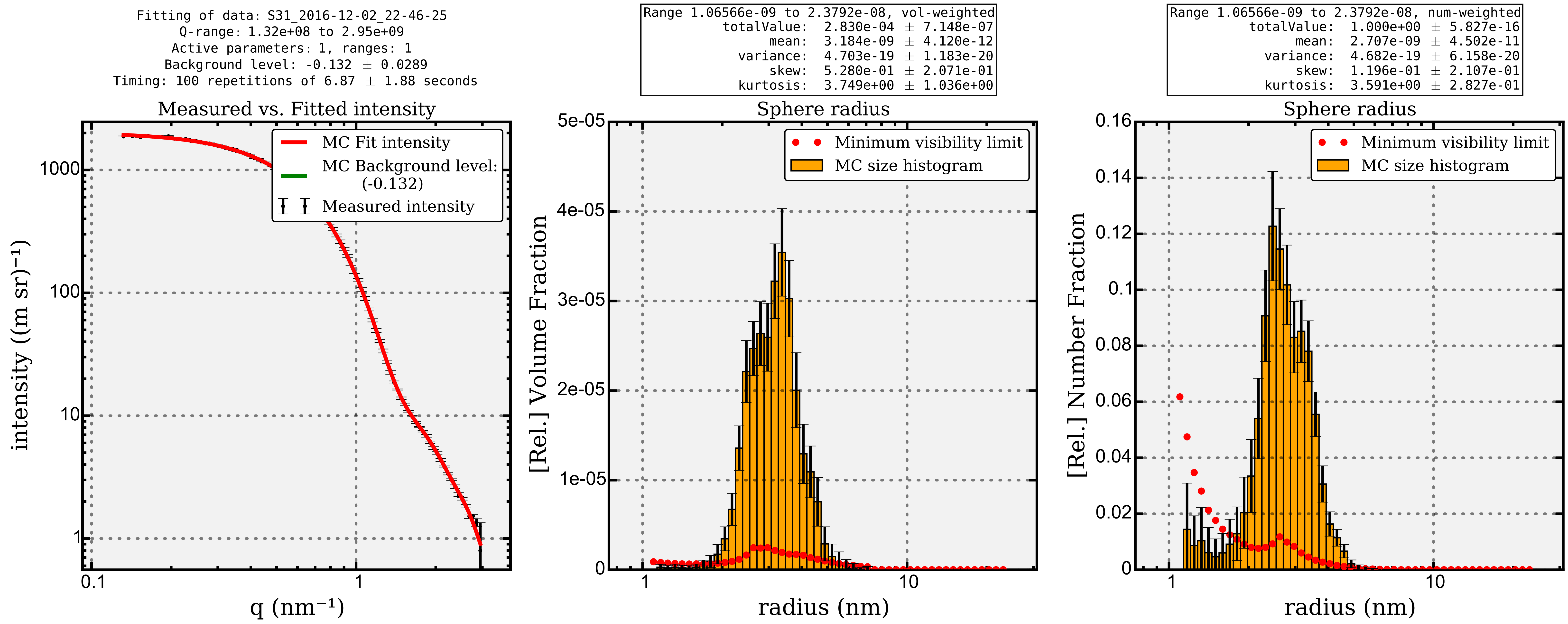

Fitting of data: S31_2016-12-02_22-46-25
Q-range: 1.32e+08 to 2.95e+09
Active parameters: 1, ranges: 1
Background level: -0.132 ± 0.0289
Timing: 100 repetitions of 6.87 ± 1.88 seconds
Measured vs. Fitted intensity
MC Fit intensity
MC Background level: (-0.132)
Measured intensity
intensity ((m sr)⁻¹)
q (nm⁻¹)
Range 1.06566e-09 to 2.3792e-08, vol-weighted
totalValue: 2.830e-04 ± 7.148e-07
mean: 3.184e-09 ± 4.120e-12
variance: 4.703e-19 ± 1.183e-20
skew: 5.280e-01 ± 2.071e-01
kurtosis: 3.749e+00 ± 1.036e+00
Sphere radius
Minimum visibility limit
MC size histogram
[Rel.] Volume Fraction
radius (nm)
Range 1.06566e-09 to 2.3792e-08, num-weighted
totalValue: 1.000e+00 ± 5.827e-16
mean: 2.707e-09 ± 4.502e-11
variance: 4.682e-19 ± 6.158e-20
skew: 1.196e-01 ± 2.107e-01
kurtosis: 3.591e+00 ± 2.827e-01
Sphere radius
Minimum visibility limit
MC size histogram
[Rel.] Number Fraction
radius (nm)
