# Supplementary material for: Nanoparticle size distribution quantification: results of a small-angle X-ray scattering inter-laboratory comparison
Source: J Appl Crystallogr. 2017 Aug 18;50(Pt 5):1280–8. doi: 10.1107/S160057671701010X (PMC5627679; doi:10.1107/S160057671701010X)
Supplement: Supplementary file 3 [file j-50-01280-sup2.zip › RRAnonData/csv/S32_2016-12-02_22-38-53/S32_2016-12-02_22-38-53.pdf]

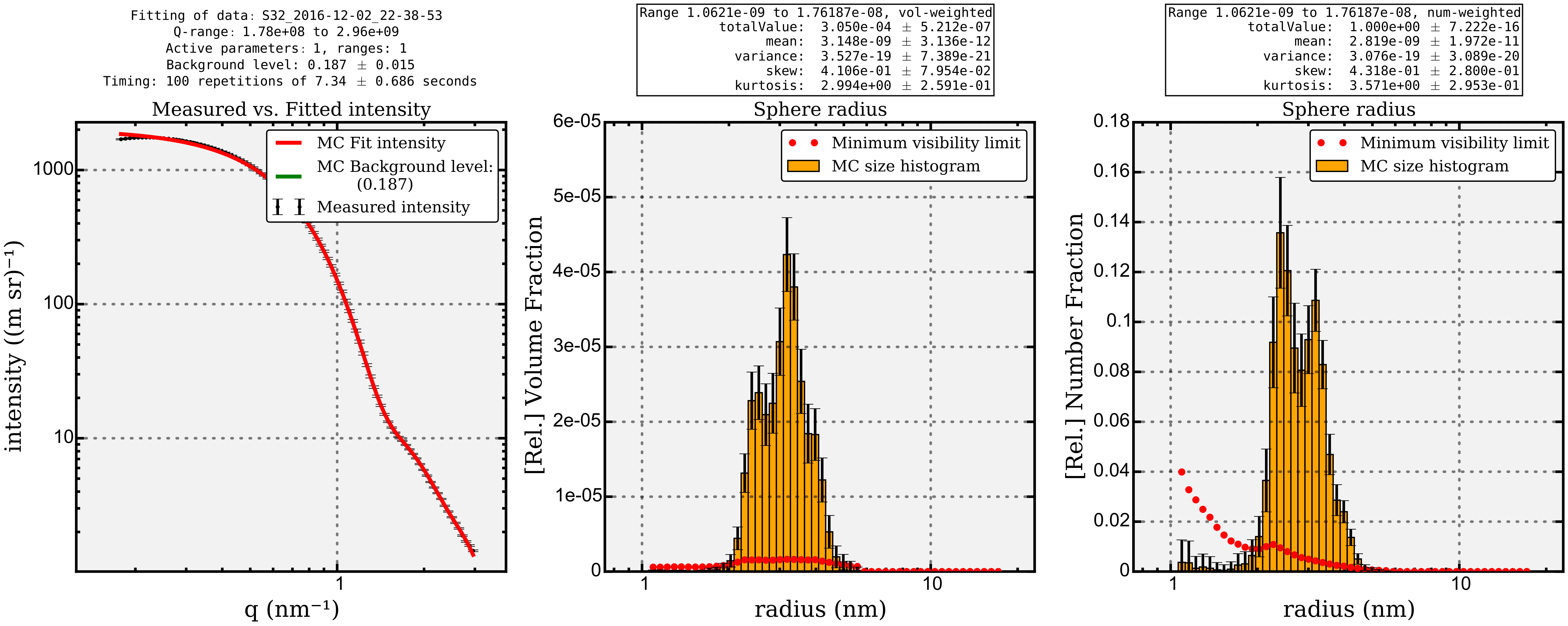

Fitting of data: S32_2016-12-02_22-38-53
Q-range: 1.78e+08 to 2.96e+09
Active parameters: 1, ranges: 1
Background level: 0.187 ± 0.015
Timing: 100 repetitions of 7.34 ± 0.686 seconds
Measured vs. Fitted intensity
MC Fit intensity
MC Background level: (0.187)
Measured intensity
intensity ((m sr)$^{-1}$)
q (nm^{-1})
1000
100
10
1
Range 1.0621e-09 to 1.76187e-08, vol-weighted
totalValue: 3.050e-04 ± 5.212e-07
mean: 3.148e-09 ± 3.136e-12
variance: 3.527e-19 ± 7.389e-21
skew: 4.106e-01 ± 7.954e-02
kurtosis: 2.994e+00 ± 2.591e-01
Sphere radius
Minimum visibility limit
MC size histogram
[Rel.] Volume Fraction
radius (nm)
6e-05
5e-05
4e-05
3e-05
2e-05
1e-05
0
1
10
Range 1.0621e-09 to 1.76187e-08, num-weighted
totalValue: 1.000e+00 ± 7.222e-16
mean: 2.819e-09 ± 1.972e-11
variance: 3.076e-19 ± 3.089e-20
skew: 4.318e-01 ± 2.800e-01
kurtosis: 3.571e+00 ± 2.953e-01
Sphere radius
Minimum visibility limit
MC size histogram
[Rel.] Number Fraction
radius (nm)
0.18
0.16
0.14
0.12
0.1
0.08
0.06
0.04
0.02
0
1
10
