# Supplementary material for: Nanoparticle size distribution quantification: results of a small-angle X-ray scattering inter-laboratory comparison
Source: J Appl Crystallogr. 2017 Aug 18;50(Pt 5):1280–8. doi: 10.1107/S160057671701010X (PMC5627679; doi:10.1107/S160057671701010X)
Supplement: Supplementary file 3 [file j-50-01280-sup2.zip › RRAnonData/csv/S33_2016-12-02_22-21-42/S33_2016-12-02_22-21-42.pdf]

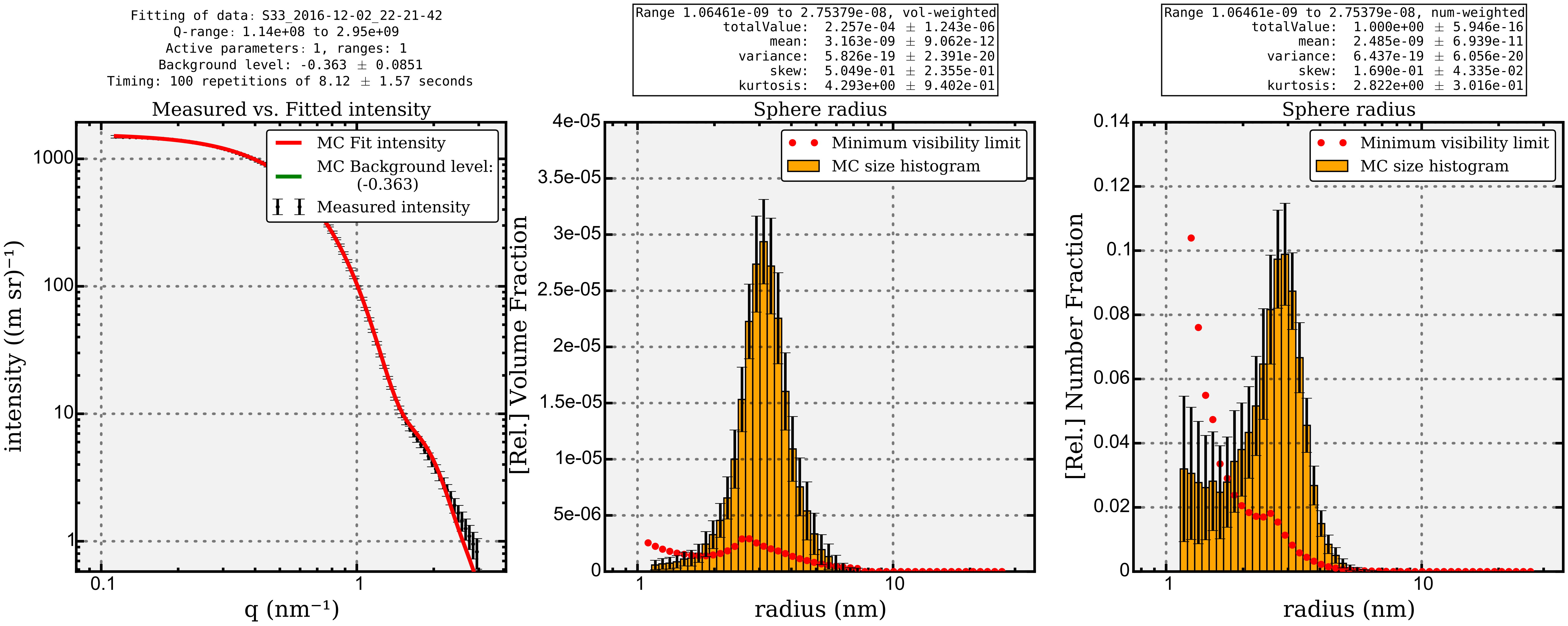
Fitting of data: S33_2016-12-02_22-21-42
Q-range: 1.14e+08 to 2.95e+09
Active parameters: 1, ranges: 1
Background level: -0.363 ± 0.0851
Timing: 100 repetitions of 8.12 ± 1.57 seconds
Measured vs. Fitted intensity
MC Fit intensity
MC Background level: (-0.363)
Measured intensity
intensity ((m sr)$^{-1}$)
1000
100
10
1
0.1
1
q (nm^{-1})
Range 1.06461e-09 to 2.75379e-08, vol-weighted
totalValue: 2.257e-04 ± 1.243e-06
mean: 3.163e-09 ± 9.062e-12
variance: 5.826e-19 ± 2.391e-20
skew: 5.049e-01 ± 2.355e-01
kurtosis: 4.293e+00 ± 9.402e-01
Sphere radius
Minimum visibility limit
MC size histogram
[Rel.] Volume Fraction
4e-05
3.5e-05
3e-05
2.5e-05
2e-05
1.5e-05
1e-05
5e-06
0
1
10
radius (nm)
Range 1.06461e-09 to 2.75379e-08, num-weighted
totalValue: 1.000e+00 ± 5.946e-16
mean: 2.485e-09 ± 6.939e-11
variance: 6.437e-19 ± 6.056e-20
skew: 1.690e-01 ± 4.335e-02
kurtosis: 2.822e+00 ± 3.016e-01
Sphere radius
Minimum visibility limit
MC size histogram
[Rel.] Number Fraction
0.14
0.12
0.1
0.08
0.06
0.04
0.02
0
1
10
radius (nm)
