# Supplementary material for: Nanoparticle size distribution quantification: results of a small-angle X-ray scattering inter-laboratory comparison
Source: J Appl Crystallogr. 2017 Aug 18;50(Pt 5):1280–8. doi: 10.1107/S160057671701010X (PMC5627679; doi:10.1107/S160057671701010X)
Supplement: Supplementary file 3 [file j-50-01280-sup2.zip › RRAnonData/csv/S34_2016-12-02_22-36-27/S34_2016-12-02_22-36-27.pdf]

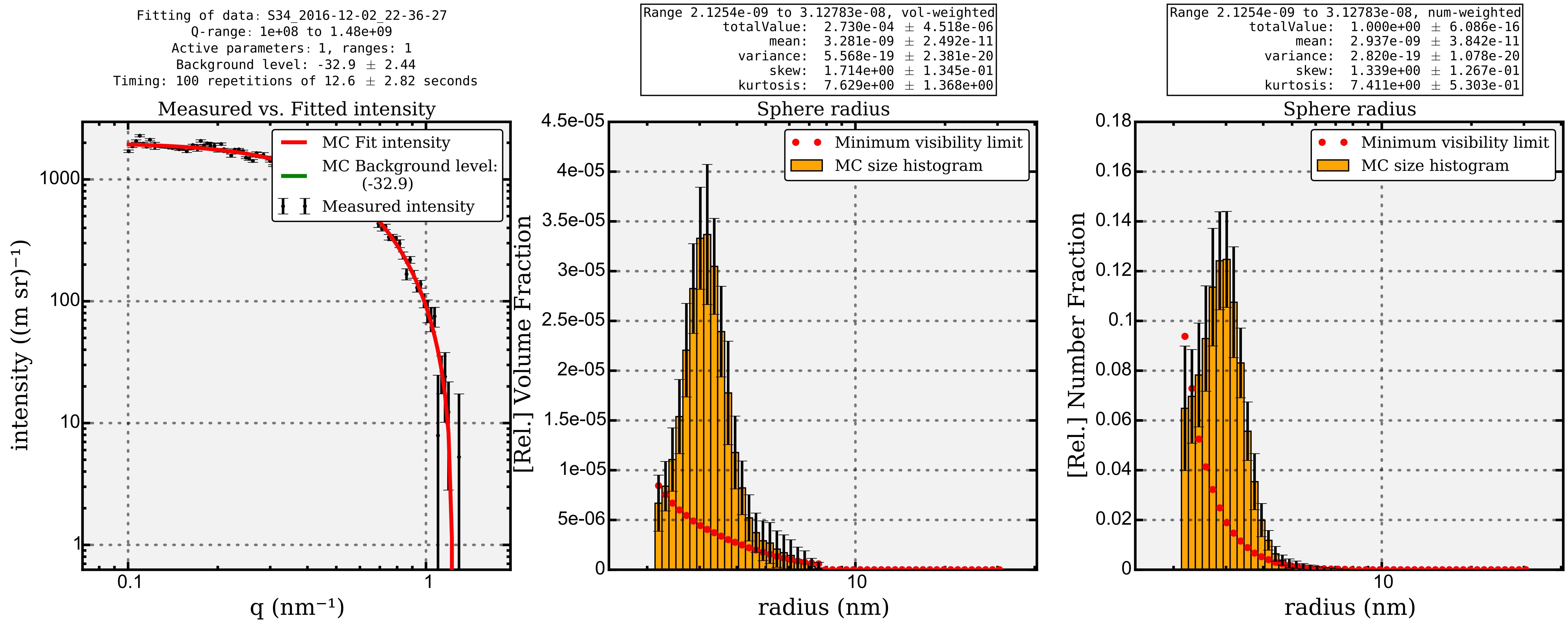

Fitting of data: S34_2016-12-02_22-36-27
Q-range: 1e+08 to 1.48e+09
Active parameters: 1, ranges: 1
Background level: -32.9 ± 2.44
Timing: 100 repetitions of 12.6 ± 2.82 seconds
Measured vs. Fitted intensity
MC Fit intensity
MC Background level: (-32.9)
Measured intensity
intensity ((m sr)⁻¹)
q (nm⁻¹)
Range 2.1254e-09 to 3.12783e-08, vol-weighted
totalValue: 2.730e-04 ± 4.518e-06
mean: 3.281e-09 ± 2.492e-11
variance: 5.568e-19 ± 2.381e-20
skew: 1.714e+00 ± 1.345e-01
kurtosis: 7.629e+00 ± 1.368e+00
Sphere radius
Minimum visibility limit
MC size histogram
[Rel.] Volume Fraction
radius (nm)
Range 2.1254e-09 to 3.12783e-08, num-weighted
totalValue: 1.000e+00 ± 6.086e-16
mean: 2.937e-09 ± 3.842e-11
variance: 2.820e-19 ± 1.078e-20
skew: 1.339e+00 ± 1.267e-01
kurtosis: 7.411e+00 ± 5.303e-01
Sphere radius
Minimum visibility limit
MC size histogram
[Rel.] Number Fraction
radius (nm)
