# Supplementary material for: Nanoparticle size distribution quantification: results of a small-angle X-ray scattering inter-laboratory comparison
Source: J Appl Crystallogr. 2017 Aug 18;50(Pt 5):1280–8. doi: 10.1107/S160057671701010X (PMC5627679; doi:10.1107/S160057671701010X)
Supplement: Supplementary file 3 [file j-50-01280-sup2.zip › RRAnonData/csv/S35_2016-12-02_22-57-35/S35_2016-12-02_22-57-35.pdf]

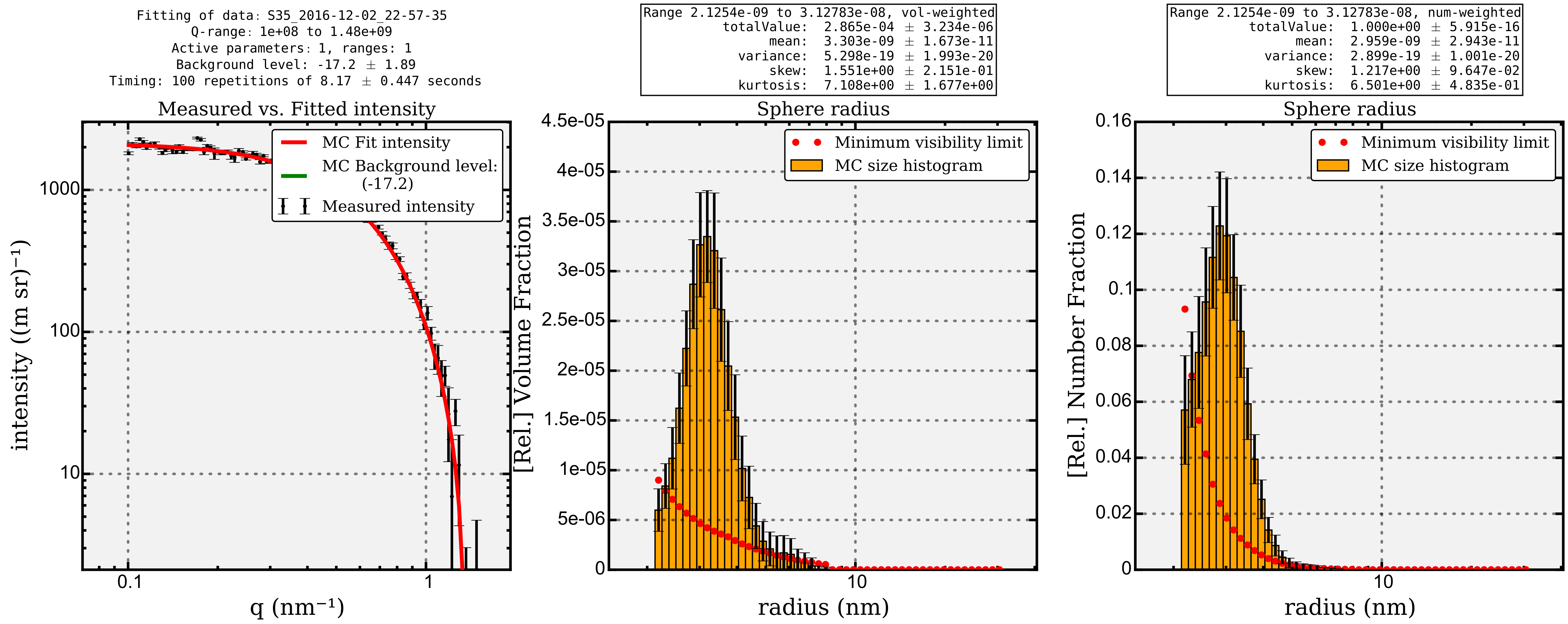
Fitting of data: S35_2016-12-02_22-57-35
Q-range: 1e+08 to 1.48e+09
Active parameters: 1, ranges: 1
Background level: -17.2 ± 1.89
Timing: 100 repetitions of 8.17 ± 0.447 seconds
Measured vs. Fitted intensity
MC Fit intensity
MC Background level: (-17.2)
Measured intensity
intensity ((m sr)$^{-1}$)
q (nm^{-1})
1000
100
10
0.1
1
Range 2.1254e-09 to 3.12783e-08, vol-weighted
totalValue: 2.865e-04 ± 3.234e-06
mean: 3.303e-09 ± 1.673e-11
variance: 5.298e-19 ± 1.993e-20
skew: 1.551e+00 ± 2.151e-01
kurtosis: 7.108e+00 ± 1.677e+00
Sphere radius
Minimum visibility limit
MC size histogram
[Rel.] Volume Fraction
radius (nm)
4.5e-05
4e-05
3.5e-05
3e-05
2.5e-05
2e-05
1.5e-05
1e-05
5e-06
0
10
Range 2.1254e-09 to 3.12783e-08, num-weighted
totalValue: 1.000e+00 ± 5.915e-16
mean: 2.959e-09 ± 2.943e-11
variance: 2.899e-19 ± 1.001e-20
skew: 1.217e+00 ± 9.647e-02
kurtosis: 6.501e+00 ± 4.835e-01
Sphere radius
Minimum visibility limit
MC size histogram
[Rel.] Number Fraction
radius (nm)
0.16
0.14
0.12
0.1
0.08
0.06
0.04
0.02
0
10
