# Supplementary material for: Nanoparticle size distribution quantification: results of a small-angle X-ray scattering inter-laboratory comparison
Source: J Appl Crystallogr. 2017 Aug 18;50(Pt 5):1280–8. doi: 10.1107/S160057671701010X (PMC5627679; doi:10.1107/S160057671701010X)
Supplement: Supplementary file 3 [file j-50-01280-sup2.zip › RRAnonData/csv/S36_2016-12-03_08-27-40/S36_2016-12-03_08-27-40.pdf]

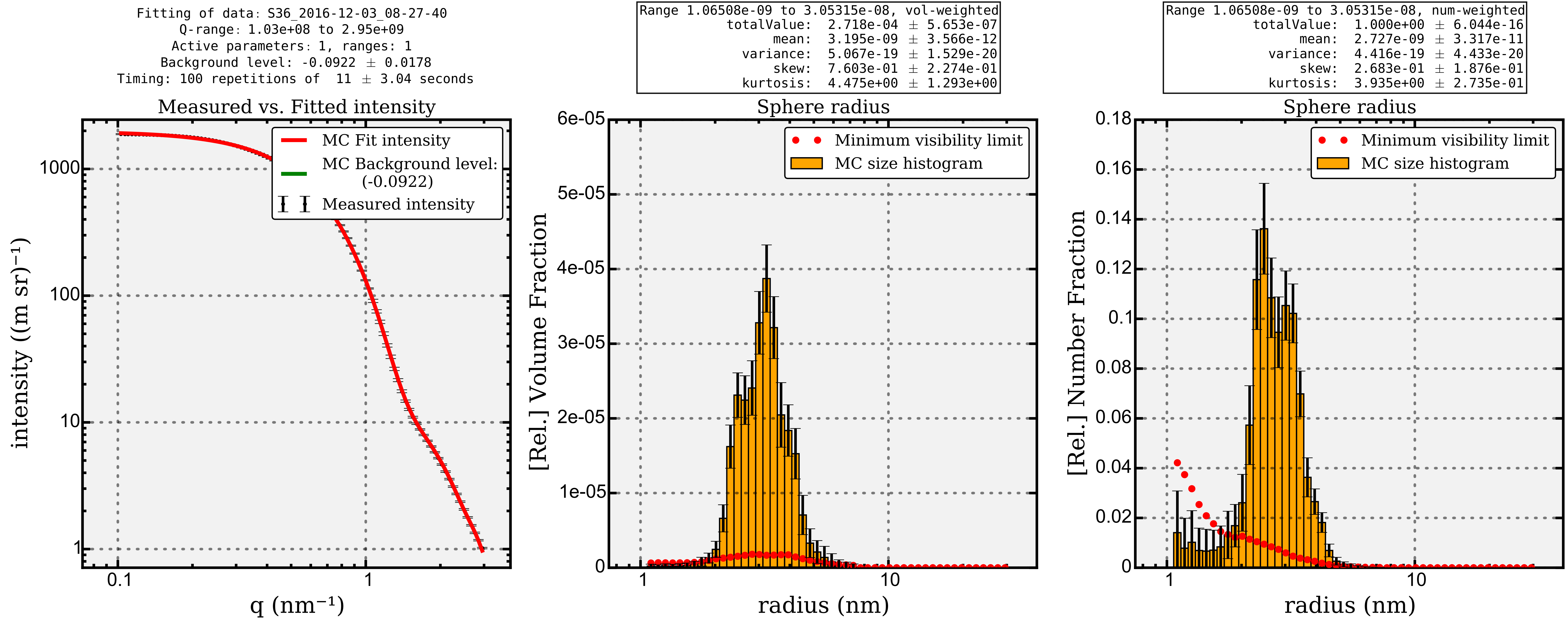
Fitting of data: S36_2016-12-03_08-27-40
Q-range: 1.03e+08 to 2.95e+09
Active parameters: 1, ranges: 1
Background level: -0.0922 ± 0.0178
Timing: 100 repetitions of 11 ± 3.04 seconds
Measured vs. Fitted intensity
MC Fit intensity
MC Background level: (-0.0922)
Measured intensity
intensity ((m sr)⁻¹)
q (nm⁻¹)
Range 1.06508e-09 to 3.05315e-08, vol-weighted
totalValue: 2.718e-04 ± 5.653e-07
mean: 3.195e-09 ± 3.566e-12
variance: 5.067e-19 ± 1.529e-20
skew: 7.603e-01 ± 2.274e-01
kurtosis: 4.475e+00 ± 1.293e+00
Sphere radius
Minimum visibility limit
MC size histogram
[Rel.] Volume Fraction
radius (nm)
Range 1.06508e-09 to 3.05315e-08, num-weighted
totalValue: 1.000e+00 ± 6.044e-16
mean: 2.727e-09 ± 3.317e-11
variance: 4.416e-19 ± 4.433e-20
skew: 2.683e-01 ± 1.876e-01
kurtosis: 3.935e+00 ± 2.735e-01
Sphere radius
Minimum visibility limit
MC size histogram
[Rel.] Number Fraction
radius (nm)
