# Supplementary material for: Nanoparticle size distribution quantification: results of a small-angle X-ray scattering inter-laboratory comparison
Source: J Appl Crystallogr. 2017 Aug 18;50(Pt 5):1280–8. doi: 10.1107/S160057671701010X (PMC5627679; doi:10.1107/S160057671701010X)
Supplement: Supplementary file 3 [file j-50-01280-sup2.zip › RRAnonData/csv/S37_2016-12-03_08-28-05/S37_2016-12-03_08-28-05.pdf]

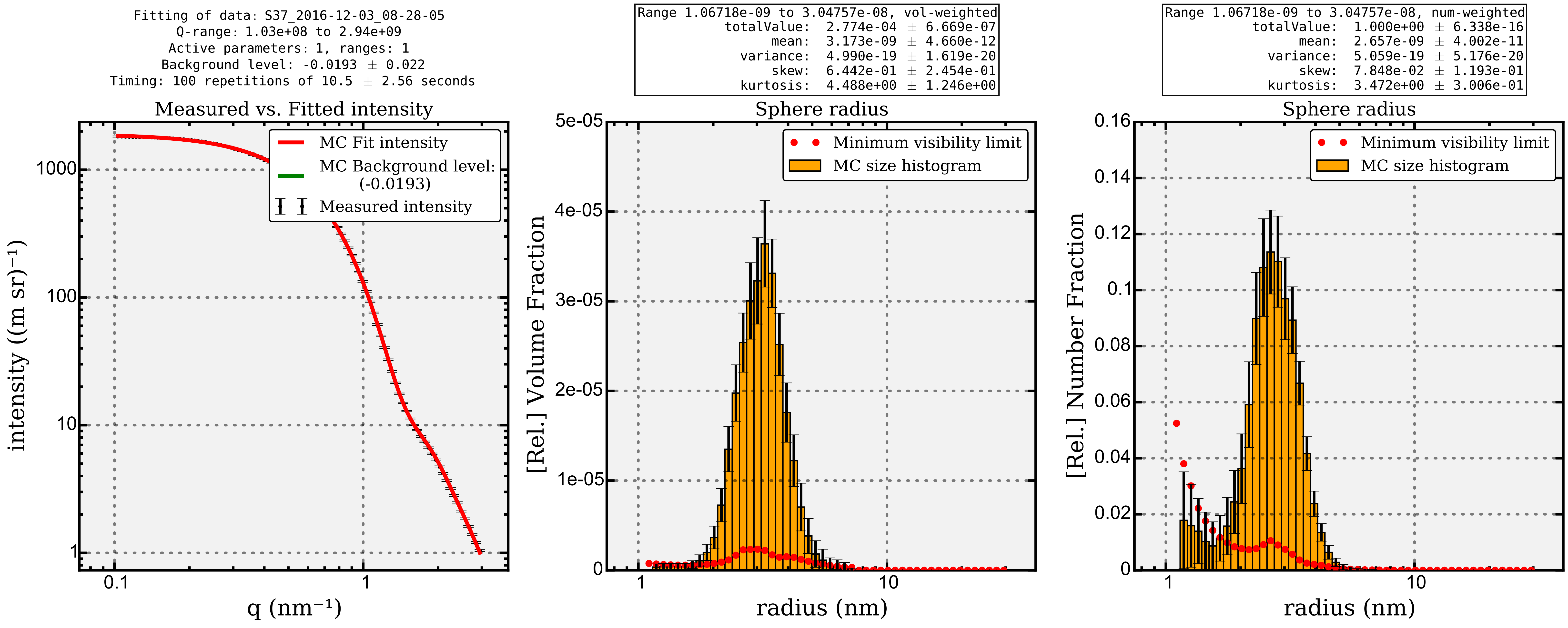

Fitting of data: S37_2016-12-03_08-28-05
Q-range: 1.03e+08 to 2.94e+09
Active parameters: 1, ranges: 1
Background level: -0.0193 ± 0.022
Timing: 100 repetitions of 10.5 ± 2.56 seconds
Measured vs. Fitted intensity
MC Fit intensity
MC Background level: (-0.0193)
Measured intensity
intensity ((m sr)$^{-1}$)
q (nm^{-1})
1000
100
10
1
0.1
1
Range 1.06718e-09 to 3.04757e-08, vol-weighted
totalValue: 2.774e-04 ± 6.669e-07
mean: 3.173e-09 ± 4.660e-12
variance: 4.990e-19 ± 1.619e-20
skew: 6.442e-01 ± 2.454e-01
kurtosis: 4.488e+00 ± 1.246e+00
Sphere radius
Minimum visibility limit
MC size histogram
[Rel.] Volume Fraction
5e-05
4e-05
3e-05
2e-05
1e-05
0
1
10
radius (nm)
Range 1.06718e-09 to 3.04757e-08, num-weighted
totalValue: 1.000e+00 ± 6.338e-16
mean: 2.657e-09 ± 4.002e-11
variance: 5.059e-19 ± 5.176e-20
skew: 7.848e-02 ± 1.193e-01
kurtosis: 3.472e+00 ± 3.006e-01
Sphere radius
Minimum visibility limit
MC size histogram
[Rel.] Number Fraction
0.16
0.14
0.12
0.1
0.08
0.06
0.04
0.02
0
1
10
radius (nm)
