# Supplementary material for: Nanoparticle size distribution quantification: results of a small-angle X-ray scattering inter-laboratory comparison
Source: J Appl Crystallogr. 2017 Aug 18;50(Pt 5):1280–8. doi: 10.1107/S160057671701010X (PMC5627679; doi:10.1107/S160057671701010X)
Supplement: Supplementary file 3 [file j-50-01280-sup2.zip › RRAnonData/csv/S38_2016-12-03_08-28-45/S38_2016-12-03_08-28-45.pdf]

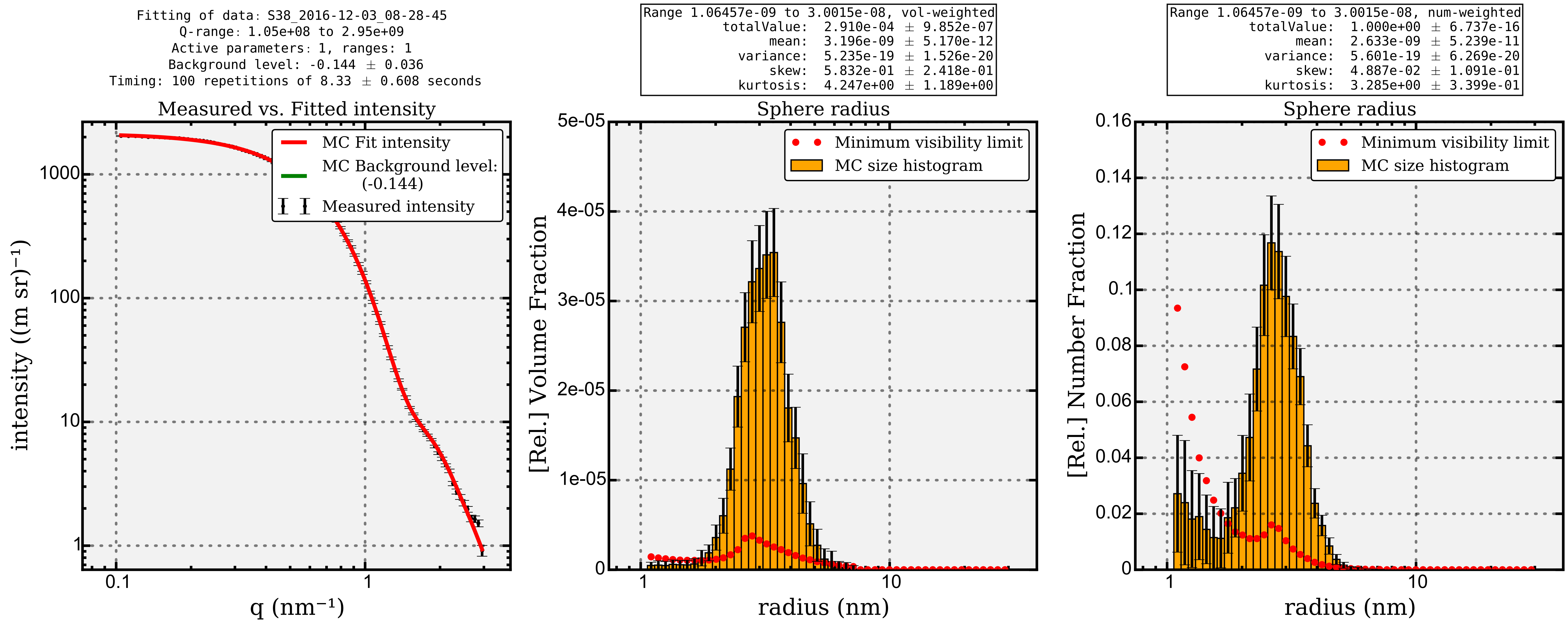
Fitting of data: S38_2016-12-03_08-28-45
Q-range: 1.05e+08 to 2.95e+09
Active parameters: 1, ranges: 1
Background level: -0.144 ± 0.036
Timing: 100 repetitions of 8.33 ± 0.608 seconds
Measured vs. Fitted intensity
MC Fit intensity
MC Background level: (-0.144)
Measured intensity
intensity ((m sr)$^{-1}$)
q (nm^{-1})
1000
100
10
1
0.1
1
Range 1.06457e-09 to 3.0015e-08, vol-weighted
totalValue: 2.910e-04 ± 9.852e-07
mean: 3.196e-09 ± 5.170e-12
variance: 5.235e-19 ± 1.526e-20
skew: 5.832e-01 ± 2.418e-01
kurtosis: 4.247e+00 ± 1.189e+00
Sphere radius
Minimum visibility limit
MC size histogram
[Rel.] Volume Fraction
radius (nm)
5e-05
4e-05
3e-05
2e-05
1e-05
0
1
10
Range 1.06457e-09 to 3.0015e-08, num-weighted
totalValue: 1.000e+00 ± 6.737e-16
mean: 2.633e-09 ± 5.239e-11
variance: 5.601e-19 ± 6.269e-20
skew: 4.887e-02 ± 1.091e-01
kurtosis: 3.285e+00 ± 3.399e-01
Sphere radius
Minimum visibility limit
MC size histogram
[Rel.] Number Fraction
radius (nm)
0.16
0.14
0.12
0.1
0.08
0.06
0.04
0.02
0
1
10
