# Supplementary material for: Nanoparticle size distribution quantification: results of a small-angle X-ray scattering inter-laboratory comparison
Source: J Appl Crystallogr. 2017 Aug 18;50(Pt 5):1280–8. doi: 10.1107/S160057671701010X (PMC5627679; doi:10.1107/S160057671701010X)
Supplement: Supplementary file 3 [file j-50-01280-sup2.zip › RRAnonData/csv/S41_2016-12-03_08-47-06/S41_2016-12-03_08-47-06.pdf]

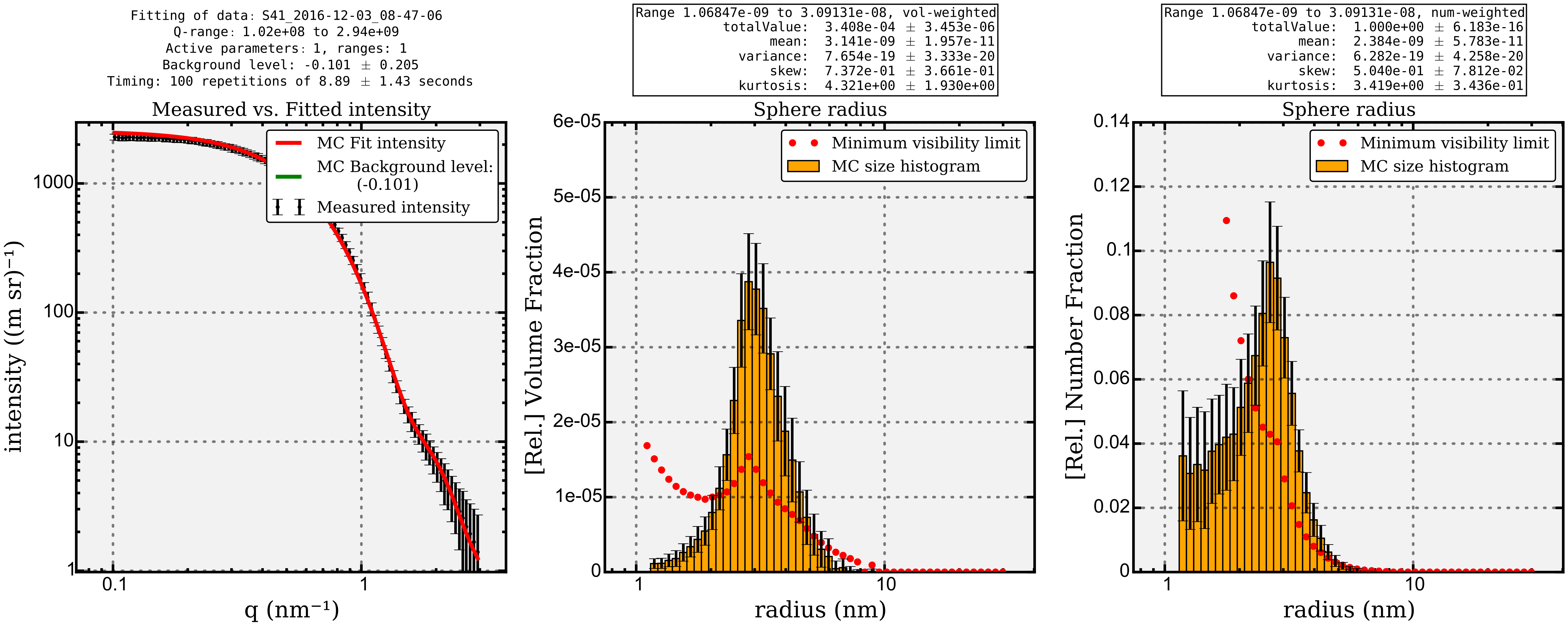

Fitting of data: S41_2016-12-03_08-47-06
Q-range: 1.02e+08 to 2.94e+09
Active parameters: 1, ranges: 1
Background level: -0.101 ± 0.205
Timing: 100 repetitions of 8.89 ± 1.43 seconds
Measured vs. Fitted intensity
MC Fit intensity
MC Background level: (-0.101)
Measured intensity
intensity ((m sr)⁻¹)
q (nm⁻¹)
Range 1.06847e-09 to 3.09131e-08, vol-weighted
totalValue: 3.408e-04 ± 3.453e-06
mean: 3.141e-09 ± 1.957e-11
variance: 7.654e-19 ± 3.333e-20
skew: 7.372e-01 ± 3.661e-01
kurtosis: 4.321e+00 ± 1.930e+00
Sphere radius
Minimum visibility limit
MC size histogram
[Rel.] Volume Fraction
radius (nm)
Range 1.06847e-09 to 3.09131e-08, num-weighted
totalValue: 1.000e+00 ± 6.183e-16
mean: 2.384e-09 ± 5.783e-11
variance: 6.282e-19 ± 4.258e-20
skew: 5.040e-01 ± 7.812e-02
kurtosis: 3.419e+00 ± 3.436e-01
Sphere radius
Minimum visibility limit
MC size histogram
[Rel.] Number Fraction
radius (nm)
