# Supplementary material for: Nanoparticle size distribution quantification: results of a small-angle X-ray scattering inter-laboratory comparison
Source: J Appl Crystallogr. 2017 Aug 18;50(Pt 5):1280–8. doi: 10.1107/S160057671701010X (PMC5627679; doi:10.1107/S160057671701010X)
Supplement: Supplementary file 3 [file j-50-01280-sup2.zip › RRAnonData/csv/S43_2016-12-03_08-47-40/S43_2016-12-03_08-47-40.pdf]

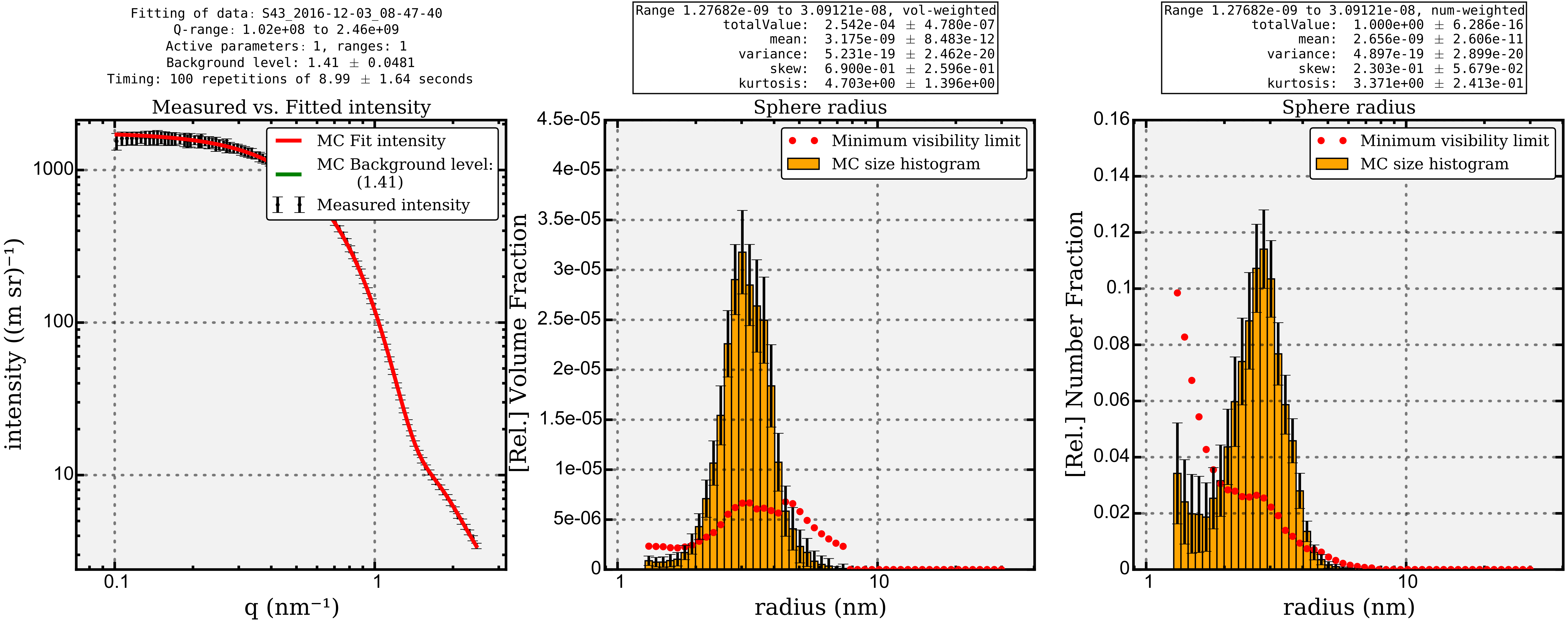

Fitting of data: S43_2016-12-03_08-47-40
Q-range: 1.02e+08 to 2.46e+09
Active parameters: 1, ranges: 1
Background level: 1.41 ± 0.0481
Timing: 100 repetitions of 8.99 ± 1.64 seconds
Measured vs. Fitted intensity
MC Fit intensity
MC Background level: (1.41)
Measured intensity
intensity ((m sr)$^{-1}$)
q (nm^{-1})
1000
100
10
0.1
1
Range 1.27682e-09 to 3.09121e-08, vol-weighted
totalValue: 2.542e-04 ± 4.780e-07
mean: 3.175e-09 ± 8.483e-12
variance: 5.231e-19 ± 2.462e-20
skew: 6.900e-01 ± 2.596e-01
kurtosis: 4.703e+00 ± 1.396e+00
Sphere radius
Minimum visibility limit
MC size histogram
[Rel.] Volume Fraction
radius (nm)
4.5e-05
4e-05
3.5e-05
3e-05
2.5e-05
2e-05
1.5e-05
1e-05
5e-06
0
1
10
Range 1.27682e-09 to 3.09121e-08, num-weighted
totalValue: 1.000e+00 ± 6.286e-16
mean: 2.656e-09 ± 2.606e-11
variance: 4.897e-19 ± 2.899e-20
skew: 2.303e-01 ± 5.679e-02
kurtosis: 3.371e+00 ± 2.413e-01
Sphere radius
Minimum visibility limit
MC size histogram
[Rel.] Number Fraction
radius (nm)
0.16
0.14
0.12
0.1
0.08
0.06
0.04
0.02
0
1
10
