# Supplementary material for: Nanoparticle size distribution quantification: results of a small-angle X-ray scattering inter-laboratory comparison
Source: J Appl Crystallogr. 2017 Aug 18;50(Pt 5):1280–8. doi: 10.1107/S160057671701010X (PMC5627679; doi:10.1107/S160057671701010X)
Supplement: Supplementary file 3 [file j-50-01280-sup2.zip › RRAnonData/csv/S45_2016-12-03_09-02-50/S45_2016-12-03_09-02-50.pdf]

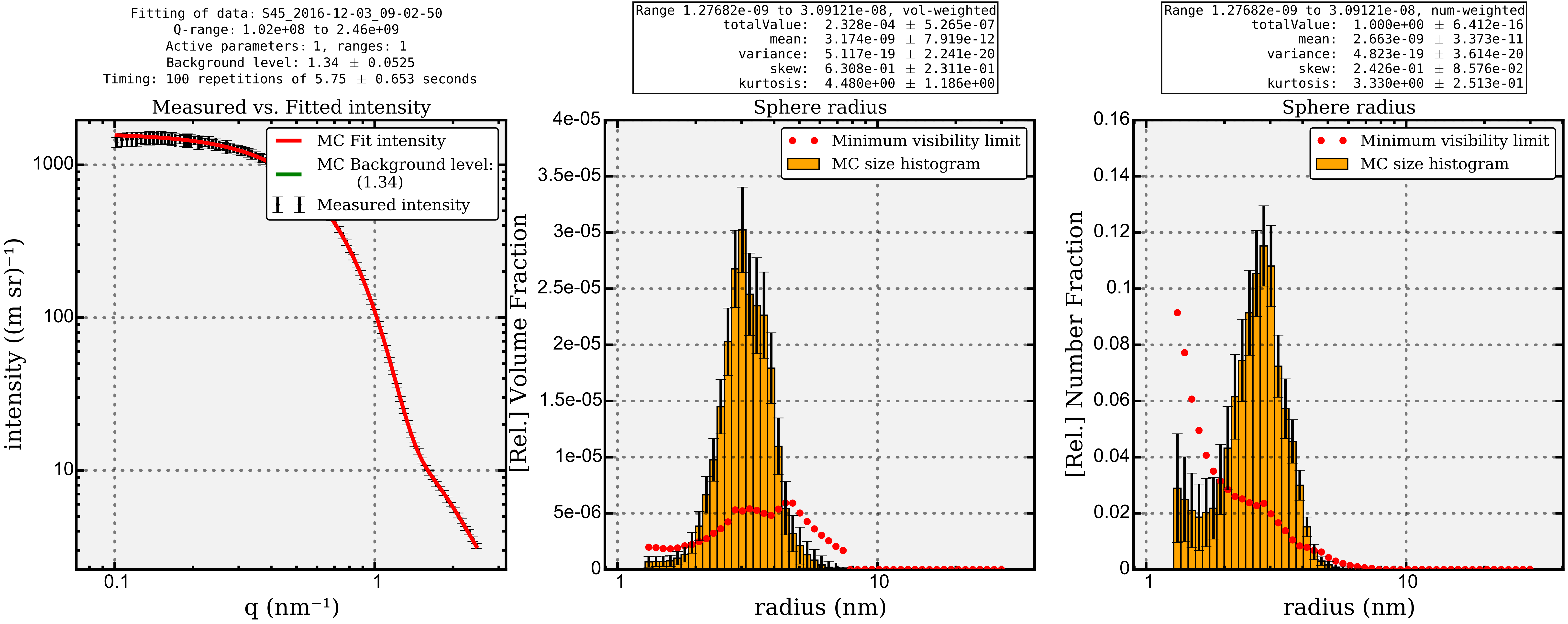
Fitting of data: S45_2016-12-03_09-02-50
Q-range: 1.02e+08 to 2.46e+09
Active parameters: 1, ranges: 1
Background level: 1.34 ± 0.0525
Timing: 100 repetitions of 5.75 ± 0.653 seconds
Measured vs. Fitted intensity
MC Fit intensity
MC Background level: (1.34)
Measured intensity
intensity ((m sr)$^{-1}$)
q (nm^{-1})
Range 1.27682e-09 to 3.09121e-08, vol-weighted
totalValue: 2.328e-04 ± 5.265e-07
mean: 3.174e-09 ± 7.919e-12
variance: 5.117e-19 ± 2.241e-20
skew: 6.308e-01 ± 2.311e-01
kurtosis: 4.480e+00 ± 1.186e+00
Sphere radius
Minimum visibility limit
MC size histogram
[Rel.] Volume Fraction
radius (nm)
Range 1.27682e-09 to 3.09121e-08, num-weighted
totalValue: 1.000e+00 ± 6.412e-16
mean: 2.663e-09 ± 3.373e-11
variance: 4.823e-19 ± 3.614e-20
skew: 2.426e-01 ± 8.576e-02
kurtosis: 3.330e+00 ± 2.513e-01
Sphere radius
Minimum visibility limit
MC size histogram
[Rel.] Number Fraction
radius (nm)
