# Supplementary material for: Nanoparticle size distribution quantification: results of a small-angle X-ray scattering inter-laboratory comparison
Source: J Appl Crystallogr. 2017 Aug 18;50(Pt 5):1280–8. doi: 10.1107/S160057671701010X (PMC5627679; doi:10.1107/S160057671701010X)
Supplement: Supplementary file 3 [file j-50-01280-sup2.zip › RRAnonData/csv/S46_2016-12-03_08-48-49/S46_2016-12-03_08-48-49.pdf]

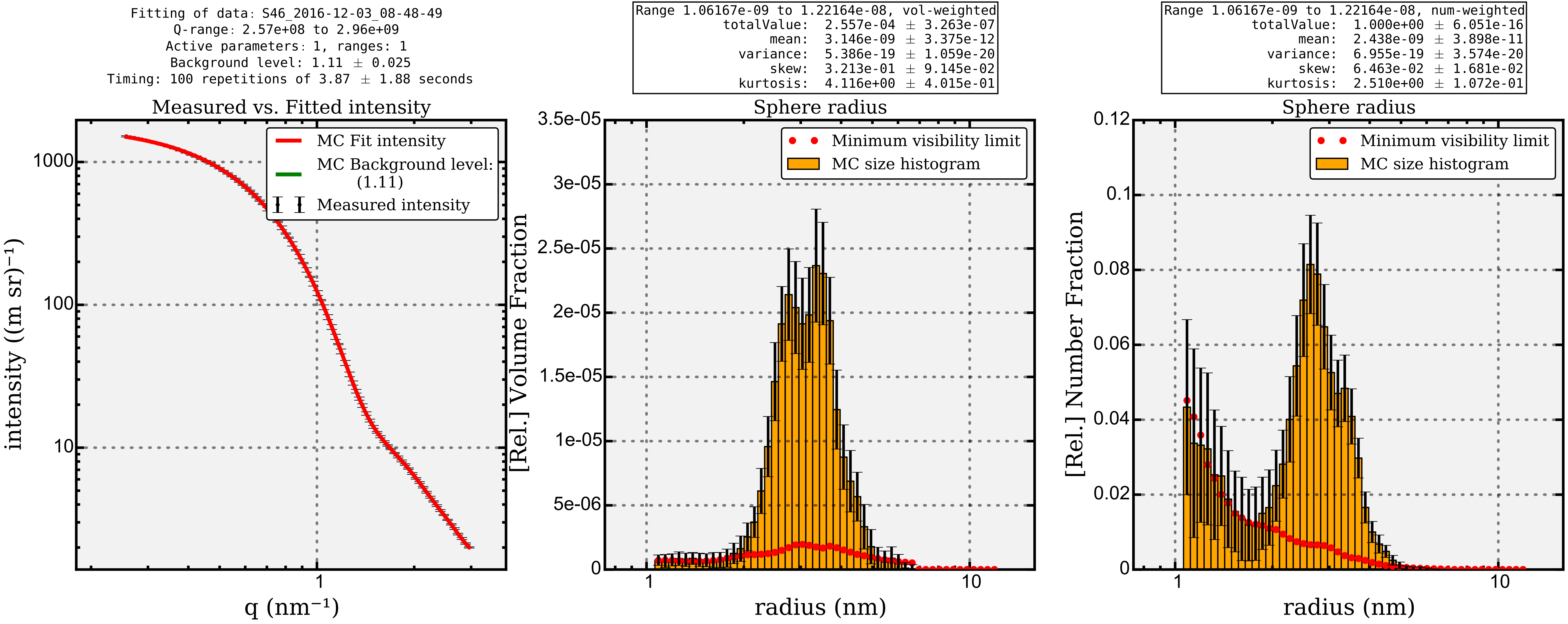
Fitting of data: S46_2016-12-03_08-48-49
Q-range: 2.57e+08 to 2.96e+09
Active parameters: 1, ranges: 1
Background level: 1.11 ± 0.025
Timing: 100 repetitions of 3.87 ± 1.88 seconds
Measured vs. Fitted intensity
MC Fit intensity
MC Background level: (1.11)
Measured intensity
intensity ((m sr)⁻¹)
q (nm⁻¹)
1000
100
10
1
Range 1.06167e-09 to 1.22164e-08, vol-weighted
totalValue: 2.557e-04 ± 3.263e-07
mean: 3.146e-09 ± 3.375e-12
variance: 5.386e-19 ± 1.059e-20
skew: 3.213e-01 ± 9.145e-02
kurtosis: 4.116e+00 ± 4.015e-01
Sphere radius
Minimum visibility limit
MC size histogram
[Rel.] Volume Fraction
radius (nm)
3.5e-05
3e-05
2.5e-05
2e-05
1.5e-05
1e-05
5e-06
0
1
10
Range 1.06167e-09 to 1.22164e-08, num-weighted
totalValue: 1.000e+00 ± 6.051e-16
mean: 2.438e-09 ± 3.898e-11
variance: 6.955e-19 ± 3.574e-20
skew: 6.463e-02 ± 1.681e-02
kurtosis: 2.510e+00 ± 1.072e-01
Sphere radius
Minimum visibility limit
MC size histogram
[Rel.] Number Fraction
radius (nm)
0.12
0.1
0.08
0.06
0.04
0.02
0
1
10
