# Supplementary material for: Nanoparticle size distribution quantification: results of a small-angle X-ray scattering inter-laboratory comparison
Source: J Appl Crystallogr. 2017 Aug 18;50(Pt 5):1280–8. doi: 10.1107/S160057671701010X (PMC5627679; doi:10.1107/S160057671701010X)
Supplement: Supplementary file 3 [file j-50-01280-sup2.zip › RRAnonData/csv/S47_2016-12-03_08-55-30/S47_2016-12-03_08-55-30.pdf]

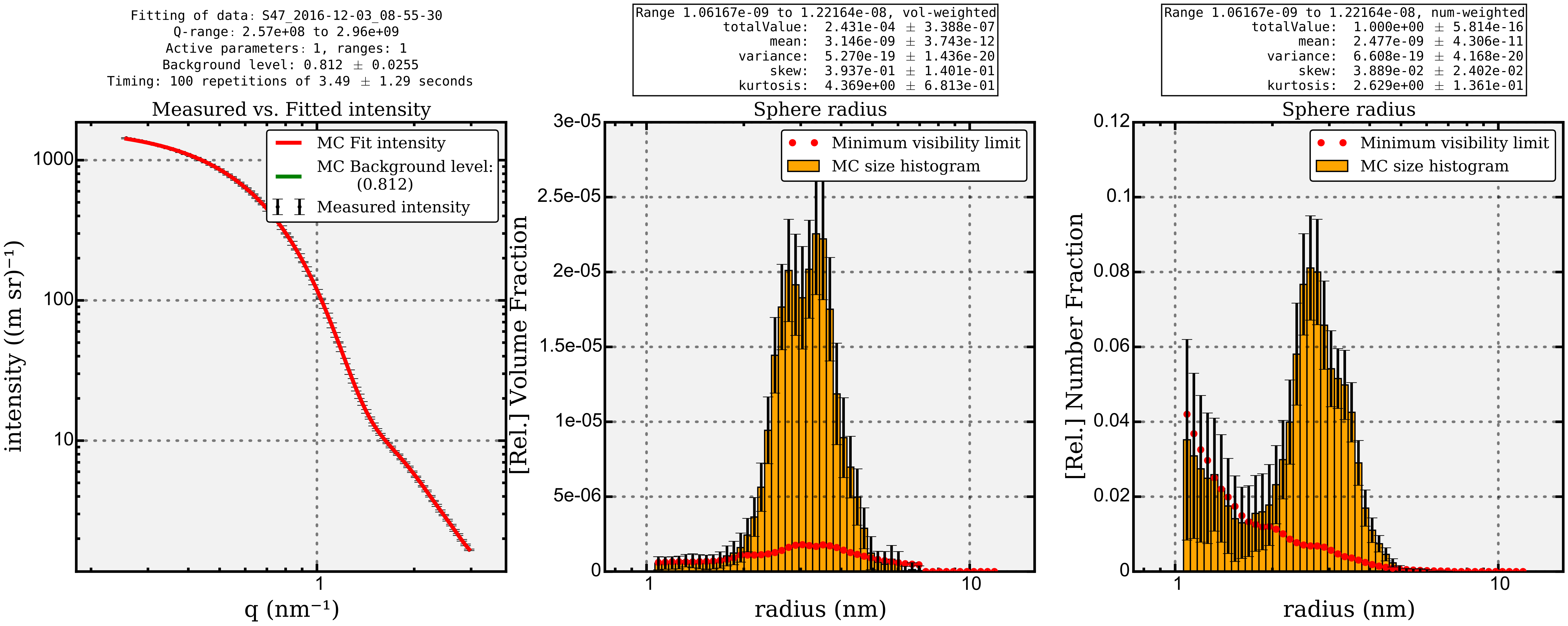
Fitting of data: S47_2016-12-03_08-55-30
Q-range: 2.57e+08 to 2.96e+09
Active parameters: 1, ranges: 1
Background level: 0.812 ± 0.0255
Timing: 100 repetitions of 3.49 ± 1.29 seconds
Measured vs. Fitted intensity
MC Fit intensity
MC Background level: (0.812)
Measured intensity
intensity ((m sr)$^{-1}$)
1000
100
10
1
q (nm^{-1})
Range 1.06167e-09 to 1.22164e-08, vol-weighted
totalValue: 2.431e-04 ± 3.388e-07
mean: 3.146e-09 ± 3.743e-12
variance: 5.270e-19 ± 1.436e-20
skew: 3.937e-01 ± 1.401e-01
kurtosis: 4.369e+00 ± 6.813e-01
Sphere radius
Minimum visibility limit
MC size histogram
[Rel.] Volume Fraction
3e-05
2.5e-05
2e-05
1.5e-05
1e-05
5e-06
0
1
10
radius (nm)
Range 1.06167e-09 to 1.22164e-08, num-weighted
totalValue: 1.000e+00 ± 5.814e-16
mean: 2.477e-09 ± 4.306e-11
variance: 6.608e-19 ± 4.168e-20
skew: 3.889e-02 ± 2.402e-02
kurtosis: 2.629e+00 ± 1.361e-01
Sphere radius
Minimum visibility limit
MC size histogram
[Rel.] Number Fraction
0.12
0.1
0.08
0.06
0.04
0.02
0
1
10
radius (nm)
