# Supplementary material for: Nanoparticle size distribution quantification: results of a small-angle X-ray scattering inter-laboratory comparison
Source: J Appl Crystallogr. 2017 Aug 18;50(Pt 5):1280–8. doi: 10.1107/S160057671701010X (PMC5627679; doi:10.1107/S160057671701010X)
Supplement: Supplementary file 3 [file j-50-01280-sup2.zip › RRAnonData/csv/S49_2016-12-03_09-22-31/S49_2016-12-03_09-22-31.pdf]

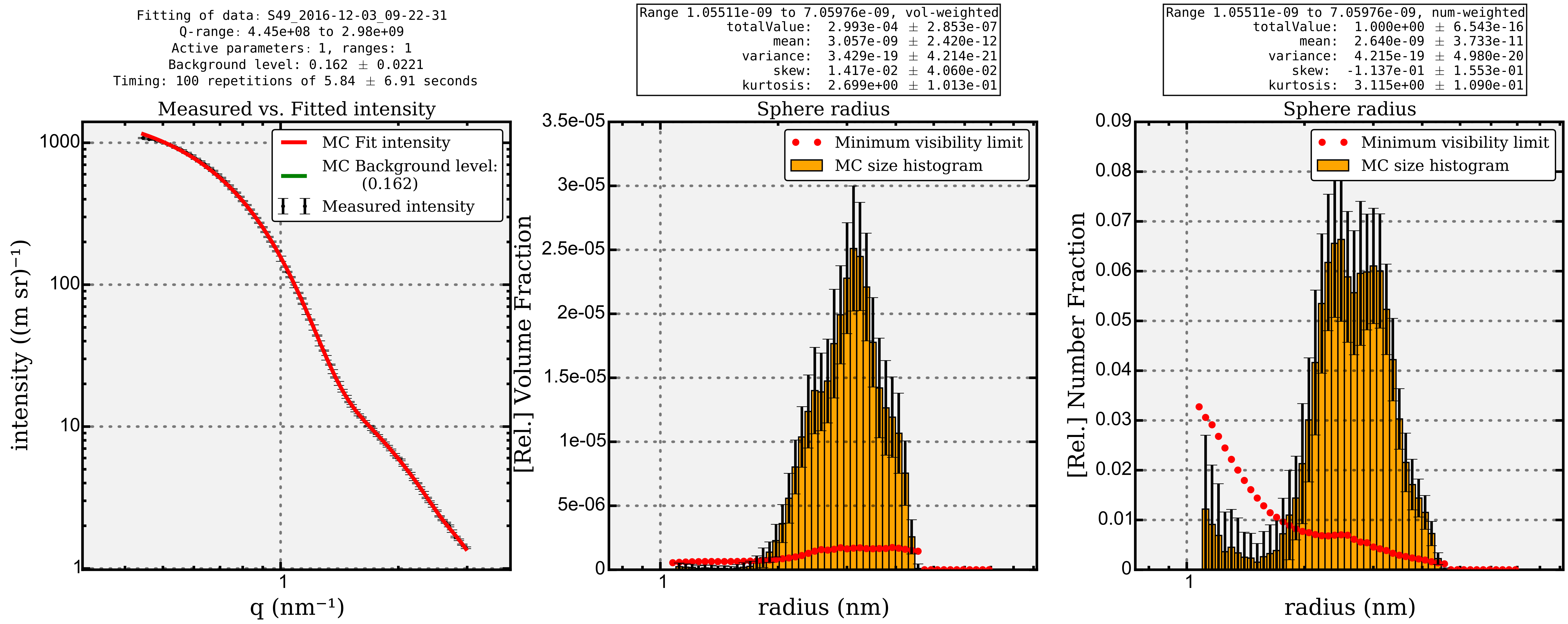
Fitting of data: S49_2016-12-03_09-22-31
Q-range: 4.45e+08 to 2.98e+09
Active parameters: 1, ranges: 1
Background level: 0.162 ± 0.0221
Timing: 100 repetitions of 5.84 ± 6.91 seconds
Measured vs. Fitted intensity
MC Fit intensity
MC Background level: (0.162)
Measured intensity
intensity ((m sr)⁻¹)
q (nm⁻¹)
Range 1.05511e-09 to 7.05976e-09, vol-weighted
totalValue: 2.993e-04 ± 2.853e-07
mean: 3.057e-09 ± 2.420e-12
variance: 3.429e-19 ± 4.214e-21
skew: 1.417e-02 ± 4.060e-02
kurtosis: 2.699e+00 ± 1.013e-01
Sphere radius
Minimum visibility limit
MC size histogram
[Rel.] Volume Fraction
radius (nm)
Range 1.05511e-09 to 7.05976e-09, num-weighted
totalValue: 1.000e+00 ± 6.543e-16
mean: 2.640e-09 ± 3.733e-11
variance: 4.215e-19 ± 4.980e-20
skew: -1.137e-01 ± 1.553e-01
kurtosis: 3.115e+00 ± 1.090e-01
Sphere radius
Minimum visibility limit
MC size histogram
[Rel.] Number Fraction
radius (nm)
