# Supplementary material for: Nanoparticle size distribution quantification: results of a small-angle X-ray scattering inter-laboratory comparison
Source: J Appl Crystallogr. 2017 Aug 18;50(Pt 5):1280–8. doi: 10.1107/S160057671701010X (PMC5627679; doi:10.1107/S160057671701010X)
Supplement: Supplementary file 3 [file j-50-01280-sup2.zip › RRAnonData/csv/S51_2016-12-03_09-32-34/S51_2016-12-03_09-32-34.pdf]

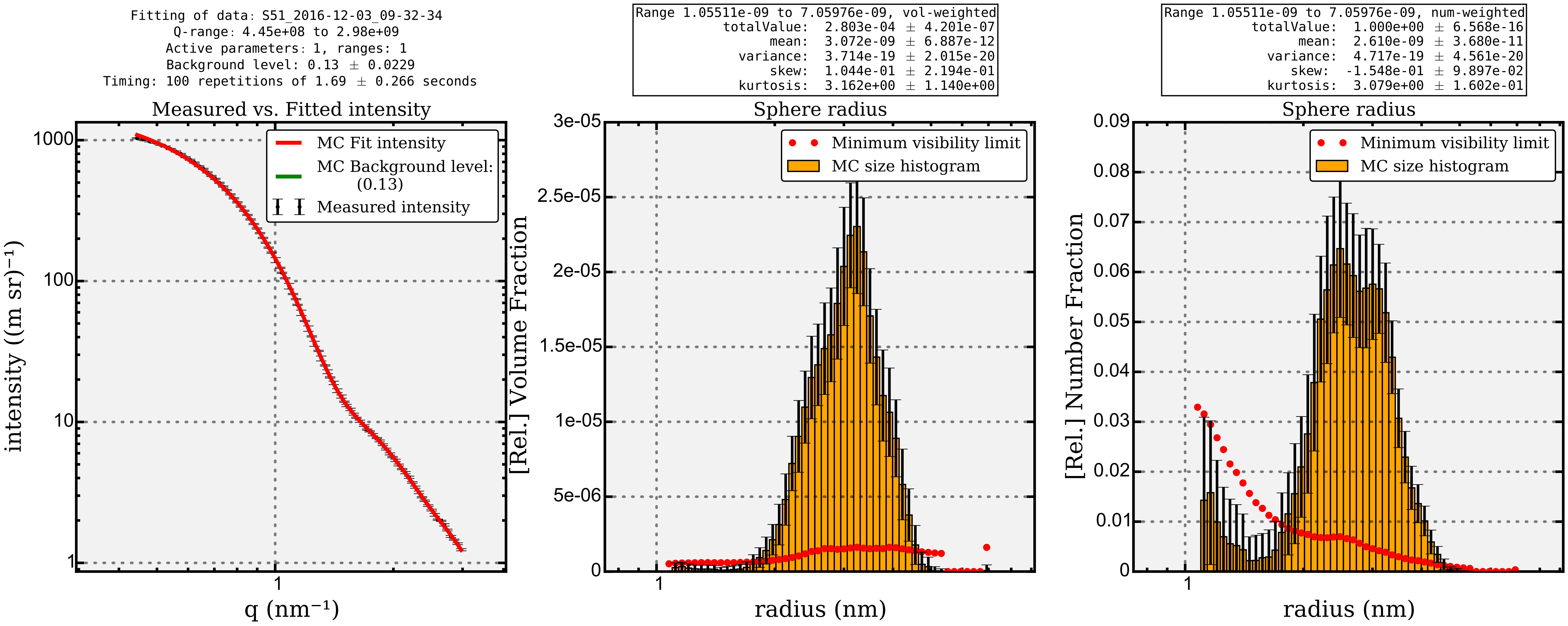
Fitting of data: S51_2016-12-03_09-32-34
Q-range: 4.45e+08 to 2.98e+09
Active parameters: 1, ranges: 1
Background level: 0.13 ± 0.0229
Timing: 100 repetitions of 1.69 ± 0.266 seconds
Measured vs. Fitted intensity
MC Fit intensity
MC Background level: (0.13)
Measured intensity
intensity ((m sr)⁻¹)
q (nm⁻¹)
1000
100
10
1
Range 1.05511e-09 to 7.05976e-09, vol-weighted
totalValue: 2.803e-04 ± 4.201e-07
mean: 3.072e-09 ± 6.887e-12
variance: 3.714e-19 ± 2.015e-20
skew: 1.044e-01 ± 2.194e-01
kurtosis: 3.162e+00 ± 1.140e+00
Sphere radius
Minimum visibility limit
MC size histogram
[Rel.] Volume Fraction
radius (nm)
3e-05
2.5e-05
2e-05
1.5e-05
1e-05
5e-06
0
Range 1.05511e-09 to 7.05976e-09, num-weighted
totalValue: 1.000e+00 ± 6.568e-16
mean: 2.610e-09 ± 3.680e-11
variance: 4.717e-19 ± 4.561e-20
skew: -1.548e-01 ± 9.897e-02
kurtosis: 3.079e+00 ± 1.602e-01
Sphere radius
Minimum visibility limit
MC size histogram
[Rel.] Number Fraction
radius (nm)
0.09
0.08
0.07
0.06
0.05
0.04
0.03
0.02
0.01
0
