# Supplementary material for: Nanoparticle size distribution quantification: results of a small-angle X-ray scattering inter-laboratory comparison
Source: J Appl Crystallogr. 2017 Aug 18;50(Pt 5):1280–8. doi: 10.1107/S160057671701010X (PMC5627679; doi:10.1107/S160057671701010X)
Supplement: Supplementary file 3 [file j-50-01280-sup2.zip › RRAnonData/csv/S52_2016-12-03_09-23-39/S52_2016-12-03_09-23-39.pdf]

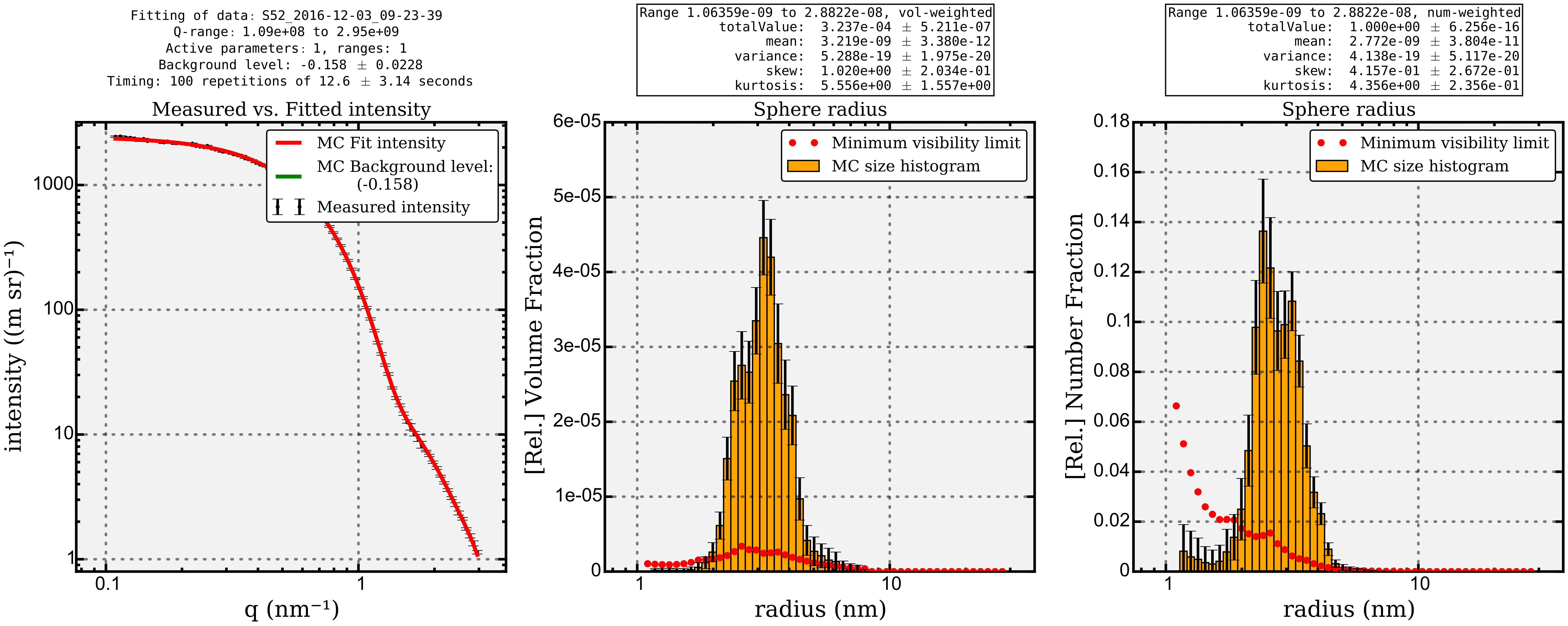
Fitting of data: S52_2016-12-03_09-23-39
Q-range: 1.09e+08 to 2.95e+09
Active parameters: 1, ranges: 1
Background level: -0.158 ± 0.0228
Timing: 100 repetitions of 12.6 ± 3.14 seconds
Measured vs. Fitted intensity
MC Fit intensity
MC Background level: (-0.158)
Measured intensity
intensity ((m sr)⁻¹)
q (nm⁻¹)
1000
100
10
1
0.1
1
Range 1.06359e-09 to 2.8822e-08, vol-weighted
totalValue: 3.237e-04 ± 5.211e-07
mean: 3.219e-09 ± 3.380e-12
variance: 5.288e-19 ± 1.975e-20
skew: 1.020e+00 ± 2.034e-01
kurtosis: 5.556e+00 ± 1.557e+00
Sphere radius
Minimum visibility limit
MC size histogram
[Rel.] Volume Fraction
radius (nm)
6e-05
5e-05
4e-05
3e-05
2e-05
1e-05
0
1
10
Range 1.06359e-09 to 2.8822e-08, num-weighted
totalValue: 1.000e+00 ± 6.256e-16
mean: 2.772e-09 ± 3.804e-11
variance: 4.138e-19 ± 5.117e-20
skew: 4.157e-01 ± 2.672e-01
kurtosis: 4.356e+00 ± 2.356e-01
Sphere radius
Minimum visibility limit
MC size histogram
[Rel.] Number Fraction
radius (nm)
0.18
0.16
0.14
0.12
0.1
0.08
0.06
0.04
0.02
0
1
10
