# Supplementary material for: Nanoparticle size distribution quantification: results of a small-angle X-ray scattering inter-laboratory comparison
Source: J Appl Crystallogr. 2017 Aug 18;50(Pt 5):1280–8. doi: 10.1107/S160057671701010X (PMC5627679; doi:10.1107/S160057671701010X)
Supplement: Supplementary file 3 [file j-50-01280-sup2.zip › RRAnonData/csv/S53_2016-12-03_09-25-00/S53_2016-12-03_09-25-00.pdf]

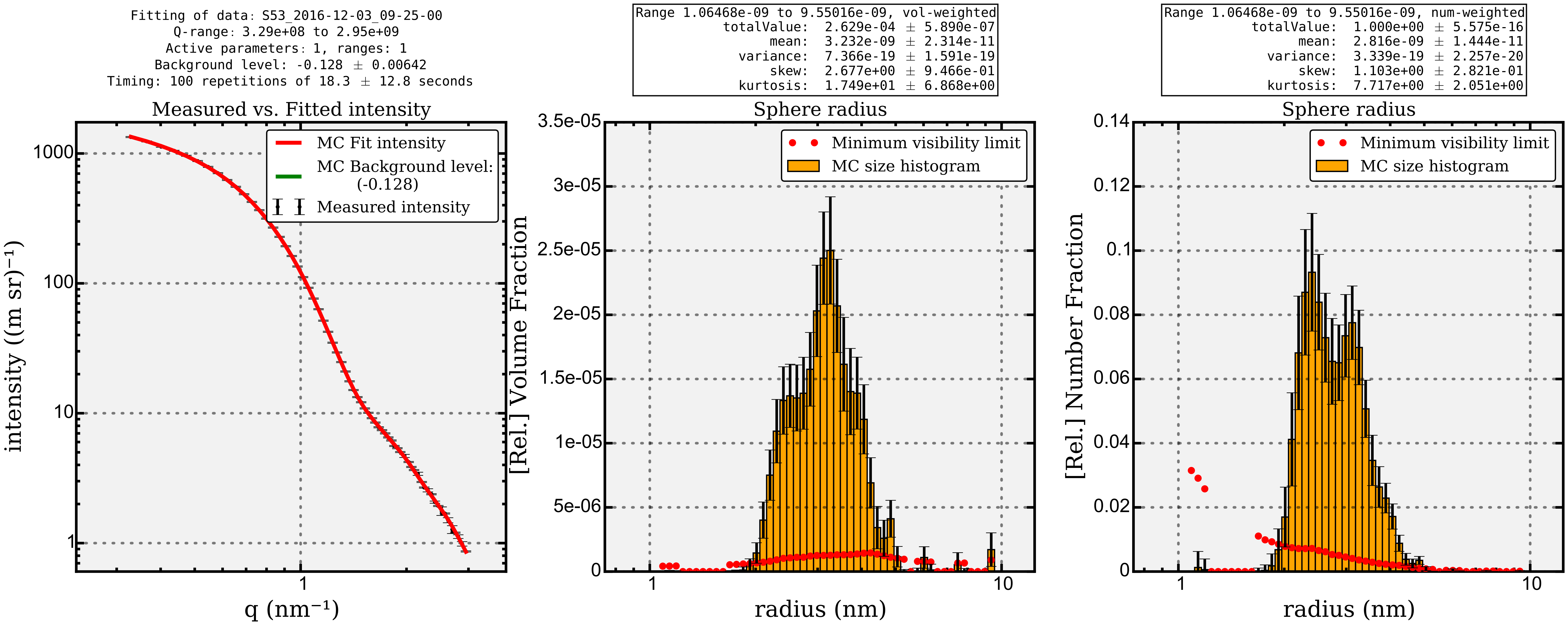

Fitting of data: S53_2016-12-03_09-25-00
Q-range: 3.29e+08 to 2.95e+09
Active parameters: 1, ranges: 1
Background level: -0.128 ± 0.00642
Timing: 100 repetitions of 18.3 ± 12.8 seconds
Measured vs. Fitted intensity
MC Fit intensity
MC Background level: (-0.128)
Measured intensity
intensity ((m sr)⁻¹)
q (nm⁻¹)
Range 1.06468e-09 to 9.55016e-09, vol-weighted
totalValue: 2.629e-04 ± 5.890e-07
mean: 3.232e-09 ± 2.314e-11
variance: 7.366e-19 ± 1.591e-19
skew: 2.677e+00 ± 9.466e-01
kurtosis: 1.749e+01 ± 6.868e+00
Sphere radius
Minimum visibility limit
MC size histogram
[Rel.] Volume Fraction
radius (nm)
Range 1.06468e-09 to 9.55016e-09, num-weighted
totalValue: 1.000e+00 ± 5.575e-16
mean: 2.816e-09 ± 1.444e-11
variance: 3.339e-19 ± 2.257e-20
skew: 1.103e+00 ± 2.821e-01
kurtosis: 7.717e+00 ± 2.051e+00
Sphere radius
Minimum visibility limit
MC size histogram
[Rel.] Number Fraction
radius (nm)
