# Supplementary material for: Nanoparticle size distribution quantification: results of a small-angle X-ray scattering inter-laboratory comparison
Source: J Appl Crystallogr. 2017 Aug 18;50(Pt 5):1280–8. doi: 10.1107/S160057671701010X (PMC5627679; doi:10.1107/S160057671701010X)
Supplement: Supplementary file 3 [file j-50-01280-sup2.zip › RRAnonData/csv/S54_2016-12-03_09-36-41/S54_2016-12-03_09-36-41.pdf]

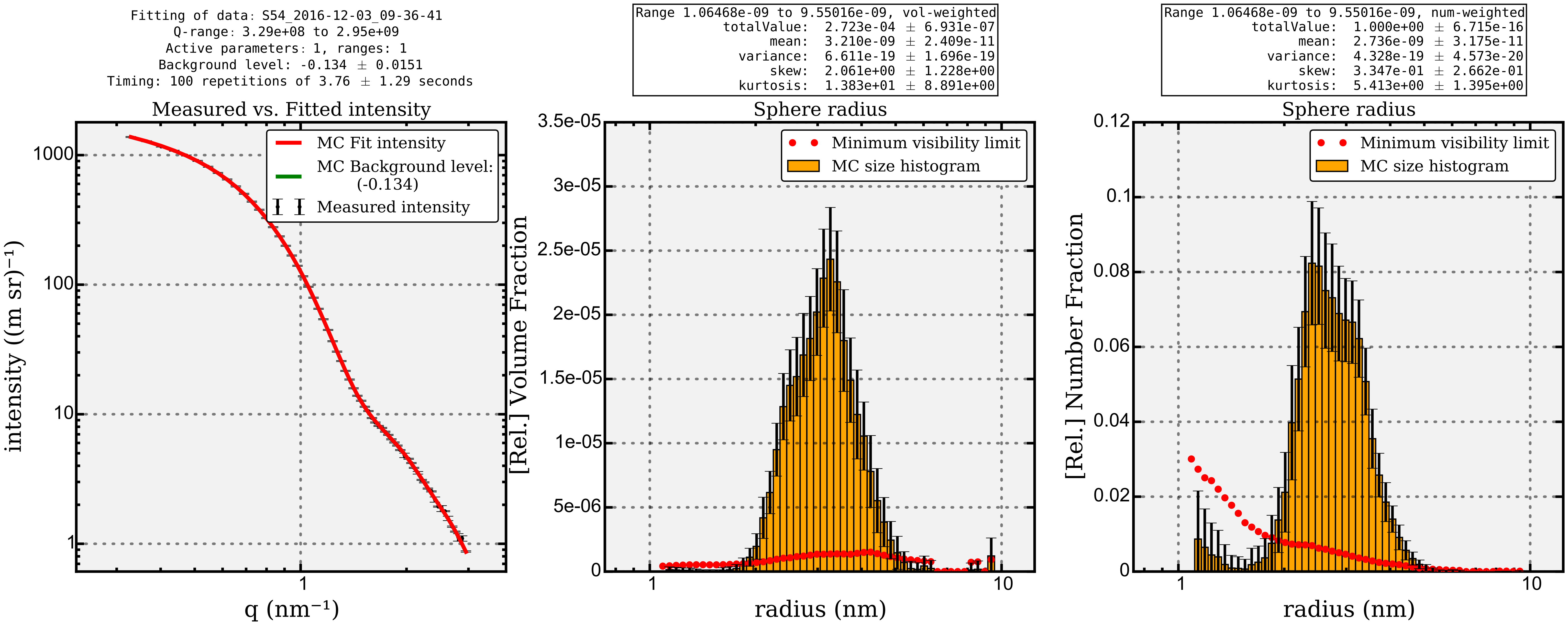

Fitting of data: S54_2016-12-03_09-36-41
Q-range: 3.29e+08 to 2.95e+09
Active parameters: 1, ranges: 1
Background level: -0.134 ± 0.0151
Timing: 100 repetitions of 3.76 ± 1.29 seconds
Measured vs. Fitted intensity
MC Fit intensity
MC Background level: (-0.134)
Measured intensity
intensity ((m sr)$^{-1}$)
q (nm^{-1})
Range 1.06468e-09 to 9.55016e-09, vol-weighted
totalValue: 2.723e-04 ± 6.931e-07
mean: 3.210e-09 ± 2.409e-11
variance: 6.611e-19 ± 1.696e-19
skew: 2.061e+00 ± 1.228e+00
kurtosis: 1.383e+01 ± 8.891e+00
Sphere radius
Minimum visibility limit
MC size histogram
[Rel.] Volume Fraction
radius (nm)
Range 1.06468e-09 to 9.55016e-09, num-weighted
totalValue: 1.000e+00 ± 6.715e-16
mean: 2.736e-09 ± 3.175e-11
variance: 4.328e-19 ± 4.573e-20
skew: 3.347e-01 ± 2.662e-01
kurtosis: 5.413e+00 ± 1.395e+00
Sphere radius
Minimum visibility limit
MC size histogram
[Rel.] Number Fraction
radius (nm)
