# Supplementary material for: Nanoparticle size distribution quantification: results of a small-angle X-ray scattering inter-laboratory comparison
Source: J Appl Crystallogr. 2017 Aug 18;50(Pt 5):1280–8. doi: 10.1107/S160057671701010X (PMC5627679; doi:10.1107/S160057671701010X)
Supplement: Supplementary file 3 [file j-50-01280-sup2.zip › RRAnonData/csv/S55_2016-12-03_09-44-49/S55_2016-12-03_09-44-49.pdf]

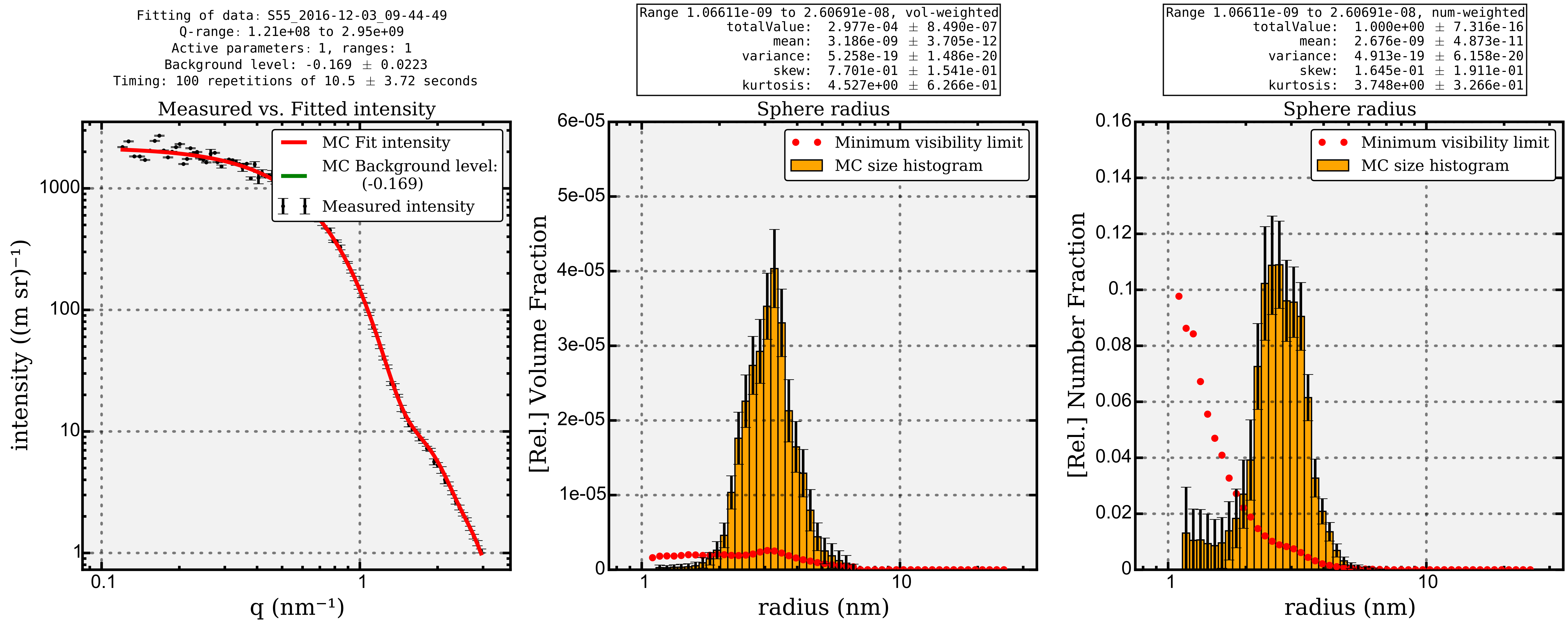

Fitting of data: S55_2016-12-03_09-44-49
Q-range: 1.21e+08 to 2.95e+09
Active parameters: 1, ranges: 1
Background level: -0.169 ± 0.0223
Timing: 100 repetitions of 10.5 ± 3.72 seconds
Measured vs. Fitted intensity
MC Fit intensity
MC Background level: (-0.169)
Measured intensity
intensity ((m sr)$^{-1}$)
q (nm^{-1})
1000
100
10
1
0.1
1
Range 1.06611e-09 to 2.60691e-08, vol-weighted
totalValue: 2.977e-04 ± 8.490e-07
mean: 3.186e-09 ± 3.705e-12
variance: 5.258e-19 ± 1.486e-20
skew: 7.701e-01 ± 1.541e-01
kurtosis: 4.527e+00 ± 6.266e-01
Sphere radius
Minimum visibility limit
MC size histogram
[Rel.] Volume Fraction
radius (nm)
6e-05
5e-05
4e-05
3e-05
2e-05
1e-05
0
1
10
Range 1.06611e-09 to 2.60691e-08, num-weighted
totalValue: 1.000e+00 ± 7.316e-16
mean: 2.676e-09 ± 4.873e-11
variance: 4.913e-19 ± 6.158e-20
skew: 1.645e-01 ± 1.911e-01
kurtosis: 3.748e+00 ± 3.266e-01
Sphere radius
Minimum visibility limit
MC size histogram
[Rel.] Number Fraction
radius (nm)
0.16
0.14
0.12
0.1
0.08
0.06
0.04
0.02
0
1
10
