# Supplementary material for: Nanoparticle size distribution quantification: results of a small-angle X-ray scattering inter-laboratory comparison
Source: J Appl Crystallogr. 2017 Aug 18;50(Pt 5):1280–8. doi: 10.1107/S160057671701010X (PMC5627679; doi:10.1107/S160057671701010X)
Supplement: Supplementary file 3 [file j-50-01280-sup2.zip › RRAnonData/csv/S56_2016-12-03_10-02-41/S56_2016-12-03_10-02-41.pdf]

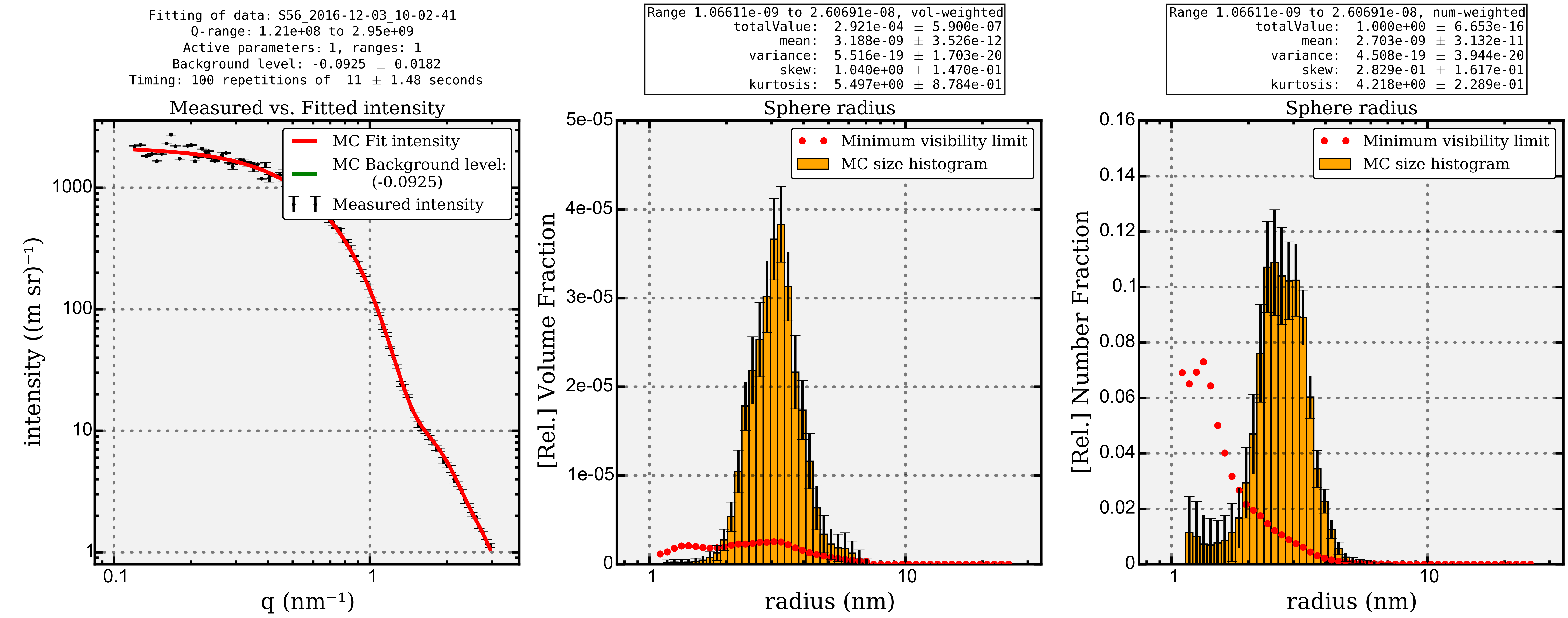
Fitting of data: S56_2016-12-03_10-02-41
Q-range: 1.21e+08 to 2.95e+09
Active parameters: 1, ranges: 1
Background level: -0.0925 ± 0.0182
Timing: 100 repetitions of 11 ± 1.48 seconds
Measured vs. Fitted intensity
MC Fit intensity
MC Background level: (-0.0925)
Measured intensity
intensity ((m sr)⁻¹)
q (nm⁻¹)
Range 1.06611e-09 to 2.60691e-08, vol-weighted
totalValue: 2.921e-04 ± 5.900e-07
mean: 3.188e-09 ± 3.526e-12
variance: 5.516e-19 ± 1.703e-20
skew: 1.040e+00 ± 1.470e-01
kurtosis: 5.497e+00 ± 8.784e-01
Sphere radius
Minimum visibility limit
MC size histogram
[Rel.] Volume Fraction
radius (nm)
Range 1.06611e-09 to 2.60691e-08, num-weighted
totalValue: 1.000e+00 ± 6.653e-16
mean: 2.703e-09 ± 3.132e-11
variance: 4.508e-19 ± 3.944e-20
skew: 2.829e-01 ± 1.617e-01
kurtosis: 4.218e+00 ± 2.289e-01
Sphere radius
Minimum visibility limit
MC size histogram
[Rel.] Number Fraction
radius (nm)
