# Supplementary material for: Nanoparticle size distribution quantification: results of a small-angle X-ray scattering inter-laboratory comparison
Source: J Appl Crystallogr. 2017 Aug 18;50(Pt 5):1280–8. doi: 10.1107/S160057671701010X (PMC5627679; doi:10.1107/S160057671701010X)
Supplement: Supplementary file 3 [file j-50-01280-sup2.zip › RRAnonData/csv/S57_2016-12-02_21-42-46/S57_2016-12-02_21-42-46.pdf]

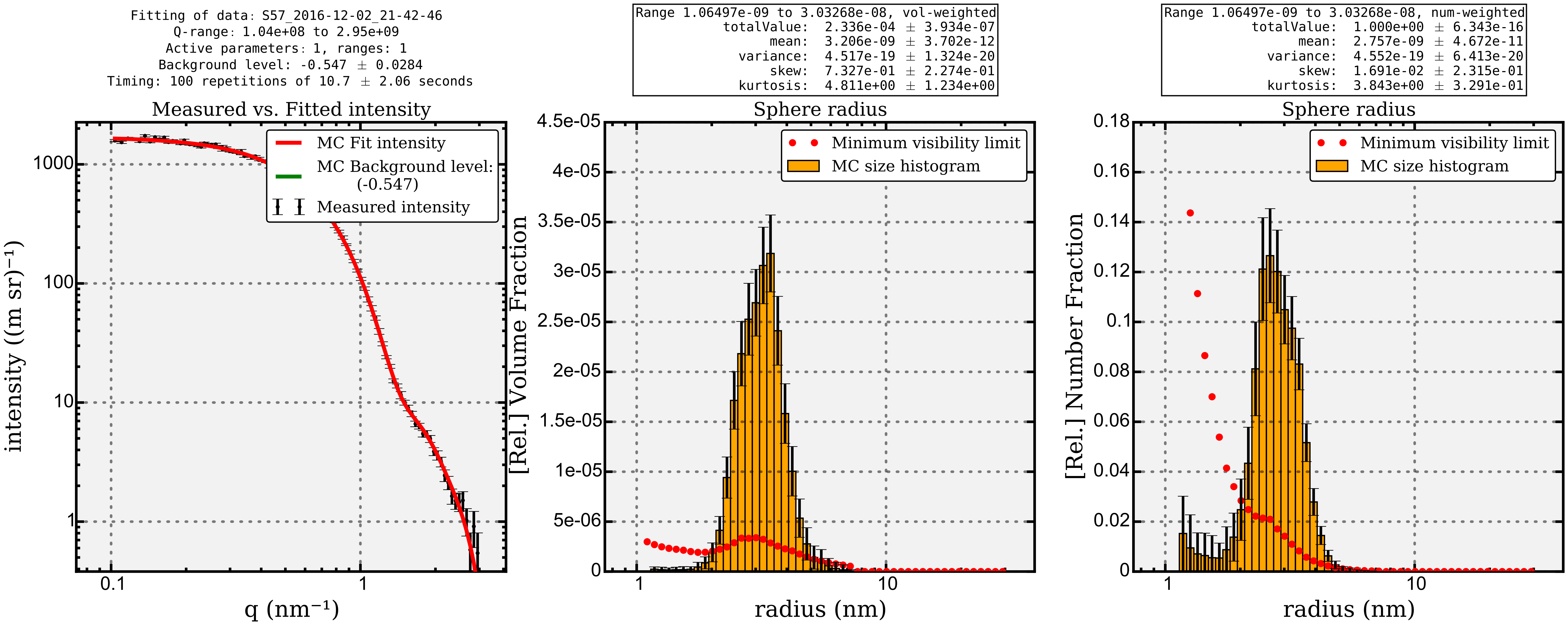
Fitting of data: S57_2016-12-02_21-42-46
Q-range: 1.04e+08 to 2.95e+09
Active parameters: 1, ranges: 1
Background level: -0.547 ± 0.0284
Timing: 100 repetitions of 10.7 ± 2.06 seconds
Measured vs. Fitted intensity
MC Fit intensity
MC Background level: (-0.547)
Measured intensity
intensity ((m sr)⁻¹)
q (nm⁻¹)
Range 1.06497e-09 to 3.03268e-08, vol-weighted
totalValue: 2.336e-04 ± 3.934e-07
mean: 3.206e-09 ± 3.702e-12
variance: 4.517e-19 ± 1.324e-20
skew: 7.327e-01 ± 2.274e-01
kurtosis: 4.811e+00 ± 1.234e+00
Sphere radius
Minimum visibility limit
MC size histogram
[Rel.] Volume Fraction
radius (nm)
Range 1.06497e-09 to 3.03268e-08, num-weighted
totalValue: 1.000e+00 ± 6.343e-16
mean: 2.757e-09 ± 4.672e-11
variance: 4.552e-19 ± 6.413e-20
skew: 1.691e-02 ± 2.315e-01
kurtosis: 3.843e+00 ± 3.291e-01
Sphere radius
Minimum visibility limit
MC size histogram
[Rel.] Number Fraction
radius (nm)
