# Supplementary material for: Nanoparticle size distribution quantification: results of a small-angle X-ray scattering inter-laboratory comparison
Source: J Appl Crystallogr. 2017 Aug 18;50(Pt 5):1280–8. doi: 10.1107/S160057671701010X (PMC5627679; doi:10.1107/S160057671701010X)
Supplement: Supplementary file 3 [file j-50-01280-sup2.zip › RRAnonData/csv/S58_2016-12-03_09-44-48/S58_2016-12-03_09-44-48.pdf]

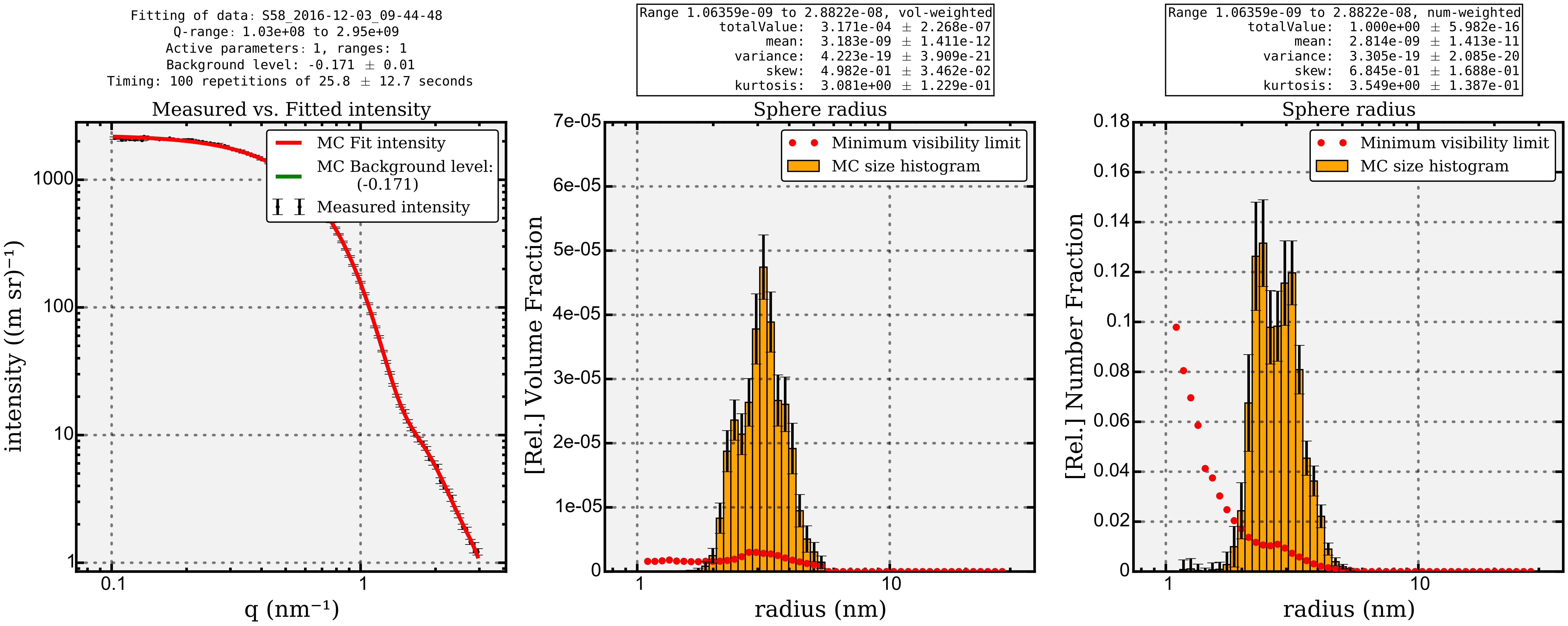

Fitting of data: S58_2016-12-03_09-44-48
Q-range: 1.03e+08 to 2.95e+09
Active parameters: 1, ranges: 1
Background level: -0.171 ± 0.01
Timing: 100 repetitions of 25.8 ± 12.7 seconds
Measured vs. Fitted intensity
MC Fit intensity
MC Background level: (-0.171)
Measured intensity
intensity ((m sr)⁻¹)
q (nm⁻¹)
Range 1.06359e-09 to 2.8822e-08, vol-weighted
totalValue: 3.171e-04 ± 2.268e-07
mean: 3.183e-09 ± 1.411e-12
variance: 4.223e-19 ± 3.909e-21
skew: 4.982e-01 ± 3.462e-02
kurtosis: 3.081e+00 ± 1.229e-01
Sphere radius
Minimum visibility limit
MC size histogram
[Rel.] Volume Fraction
radius (nm)
Range 1.06359e-09 to 2.8822e-08, num-weighted
totalValue: 1.000e+00 ± 5.982e-16
mean: 2.814e-09 ± 1.413e-11
variance: 3.305e-19 ± 2.085e-20
skew: 6.845e-01 ± 1.688e-01
kurtosis: 3.549e+00 ± 1.387e-01
Sphere radius
Minimum visibility limit
MC size histogram
[Rel.] Number Fraction
radius (nm)
