# Supplementary material for: Nanoparticle size distribution quantification: results of a small-angle X-ray scattering inter-laboratory comparison
Source: J Appl Crystallogr. 2017 Aug 18;50(Pt 5):1280–8. doi: 10.1107/S160057671701010X (PMC5627679; doi:10.1107/S160057671701010X)
Supplement: Supplementary file 3 [file j-50-01280-sup2.zip › RRAnonData/csv/S62_2016-12-03_09-57-27/S62_2016-12-03_09-57-27.pdf]

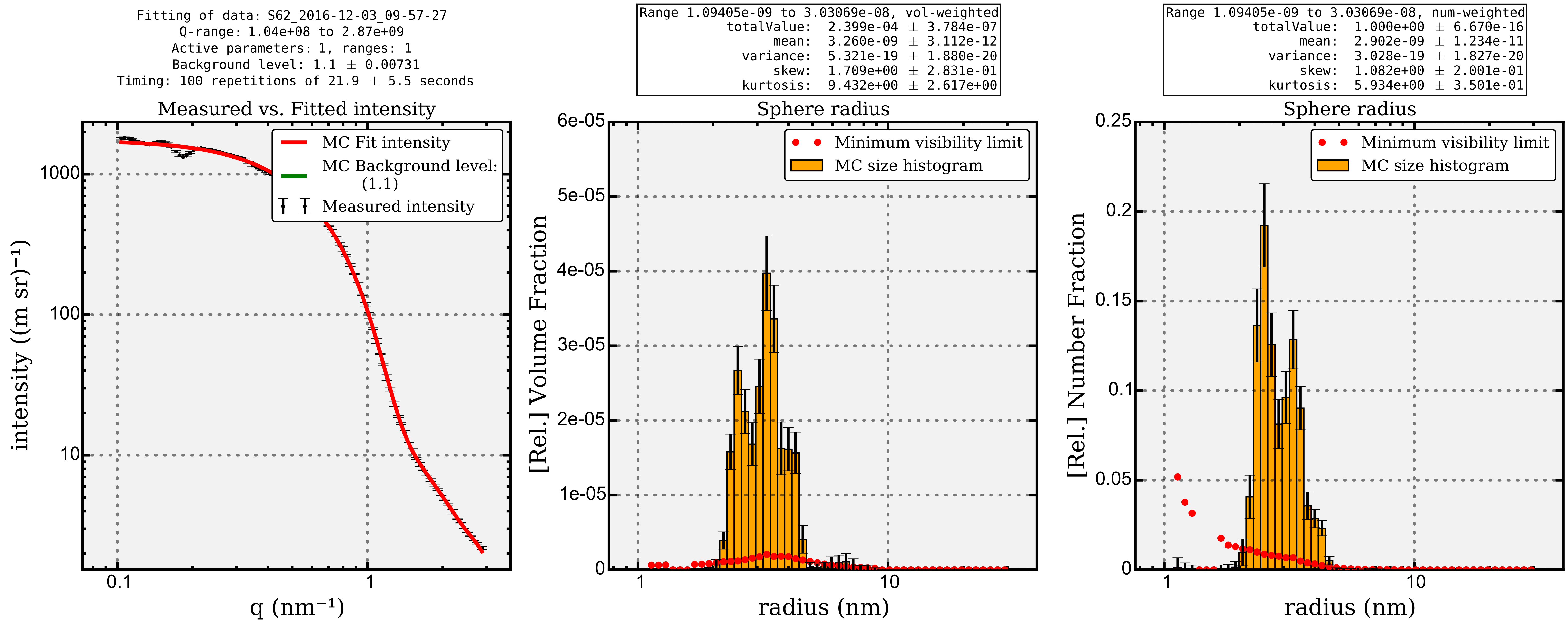
Fitting of data: S62_2016-12-03_09-57-27
Q-range: 1.04e+08 to 2.87e+09
Active parameters: 1, ranges: 1
Background level: 1.1 ± 0.00731
Timing: 100 repetitions of 21.9 ± 5.5 seconds
Measured vs. Fitted intensity
MC Fit intensity
MC Background level: (1.1)
Measured intensity
intensity ((m sr)⁻¹)
1000
100
10
0.1
1
q (nm⁻¹)
Range 1.09405e-09 to 3.03069e-08, vol-weighted
totalValue: 2.399e-04 ± 3.784e-07
mean: 3.260e-09 ± 3.112e-12
variance: 5.321e-19 ± 1.880e-20
skew: 1.709e+00 ± 2.831e-01
kurtosis: 9.432e+00 ± 2.617e+00
Sphere radius
Minimum visibility limit
MC size histogram
[Rel.] Volume Fraction
6e-05
5e-05
4e-05
3e-05
2e-05
1e-05
0
1
10
radius (nm)
Range 1.09405e-09 to 3.03069e-08, num-weighted
totalValue: 1.000e+00 ± 6.670e-16
mean: 2.902e-09 ± 1.234e-11
variance: 3.028e-19 ± 1.827e-20
skew: 1.082e+00 ± 2.001e-01
kurtosis: 5.934e+00 ± 3.501e-01
Sphere radius
Minimum visibility limit
MC size histogram
[Rel.] Number Fraction
0.25
0.2
0.15
0.1
0.05
0
1
10
radius (nm)
