# Supplementary material for: Nanoparticle size distribution quantification: results of a small-angle X-ray scattering inter-laboratory comparison
Source: J Appl Crystallogr. 2017 Aug 18;50(Pt 5):1280–8. doi: 10.1107/S160057671701010X (PMC5627679; doi:10.1107/S160057671701010X)
Supplement: Supplementary file 3 [file j-50-01280-sup2.zip › RRAnonData/csv/S63_2016-12-03_10-34-13/S63_2016-12-03_10-34-13.pdf]

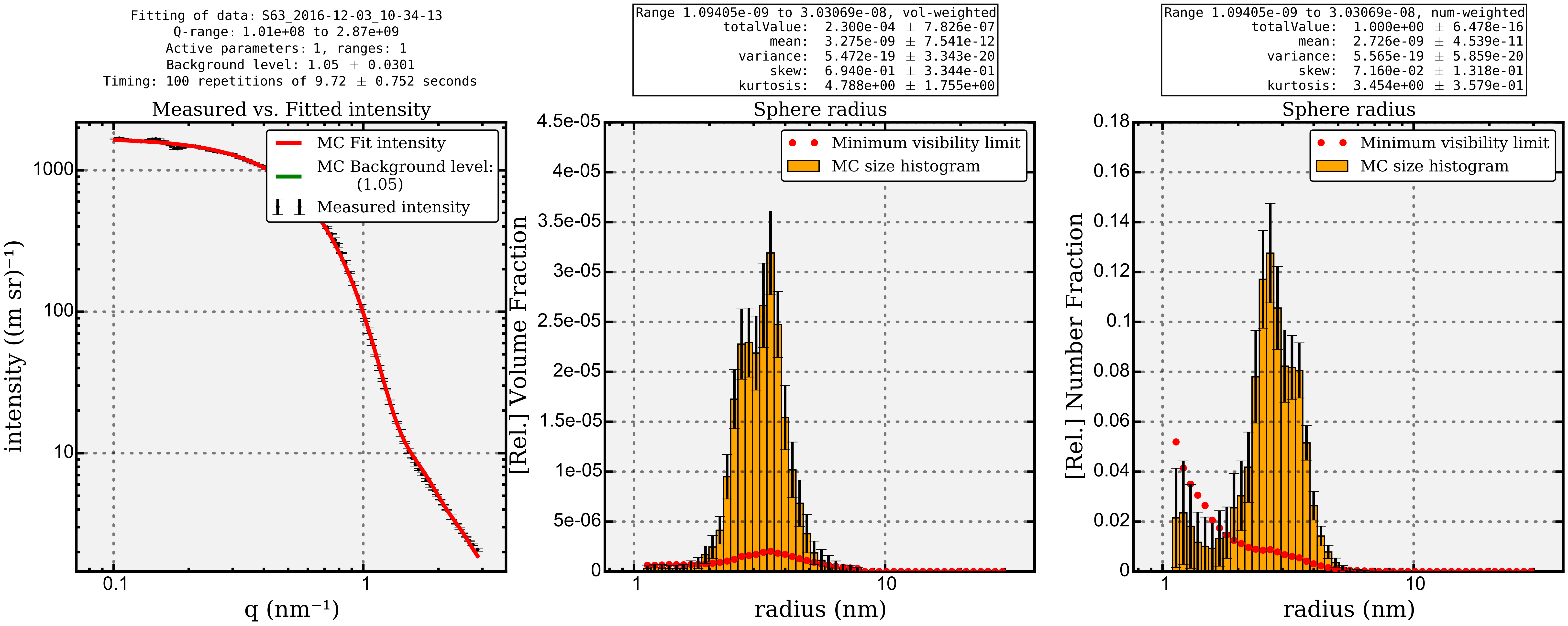
Fitting of data: S63_2016-12-03_10-34-13
Q-range: 1.01e+08 to 2.87e+09
Active parameters: 1, ranges: 1
Background level: 1.05 ± 0.0301
Timing: 100 repetitions of 9.72 ± 0.752 seconds
Measured vs. Fitted intensity
MC Fit intensity
MC Background level: (1.05)
Measured intensity
intensity ((m sr)$^{-1}$)
1000
100
10
0.1
1
q (nm^{-1})
Range 1.09405e-09 to 3.03069e-08, vol-weighted
totalValue: 2.300e-04 ± 7.826e-07
mean: 3.275e-09 ± 7.541e-12
variance: 5.472e-19 ± 3.343e-20
skew: 6.940e-01 ± 3.344e-01
kurtosis: 4.788e+00 ± 1.755e+00
Sphere radius
Minimum visibility limit
MC size histogram
[Rel.] Volume Fraction
4.5e-05
4e-05
3.5e-05
3e-05
2.5e-05
2e-05
1.5e-05
1e-05
5e-06
0
1
10
radius (nm)
Range 1.09405e-09 to 3.03069e-08, num-weighted
totalValue: 1.000e+00 ± 6.478e-16
mean: 2.726e-09 ± 4.539e-11
variance: 5.565e-19 ± 5.859e-20
skew: 7.160e-02 ± 1.318e-01
kurtosis: 3.454e+00 ± 3.579e-01
Sphere radius
Minimum visibility limit
MC size histogram
[Rel.] Number Fraction
0.18
0.16
0.14
0.12
0.1
0.08
0.06
0.04
0.02
0
1
10
radius (nm)
