# Supplementary material for: Nanoparticle size distribution quantification: results of a small-angle X-ray scattering inter-laboratory comparison
Source: J Appl Crystallogr. 2017 Aug 18;50(Pt 5):1280–8. doi: 10.1107/S160057671701010X (PMC5627679; doi:10.1107/S160057671701010X)
Supplement: Supplementary file 3 [file j-50-01280-sup2.zip › RRAnonData/csv/S6_2016-12-02_20-35-01/S6_2016-12-02_20-35-01.pdf]

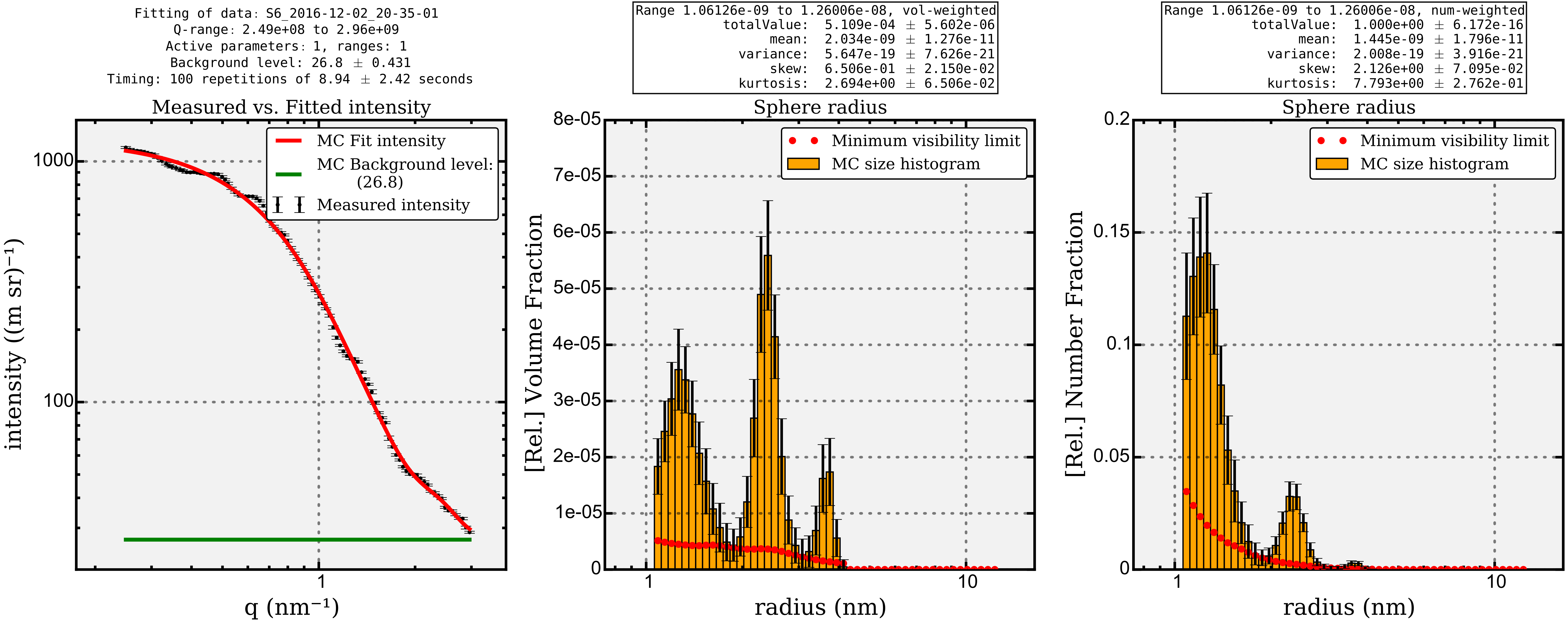
Fitting of data: S6_2016-12-02_20-35-01
Q-range: 2.49e+08 to 2.96e+09
Active parameters: 1, ranges: 1
Background level: 26.8 ± 0.431
Timing: 100 repetitions of 8.94 ± 2.42 seconds
Measured vs. Fitted intensity
MC Fit intensity
MC Background level: (26.8)
Measured intensity
1000
100
intensity ((m sr)⁻¹)
1
q (nm⁻¹)
Range 1.06126e-09 to 1.26006e-08, vol-weighted
totalValue: 5.109e-04 ± 5.602e-06
mean: 2.034e-09 ± 1.276e-11
variance: 5.647e-19 ± 7.626e-21
skew: 6.506e-01 ± 2.150e-02
kurtosis: 2.694e+00 ± 6.506e-02
Sphere radius
Minimum visibility limit
MC size histogram
8e-05
7e-05
6e-05
5e-05
4e-05
3e-05
2e-05
1e-05
0
[Rel.] Volume Fraction
1
10
radius (nm)
Range 1.06126e-09 to 1.26006e-08, num-weighted
totalValue: 1.000e+00 ± 6.172e-16
mean: 1.445e-09 ± 1.796e-11
variance: 2.008e-19 ± 3.916e-21
skew: 2.126e+00 ± 7.095e-02
kurtosis: 7.793e+00 ± 2.762e-01
Sphere radius
Minimum visibility limit
MC size histogram
0.2
0.15
0.1
0.05
0
[Rel.] Number Fraction
1
10
radius (nm)
