# Supplementary material for: Nanoparticle size distribution quantification: results of a small-angle X-ray scattering inter-laboratory comparison
Source: J Appl Crystallogr. 2017 Aug 18;50(Pt 5):1280–8. doi: 10.1107/S160057671701010X (PMC5627679; doi:10.1107/S160057671701010X)
Supplement: Supplementary file 3 [file j-50-01280-sup2.zip › RRAnonData/Curves.pdf]

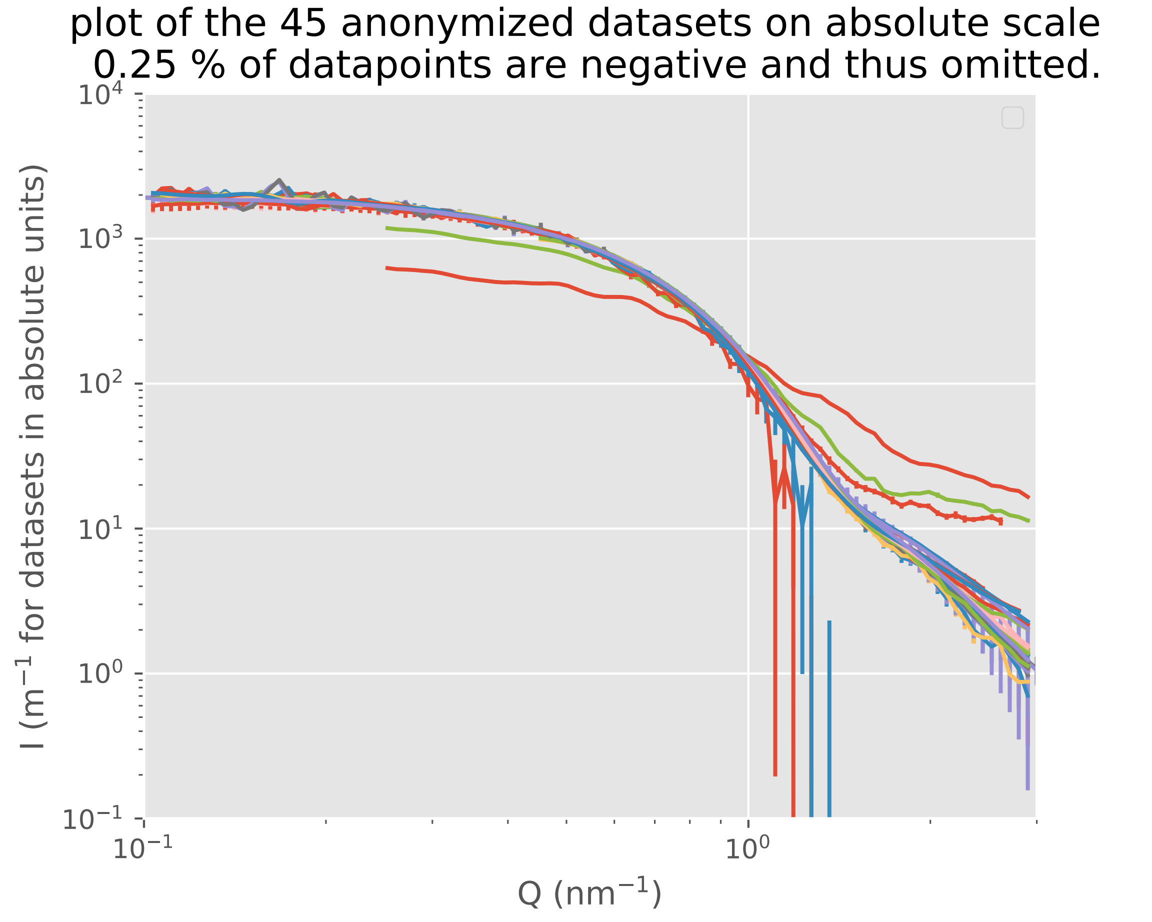

plot of the 45 anonymized datasets on absolute scale
0.25 % of datapoints are negative and thus omitted.
10^4
10^3
10^2
10^1
10^0
10^{-1}
10^{-1}
10^0
I (m^{-1} for datasets in absolute units)
Q (nm^{-1})
