# Supplementary material for: Nanoparticle size distribution quantification: results of a small-angle X-ray scattering inter-laboratory comparison
Source: J Appl Crystallogr. 2017 Aug 18;50(Pt 5):1280–8. doi: 10.1107/S160057671701010X (PMC5627679; doi:10.1107/S160057671701010X)
Supplement: Supplementary file 3 [file j-50-01280-sup2.zip › RRAnonData/deviation.pdf]

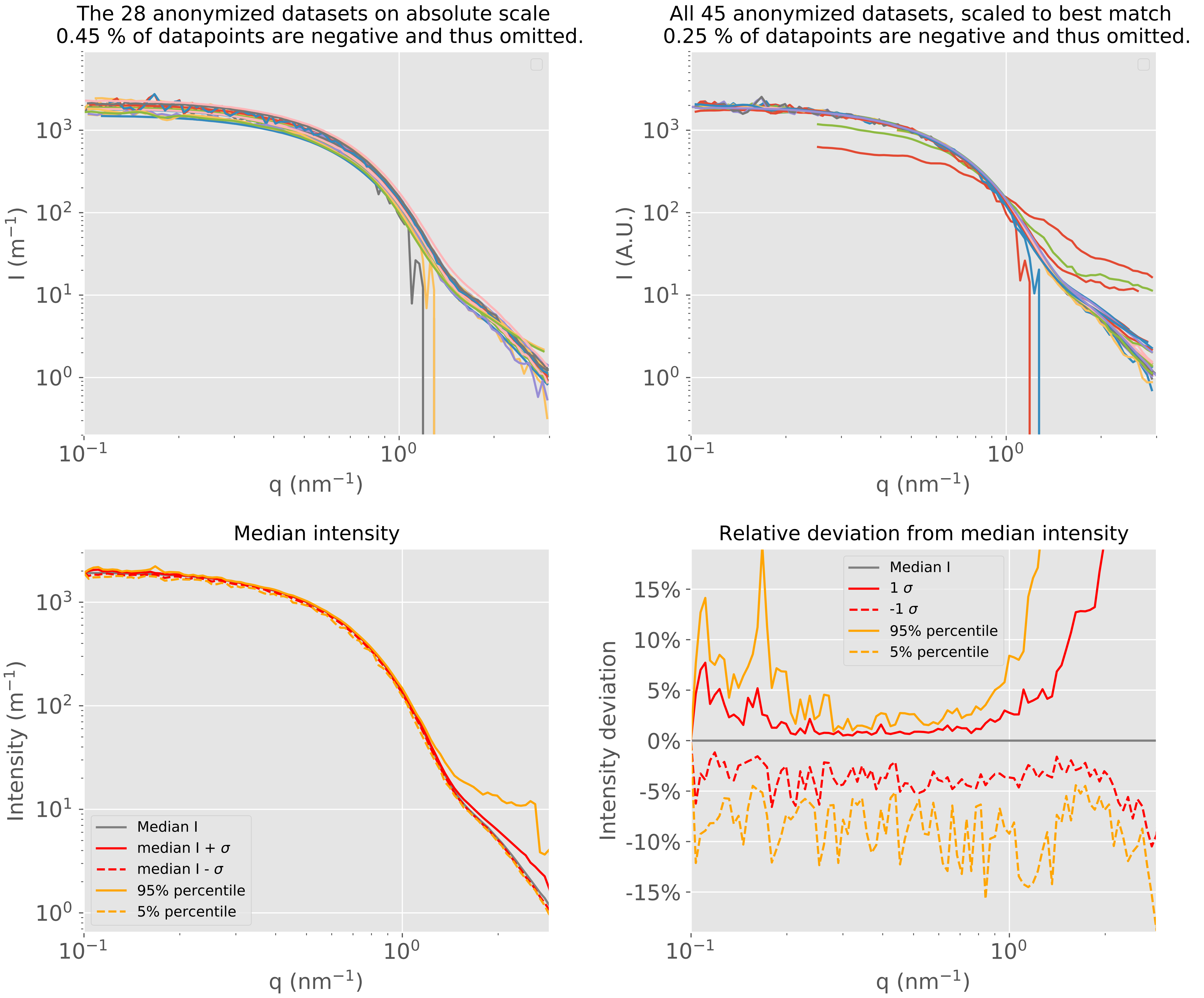

The 28 anonymized datasets on absolute scale
0.45 % of datapoints are negative and thus omitted.
I (m^{-1})
q (nm^{-1})
All 45 anonymized datasets, scaled to best match
0.25 % of datapoints are negative and thus omitted.
I (A.U.)
q (nm^{-1})
Median intensity
Intensity (m^{-1})
Median I
median I + σ
median I - σ
95% percentile
5% percentile
q (nm^{-1})
Relative deviation from median intensity
Intensity deviation
Median I
1 σ
-1 σ
95% percentile
5% percentile
q (nm^{-1})
