# Supplementary material for: Nanoparticle size distribution quantification: results of a small-angle X-ray scattering inter-laboratory comparison
Source: J Appl Crystallogr. 2017 Aug 18;50(Pt 5):1280–8. doi: 10.1107/S160057671701010X (PMC5627679; doi:10.1107/S160057671701010X)
Supplement: Supplementary file 3 [file j-50-01280-sup2.zip › RRAnonData/McSASDist.pdf]

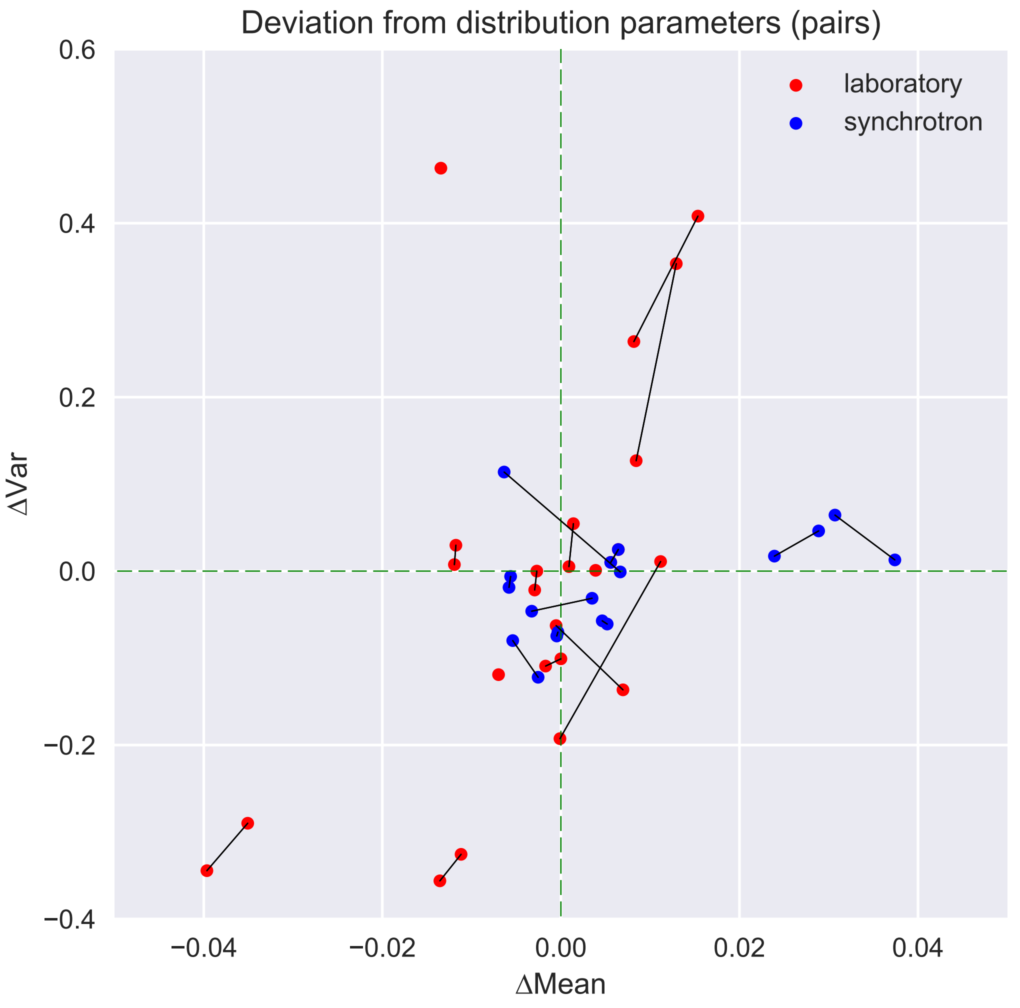

Deviation from distribution parameters (pairs)
laboratory
synchrotron
0.6
0.4
0.2
0.0
−0.2
−0.4
−0.04
−0.02
0.00
0.02
0.04
ΔMean
ΔVar
